# Randomised Controlled Feasibility Trial of an Evidence-Informed Behavioural Intervention for Obese Adults with Additional Risk Factors

**DOI:** 10.1371/journal.pone.0023040

**Published:** 2011-08-29

**Authors:** Falko F. Sniehotta, Stephan U. Dombrowski, Alison Avenell, Marie Johnston, Suzanne McDonald, Peter Murchie, Craig R. Ramsay, Kim Robertson, Vera Araujo-Soares

**Affiliations:** 1 Health Psychology Group, Institute of Health and Society, Newcastle University, Newcastle upon Tyne, United Kingdom; 2 Fuse: The UK Centre for Translational Research in Public Health, Newcastle University, Newcastle upon Tyne, United Kingdom; 3 Health Services Research Unit, University of Aberdeen, Aberdeen, United Kingdom; 4 College of Life Sciences and Medicine, University of Aberdeen, Aberdeen, United Kingdom; 5 Centre of Academic Primary Care, University of Aberdeen, Aberdeen, United Kingdom; Finnish Institute of Occupational Health, Finland

## Abstract

**Background:**

Interventions for dietary and physical activity changes in obese adults may be less effective for participants with additional obesity-related risk factors and co-morbidities than for otherwise healthy individuals. This study aimed to test the feasibility and acceptability of the recruitment, allocation, measurement, retention and intervention procedures of a randomised controlled trial of an intervention to improve physical activity and dietary practices amongst obese adults with additional obesity related risk factors.

**Method:**

Pilot single centre open-labelled outcome assessor-blinded randomised controlled trial of obese (Body Mass Index (BMI)≥30 kg/m2) adults (age≥18 y) with obesity related co-morbidities such as type 2 diabetes, impaired glucose tolerance or hypertension. Participants were randomly allocated to a manual-based group intervention or a leaflet control condition in accordance to a 2∶1 allocation ratio. Primary outcome was acceptability and feasibility of trial procedures, secondary outcomes included measures of body composition, physical activity, food intake and psychological process measures.

**Results:**

Out of 806 potentially eligible individuals identified through list searches in two primary care general medical practices N = 81 participants (63% female; mean-age = 56.56(11.44); mean-BMI = 36.73(6.06)) with 2.35(1.47) co-morbidities were randomised. Scottish Index of Multiple Deprivation (SIMD) was the only significant predictor of providing consent to take part in the study (higher chances of consent for invitees with lower levels of deprivation). Participant flowcharts, qualitative and quantitative feedback suggested good acceptance and feasibility of intervention procedures but 34.6% of randomised participants were lost to follow-up due to overly high measurement burden and sub-optimal retention procedures. Participants in the intervention group showed positive trends for most psychological, behavioural and body composition outcomes.

**Conclusions:**

The intervention procedures were found to be acceptable and feasible. Attrition rates were unacceptably high and areas for improvements of trial procedures were identified.

**Trial Registration:**

Controlled-Trials.com ISRCTN90101501

## Introduction

About two thirds of adults in the US and UK are overweight (BMI, 25 to 29.9 kg/m^2^) or obese (BMI≥30 kg/m^2^) and at least one quarter are obese [1], [2], [3]. Excess body weight is associated with a cluster of metabolic and cardiovascular risk factors [4] and there is compelling evidence linking obesity to the risk for cardiovascular disease [5], [6], type 2 diabetes mellitus [7], [8], cancer [9], [10] and other conditions [11] resulting in considerable disability [12], premature mortality [13] and health service costs [14]. Obese individuals with additional obesity-related risk factors (e.g., impaired glucose tolerance) and secondary conditions (e.g., Type 2 diabetes mellitus) have a higher risk for further ill health, consume more health service costs and would therefore benefit most from effective interventions targeting food intake, physical activity and weight loss.

A recent systematic review of 44 Randomised Controlled Trials (RCTs) of behavioural interventions for obese adults with additional obesity-related risk factors and/or co-morbidities showed that interventions targeting both, dietary and physical activity changes, result in consistent improvements in weight and weight-related cardiovascular disease risk factors [15]. However, the effects of these interventions are smaller compared with systematic reviews not limiting study inclusion to participants with additional risk factors [16], [17]. This suggests that achieving behaviour change, consequent weight loss and risk reduction is more difficult amongst individuals who have already developed additional risk factors and obesity related disease.

### Intervention Development

Interventions for health should be based on best evidence and theory to optimise their effectiveness and understand how and why interventions do or do not work [18], [19], [20], [21]. However, previous weight loss interventions have rarely been systematically developed and published reports do not describe if and how evidence or relevant theory informed the content or delivery of weight loss treatment [22]. The UK National Institute for Health and Clinical Excellence obesity guidelines suggest that obesity research needs to identify what ‘elements make an intervention effective and sustainable’ p. 63 [23]. The current paper reports a randomised feasibility trial of an intervention resulting from a systematic, evidence-informed development process in line with recent guidelines and methodologies for developing complex interventions for health [18], [19], [24].

We conducted a systematic review of 44 RCTs of behavioural interventions targeting dietary and/or physical activity changes in obese adults with additional obesity-related co-morbidities (e.g., Type 2 diabetes mellitus or cardiovascular disease) or risk factors for co-morbidities (e.g., impaired glucose tolerance or hypercholesterolaemia), representing a total of more than 10,000 participants [15]. Each of the usually complex interventions included in the review was thoroughly characterised in terms of a) modes of delivery (e.g., face-to-face versus computer delivered, individual versus group setting), b) use of theory in defining the content and intermediate targets of intervention techniques and/or the combination and sequence of their delivery and c) the use of behaviour change techniques (e.g., goal setting, provision of knowledge, etc) based on a recent reliable taxonomy of behaviour change techniques [25], [26]. Meta regression was used to identify intervention features associated with intervention effectiveness [27]. The review found that neither the mode of intervention delivery nor the timing of the active intervention period were associated with effectiveness in achieving weight loss, whereas interventions with more frequent contact with participants achieved higher weight loss. Studies recruiting from clinical settings were less effective than studies recruiting from community settings and marginally less effective than studies recruiting through general practice. No significant associations between the *number* of behaviour change techniques utilised and effectiveness were found. However, studies utilising techniques of *intention formation/goal setting*, *self-monitoring of behaviour*, *action planning*, *barrier identification/coping planning*, *review of behavioural goals*, *prompting practice*, *planning contingent rewards*; *relapse prevention* were more effective in achieving weight loss. Moreover, the review found that studies using behaviour change techniques congruent with Self-Regulation Theory [28], [29], Social Cognitive Theory [30], Social Comparison Theory [31] were more effective than studies using other techniques, suggesting these theories as a useful framework for the development of a new intervention. Based on this evidence, a draft intervention manual was developed involving the clinical expertise of physicians caring for obese adults, dieticians, health/clinical psychologists, and nutritionists. Intervention materials developed were informed by publicly available materials of successful trials included in the review [32], previous RCTs of the researchers [33], [34], [35] and the UK Health Trainer manual [36]. A comprehensive training programme was developed for delivery by health professionals.

### Pilot studies

Acceptability and feasibility of the draft intervention materials and procedures were tested in an initial pilot study without outcome measurement in a group of 12 adults participating in a community-based public weight management programme where the intervention was found acceptable and popular amongst participants. Subsequently, an open, uncontrolled before-and-after pilot study with consecutive recruitment of eight small groups of users of an urban hospital-based obesity clinic (N = 74) was conducted. This open pilot allowed systematic intervention adaptation and refinement in accordance with ongoing feedback. Ongoing quantitative and qualitative assessments of acceptability and satisfaction were collected from participants and measures of acceptability and feasibility by the research nurse delivering the intervention. Overall satisfaction was 94.5% and participants showed positive changes in physical activity and weight pre-post study and trends toward improved dietary practices. Minor changes reflecting participants' and facilitators' feedback were made, further improving the intervention (e.g., materials were adapted, the duration of sessions was increased from 60 to 90 minutes and a follow-up session was added) [37].

Based on the evidence from this systematic development process, the current study aimed to test feasibility and acceptability of procedures for recruitment, allocation, measurement, retention, and for the intervention to inform a definitive RCT of this novel, systematically developed intervention for obese adults with additional obesity-related risk factors and co-morbid conditions. We pre-specified the following criteria for considering the protocol viable for a definitive RCT without modification: a) a recruitment rate of at least 10% of eligible patients (based on typical rates for study recruitment through general medical practices in the region), b) attrition rates of less than 20% (based on systematic review evidence and typical risk of attrition bias considerations [15], [38] and compliance rates (group attendance and material completion) of 60% (based on open pilot study [37]). If these targets were not met, modifications to the protocol in the light of the study's findings and potentially further pilot work would be required.

## Methods

The protocol for this trial and supporting CONSORT checklist are available as supporting information; see Checklist S1 and Protocol S1.

### Ethics

The study protocol (Protocol S1, S2, S3, S4, S5, S6, S7, S8 and S9) was approved by the North of Scotland Research Ethics Committee (REC 09/S0801/54), 28^th^ May 2009. Written informed consent was obtained from all study participants.

### Trial Design

This was a single centre, outcome assessor blinded, parallel group study with imbalanced randomisation [2∶1] conducted in Aberdeen, Scotland, UK (Trial registration: ISRCTN90101501; CONSORT checklist S1).

### Participants

Adults aged ≥18 years with a Body Mass Index (BMI) of ≥30 kg/m^2^ with at least one of the following additional risk factors/conditions: hypertension (blood pressure ≥150/90); coronary or ischaemic heart disease; chronic obstructive pulmonary disease (COPD), Type 1 or Type 2 diabetes mellitus, impaired glucose tolerance (IGT), cerebrovascular disease and arthritis. Eligible participants were identified by the Scottish Primary Care Research Network (SPCRN) through searching the list of patients registered with two primary care General Medical Practices' patient lists in September 2008. Invitation letters together with participant information materials, response slips and consent forms were sent through the practices. Exclusion criteria were: current treatment for cancer, dementia or significant psychiatric illness, inability to give informed consent, inability to comply with trial protocol (e.g. terminally ill, housebound) and insufficient language skills to complete consent procedures.

### Study setting

The Clinical Research Facility of the University of Aberdeen, set up at the central hospital site for ambulatory clinical research such as clinical trials staffed with specifically trained research nurses.

### Interventions

Participants were randomly allocated to either a face-to-face group behaviour change intervention focusing on dietary and physical activity changes for weight loss, or a control group.

### Face-to-face group intervention

This manual and material-based intervention (Intervention Manual S1, S2, S3, S4, S5 and S6) consisted of five weekly group sessions, and one follow-up session (week 8). Sessions lasted ∼90 min and were delivered by one researcher with 7 years of experience, a BA in Midwifery, a BSc in Environmental Health and an MSc in Public Health and current training in research methods and communication skills. Participants missing sessions were sent materials and details of the next meeting. Facilitator training consisted of three 4 h workshops delivered by VAS, SUD and FFS.

Intervention participants completed self-selected weekly goals between sessions, kept a behavioural diary and used a provided pedometer (HJ-113 piezoelectric pedometer-Omron Healthcare Ltd. Milton Keynes, UK). The intervention comprised of evidence-based behaviour change techniques (BCTs) [25], [26] found to be associated with effective physical activity and dietary interventions for the target population [27], including *intention formation/goal setting*, *self-monitoring of behaviour*, *action planning*, *barrier identification/coping planning*, *review of behavioural goals*, *prompting practice*, *planning contingent rewards* and *relapse prevention*. These were delivered through facilitator advice as well as paper and pencil materials used during the sessions. Participants were asked to complete and return weekly booklets which formed the basis of small group discussions with the facilitator based on the prior reading of submitted booklets.

Participants in both groups received two British Heart Foundation (BHF) booklets: a) ‘So you want to lose weight for good’- including information on portion sizes, a daily eating plan and a section to track progress, and b) ‘Get Active’, including recommendations and types of safe physical activity, outlines positive health benefits of physical activity and briefly addresses barriers.

### Control group

Participants received standard care and two BHF leaflets as described above by post.

### Measurement

Outcome and process measures were taken before randomisation (Time 1) and six months later (Time 3), using a combination of face-to-face measurement by a research nurse (anthropometric and performance measures) and postal questionnaires (self-reports of food intake, physical activity; and psychological process measures). The postal questionnaire was repeated three months following randomisation (Time 2).

### Acceptability and feasibility

**Figure 1:**
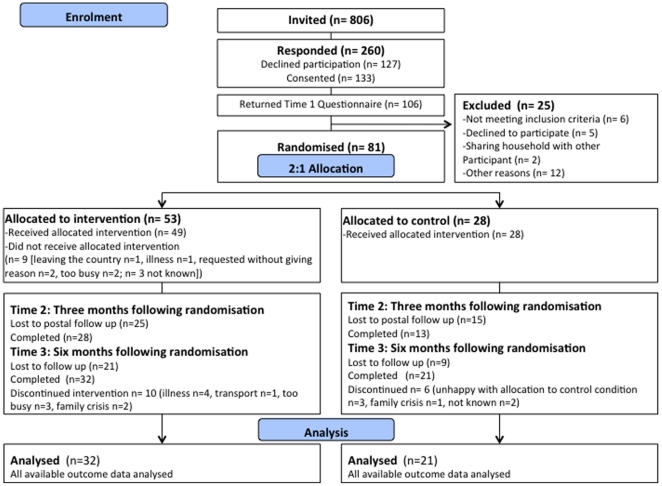
CONSORT Study Flowchart.

The primary outcome for this study was acceptability and feasibility of procedures for recruitment, allocation, measurement, retention and for the intervention procedures. Recruitment rates were measured as rate of invited participants consenting/eligible and are reported in a CONSORT participant flow chart (Figure 1). Acceptability of allocation procedures was assessed examining reasons for dropout in discontinuing participants and comparing attrition rates between both conditions. Suitability of measurement procedures was evaluated based on completion rates and, where applicable, psychometric information about reliability. Attrition rates were established as discontinuation of intervention and loss to follow-up measurement for both conditions. We also recorded completion of intervention materials as an indicator of participation. We attempted fidelity assessment through audio-recordings of intervention sessions. However, these recordings are not available as the facilitator perceived manual recordings as obtrusive to the proceedings and discontinued the recording.

### Trial outcomes

In addition to acceptability and feasibility, we measured the following anthropometric and performance outcomes relevant for a full trial: weight in kg, height in cm, waist circumference, waist-to-hip ratio, %body fat (Omron BF306 handhold body fat monitor), blood pressure, resting heart rate and the 6-minute walk test [39] to assess fitness were all objectively measured at Time 1 and Time 3 by an experienced research nurse using standard operating procedures in the Clinical Research Facility, Aberdeen of University. Dyspnea and fatigue pre and post 6 minute walk test were measured on a standard 10 point Borg scale [40].

Questionnaires administered at Time 1, Time 2 and Time 3 included self-reported physical activity [41], walking in min/day and dietary intake through a validated food frequency questionnaire [42]. Process measures included a Theory of Planned Behaviour (TPB) questionnaire [43], Action Planning and Coping Planning Scales [44], and the Action Control Scale [45] for physical activity and adherence to a healthy weight loss diet as well as the ENRICHd Social Support Scale [46] and a shortened version of the Illness Perception Questionnaire–R (IPQ-R PS) [47], [48].

### Sample size

The study aimed to recruit 90 participants as this gives precision of at least 5 percentage points on any estimated acceptability/feasibility proportion (i.e. the 95% confidence interval limits around the estimated proportion of, for instance, willingness to be randomised ±10%) [49].

### Randomisation

Individual computer generated randomisation to intervention or control condition in a 2∶1 ratio, using a secure centralised web-based randomisation system provided by the Health Services Research Unit (HSRU), connected to a database with appropriate user level security. Randomisation was performed by FFS. Participants were informed about condition allocation by KR without involvement of the outcome assessor.

### Blinding

Participants and intervention facilitator were aware of condition allocations. The outcome assessor was blinded to allocation. Returning participants at Time 3 assessment were asked not to disclose allocation. The assessor was instructed to remind participants not to disclose study group allocation.

### Statistical analyses

We describe the response rates to invitation, recruitment and adherence using proportions for the whole pilot group, and by intervention group at each time point. We investigated the completion patterns of materials. Reliability of instruments was assessed using Cronbach's alpha over the whole pilot group irrespective of treatment allocation. Logistic regression analysis was used to investigate if invitees who provided consent to participate differed from those who did not in terms of demographics, clinical variables, general practice or post code linked Scottish Index of Multiple Deprivation deciles (SIMD) [50]. We originally planned to use the intervention group data to explore any potential clustering due to the intervention being delivered to groups of participants and estimate an intra-cluster correlation to inform power calculations for any future RCTs. However, given the loss to follow-up it was not feasible to calculate the correlations. No clustering occurred in the control group. Effect size for the main outcome was explored using analysis of covariance.

## Results

Figure 1 shows the CONSORT flowchart for this study.

Eight hundred and six eligible individuals, aged between 19–89 years (mean = 57.3, SD = 15.2) were identified, and sent a study invitation. 260 (32.3%) invitees responded and 133 (51.2%) provided informed consent. The procedures did not allow checking of the percentage of invitees who received the invitation letter. Due to the initially high consent rates, it was decided not to send reminders to invitees as 133 consents were considered sufficient to reach the target of n = 90 participants randomised. A multiple logistic regression analysis examined if consent was related to gender, age, SIMD, or type and number of obesity related co-morbidities/risk factors (Table 1). SIMD emerged as a significant predictor (p = .007) for providing consent to take part in the study; for every increase in SIMD score (indicating lower deprivation levels), the odds of participation increased by 1.3 times.

**Table 1:** Predictors of consent to participate in the study (n = 806).

Predictor	p	OR (CI 95%)	β	SE β	Wald's *χ* ^2^	df
Gender (female)	.280	.804 (.541–1.194)	−.218	.202	1.168	1
Age	.072	.985 (.970–1.001)	−.015	.008	3.241	1
SIMD score	.007	1.259 (1.064–1.488)	.230	.086	7.234	1
GP Practice	.757	1.081 (.659–1.774)	.078	.252	.096	1
Type II diabetes	.449	1.348 (.623–2.916)	.298	.394	.574	1
Impaired glucose tolerance	.636	.806 (.329–1.972)	−.216	.457	.224	1
Hypertension	.387	1.323 (.701–2.494)	.280	.324	.747	1
Coronary heart disease	.602	1.246 (.546–2.843)	.220	.421	.273	1
Cerebrovascular disease	.070	2.862 (.919–8.916)	1.052	.580	3.290	1
Arthritis	.402	1.302 (.703–2.412)	.264	.315	.703	1
Lipid disorders	.531	1.303 (.569–2.986)	.265	.423	.392	1
Diseases of the airways	.600	1.175 (.643–2.146)	.161	.307	.274	1
No. of co-morbidities	.088	1.482 (.943–2.329)	.394	.231	2.913	1
Constant	.083	.019	−3.953	2.277	3.013	1

*Note*. The regression model did not differ significantly from the intercept only model (*χ* ^2^(1) = 20.614, p = .081). However, rather than to better predict the outcome, the focus was to examine associations between independent and dependent variables as well as the predictive abilities of independent variables on dependent variables.

Consenting participants were sent a baseline questionnaire, which was returned by 106 individuals who received invitations to attend baseline assessment. Twenty-five participants were excluded (due to not meeting inclusion criteria, change of mind, or inability to contact). The remaining 81 participants were randomised. These participants had Type 1 diabetes (n = 1; 1.2%), Type 2 diabetes (n = 17; 21%), IGT (n = 9; 11.1%), hypertension (n = 47; 58.0%), heart disease (n = 15; 18.5%), cerebrovascular disease (n = 2; 2.5%), rheumatoid/osteo-arthritis (n = 20; 24.7), hyperlipidaemia (n = 13; 16%) and COPD/Asthma (n = 22; 27.2%); n = 44 (54.3%) participants were regular statin users.

Table 2 shows the pre-randomisation baseline characteristics. The total sample was on average 56.6 years (SD = 11.4), 63% (n = 51) female, BMI of 36.73 kg/m^2^ (SD = 6.1) and were diagnosed with 2.4 (SD = 1.5) additional risk factors/comorbidities. Mean SIMD decile was 4.8 (SD = 3.1; range 1 [lowest level of social deprivation]-10[highest level of deprivation]).

**Table 2:** Means (standard deviation) of pre-randomisation baseline characteristics in control and intervention group.

Variable	Control (n = 28)	Intervention (n = 53)
Female	71.4%	58.5%
Age (years)	61.04 (7.73)	54.41 (12.40)
SIMD (deciles)	4.70 (3.09)	4.79 (3.17)
Participants from GP practice A	46.4%	49.1%
No of conditions/risk factors	2.79 (1.57)	2.11 (1.37)
**Body composition**		
Height (cm)	164.56 (9.57)	166.04 (8.17)
Weight (kg)	93.81 (15.60)	104.77 (22.41)
BMI (kg/m2)	34.59 (4.65)	37.86 (6.44)
%Body fat	42.09 (5.83)	42.40 (5.97)
Waist circumference (cm)	111.07 (9.82)	115.91 (14.89)
Hip circumference (cm)	121.58 (15.83)	120.04 (14.60)
Waist-to-hip ratio	.92 (1.06)	.97 (.09)
**Cardiovascular functions**		
Resting heart rate	77.61 (11.72)	76.91 (13.34)
Systolic blood pressure	143.00 (17.81)	146.89 (20.09)
Diastolic BP	79.36 (9.58)	85.47 (11.10)
**Fitness**		
6 Minute Walk Test (distance in m)	443.04 (93.48)	435.09 (108.18)
- Post 6MWT dyspnea	1.26 (1.14)	1.65 (1.25)
- Post 6MWT fatigue	.50 (1.04)	.81 (1.31)
**Behaviour**		
Protein intake (g/day)	107.02 (36.74)	123.82 (77.25)
Fat intake (g/day)	99.81 (45.14)	127.15 (100.46)
Carbohydrate (g/day)	290.6 (126.89)	365.60 (230.04)
Alcohol (g/day)	6.70 (10.26)	9.17 (11.16)
Kcal (day)	2459.32 (1002)	3072.41 (2028)
Physical activity (G-LTEI)	31.45 (27.21)	21.52 (24.44)
Walking (minutes/day)	65.77 (93.55)	67.96 (86.93)

*Note*. GP practice A indicates proportion of sample recruited from the GP practice located in the more deprived area. Differences between groups were not tested for significance as allocation was randomised.

### Baseline characteristics of control and intervention groups

Participants allocated to the control condition were more likely to be female (71.4% vs. 58.5%), older (M_control_ = 61.0 years vs. M_intervention_ = 54.4), shorter (M_control_ = 164.6 vs. M_intervention_ = 166.0), lighter (M_control_ = 93.8 kg vs. M_intervention_ = 104.8 kg), and with a smaller waist circumference (M_control_ = 111.1 cm vs. M_intervention_ = 115.9 cm). Since group allocation was randomised, no p-tests for differences were conducted as the null-hypothesis of p-tests stating that both groups are taken from the same population, is already given. Due to an oversight, one participant with a BMI of 25.7 was included in the study and randomised to the control group. This participant did not provide follow-up data. Four participants had a BMI>50 (50.2; 51.0; 56.6; 61.2) and were all randomised to the intervention condition. Intervention participants reported higher fat, alcohol and calorie intake, and slightly lower levels of baseline physical activity compared to control participants. Since the randomisation procedure was robust and not compromised, these differences are most likely due to chance and a result of the small sample size, particularly in the control condition as a result of the unbalanced randomisation (2∶1).

### Intervention procedures and participation

Of 53 participants randomised to the intervention 49 were allocated to one of seven groups ranging from 4–9 participants. Reasons for initial dropout were ‘too busy’ (n = 2), ‘leaving the country’ (n = 1) or ‘no reason’ (n = 1). Of 49 allocated participants 40 attended at least 1 session. Reasons for not attending a single session were ‘not known’ (n = 3), ‘illness’ (n = 2), ‘too busy’, ‘family crisis’, ‘no reason’, and ‘transport’ (all n = 1). Of the 40 attending participants a further 6 did not complete the intervention due to ‘illness’ (n = 3), ‘too busy’ (n = 2) and ‘family crisis’ (n = 1). Total loss to follow-up in the intervention group was n = 21 (39.6%), attrition amongst those who attended at least one session was 15%.

In the control intervention, three participants (10.7%) discontinued study participation explicitly referring to disappointment about their allocation. During initial telephone contacts, anecdotal evidence suggested that participants intended to drop out if allocated to the control condition (labelled *written information* intervention). It was not possible to obtain a reliable measure for participants reading the leaflets sent to them.

### Session attendance

Of n = 49 allocated participants the average attendance was 3.8 (SD = 2.4), equalling an average attendance of 64.2% of allocated session slots. Participants completing the intervention attended an average of 5.2 (SD = 1.2) sessions. Twenty-one participants attended all 6 scheduled sessions (43%). The majority of participants attended session 1 (n = 40, 81%), with numbers reducing to 34 (69%), 32 (65%), 29 (59%), 29 (59%) and 23 (47%) for sessions 2 to 6 respectively. Due to facilitator illness, session 6 had to be rescheduled once with only half the participants (n = 3) returning for the final session.

The original intention for this feasibility trial was to form groups of 10 but due to limited staff capacity, initial assessment and consequently randomisation was slow. In order to avoid longer waiting times, smaller groups were formed. Since not all participants attended, some groups were too small (e.g. n = 4). Attendance varied by intervention group. The two groups with the lowest attendance had the lowest number of participants scheduled (6 and 4 participants). Group 6, experienced parking problems with the parking attendant refusing to accept valid parking permits, leading to 3 participants not being able to attend the sessions and subsequently dropping out. Scotland experienced a severe winter with considerable snowfall and disruption to traffic and public transport during the main period of intervention delivery. Anecdotal evidence suggests that this made attendance at sessions difficult, and may have contributed to attrition.

### Material completion

Of 49 allocated participants the average number of intervention booklets returned was 2.9 (SD = 2.1) out of 5, an average booklet return of 58%. Participants returned >50% of booklets for all sessions except 5. Participants attending a session returned 77.6% (SD = 12.8) of received booklets. Lowest returns were noted for sessions 1 (67%) and 5 (61%). For session 1, the majority of unreturned booklets (n = 5, 38%) was in the first group, with the facilitator not explicitly mentioning the need to return booklets. The increase for booklets participants received in session 5 could have resulted from the longer interval between sessions 5 and 6.

### Process measures, psychometric properties and completion of questionnaires

At baseline, completion of the questionnaire was a prerequisite for randomisation. Postal questionnaires containing self-reports of behaviour and psychological process measures were returned at Time 2 by n = 42 (51.9%) and at Time 3 n = 48 (59.3%) participants indicating that a postal questionnaire might be a limited method to obtain process measures from all participants. Not all participants completed the self-report of behaviour. The Godin Leisure Time Physical Activity Index [41] was available at Time 2 from n = 30 and Time 3 from n = 37 participants. Data for the Food Frequency Questionnaire [42] were available for n = 41 at Time 2 and n = 26 at Time 3. There was anecdotal evidence from participants who received telephone calls reminding them about returning the questionnaire that the food frequency questionnaire in particular was perceived as unduly lengthy and complicated.

Table 3 shows that participants at baseline attributed their obesity more often to energy balance behaviours than to emotional/psychological, genetic or external causes. Baseline IPQ-R PS measures showed low levels of coherence (understanding the nature of the individual weight problem) and beliefs about personal control over weight. Higher perceptions of consequences, emotional response and treatment beliefs indicate that external treatments (rather than personal control) are seen as more effective for decreasing body weight. TPB variables showed positive cognitions about physical activity and healthy eating behaviours. Notably, perceived control and intentions for dietary changes are higher than for physical activity. Baseline data show low levels of self-regulation (action planning, coping planning and action control). These constructs were key targets of the intervention. Over the course of the study, these measures showed only slight changes and did not suggest any major group differences.

**Table 3:** Baseline self-reported measures of behaviour and psychological variables, available data at Time 2 and 3 for all participants and Cronbach's alphas.

	Control			Intervention		
Variable	Time 1	ΔT1/T2	ΔT1/T3	Time 1	ΔT1/T2	ΔT1/T3
IPQ-R PS (1–5)						
Causes (frequency)						
Phys. activity/diet	1.54 (.92)	−.15 (1.46)	−.16 (.62)	1.65 (.99)	.30 (.96)	.02 (1.10)
Emotional/motivational	.43 (.88)	−.15 (.99)	−.18 (.84)	.42 (.70)	.02 (.55)	.17 (.88)
genetic	.18 (.39)	−.15 (.38)	−.16 (.37)	.13 (.39)	−.14 (.36)	−.07 (.37)
external	.46 (.64)	.31 (1.03)	.45 (.93)	.53 (.64)	.14 (.89)	−.02 (.63)
Timeline						
Acute-chronic (α = .81)	3.20 (.78)	.23 (.69)	.40 (.79)	3.23 (.97)	−.29 (.97)	−.02 (.81)
cyclical (α = .84)	3.33 (1.01)	−.10 (.74)	−.14 (.91)	3.24 (.89)	.08 (.82)	−.04 (.82)
Treatment control (α = .79)	3.27 (.75)	−.60 (.88)	−.40 (.85)	3.16 (.78)	−.33 (.89)	−.67 (.90)
Personal control (α = .64)	1.96 (.43)	.24 (.65)	.32 (.51)	2.13 (.72)	−.17 (.48)	−.05 (.52)
Consequences (α = .79)	3.28 (.80)	−.04 (.54)	.22 (.70)	3.38 (.88)	.14 (.78)	.06 (.53)
Coherence (α = .85)	2.90 (.91)	.28 (.48)	.27 (.73)	2.64 (.83)	.29 (.82)	.46 (.91)
Emotional Response (α = .87)	3.46 (1.02)	−.10 (.66)	.19 (1.05)	3.48 (.97)	.31 (.70)	.44 (.80)
Motivation towards physical activity (−3–3)						
Attitude (α = .86)	1.97 (.91)	−.43 (.84)	.24 (1.11)	1.77 (1.0)	−.21 (.87)	.12 (1.10)
Subjective Norm	2.04 (1.02)	.10 (.88)	.88 (2.00)	1.78 (1.40)	.50 (2.02)	.77 (2.03)
PBC (α = .83)	1.27 (1.36)	.19 (1.28)	.27 (1.83)	1.03 (1.46)	−.09 (1.44)	−.12 (1.33)
Intention	1.55 (1.26)	−.27 (1.90)	.13 (2.06)	1.71 (1.23)	.15 (1.43)	.26 (1.35)
Action planning (α = .97)	−.88 (1.96)	−1.40 (2.11)	−.67 (2.52)	−1.11 (1.93)	−.59 (2.39)	−.25 (2.63)
Coping Planning (α = .97)	−1.25 (1.74)	−.52 (1.07)	−.33 (1.07)	−1.43 (1.69)	−.79 (2.06)	−.76 (2.24)
Self-regulation (α = .93)	−.86 (1.72)	−.88 (1.04)	−.41 (1.84)	−1.30 (1.62)	−1.62 (1.51)	−1.00 (1.91)
Motivation towards a healthy weight loss diet (−3–3)						
Attitude (α = .85)	1.90 (1.17)	−.09 (1.09)	.08 (1.31)	1.39 (1.40)	−.58 (1.16)	0.19 (1.26)
Subjective Norm	2.28 (.79)	.25 (.62)	1.12 (2.09)	1.98 (1.48)	−.20 (1.78)	.33 (1.71)
PBC (α = .90)	1.74 (1.33)	1.50 (.90)	.88 (1.53)	1.39 (1.34)	1.32 (.99)	.23 (1.09)
Intention	2.00 (.96)	.23 (1.09)	.50 (1.04)	1.80 (1.13)	−.35 (1.09)	.11 (1.50)
Action planning (α = .93)	.22 (2.00)	−1.06 (1.93)	−.57 (2.19)	−.62 (1.90)	−1.54 (1.70)	−.59 (2.09)
Coping planning (α = .98)	−.67 (1.88)	−.76 (1.67)	−.27 (2.19)	−1.25 (1.79)	−1.57 (1.70)	−1.20 (1.02)
Self-regulation (α = .91)	−.38 (1.58)	−.91 (1.66)	−.11 (1.64)	−.87 (1.44)	−1.47 (1.48)	−.90 (1.72)
Social Support (α = .94)	3.78 (1.25)	−.06 (.88)	.07 (.64)	3.46 (1.18)	−.05 (.96)	.15 (.87)

Note. Intention and subjective norm measures were based on single items; The psychometrically shortened version of the IPQ-R was used.

### Loss to follow-up and changes in outcomes from baseline to 6 months

Table 4 shows the outcome measures at baseline, 3 and 6 months. Due to limited sample size in the control condition we abstained from imputing missing data.

**Table 4:** Changes in outcome measures from baseline (Time 1) to six month (Time 3) in control and intervention group with standard deviation (SD), intention-to-treat for all participants completing the study and ANCOVA statistics controlled for sex, height and baseline outcome measure.

Variable	Control	Intervention	Adj. mean differences ANCOVA statistics
**Body composition**			
Weight (kg)	−1.18 (4.21)	−2.58 (3.91)	−1.36 (CI95% −3.75 | 1.03)*F*(1,52) = 1.311, *p* = .258
%Body fat	.10 (1.52)	−.72 (1.79)	−.88 (CI95% −1.88 | .11)*F*(1,48) = 3.200; *p* = .081
Waist circumference (cm)	−1.64 (3.86)	−4.24 (4.12)	−2.43 (CI95% −4.82 | −.04)*F*(1,52) = 4.171, *p* = .047
**Cardiovascular functions**			
Resting heart rate	−2.90 (13.72)	−5.09 (9.04)	−2.30 (CI95% −8.67 | 4.08)*F*(1,52) = .525; *p* = .472
Systolic blood pressure	−8.10 (14.86)	−10.47 (13.56)	−.166 (CI95% −8.96 | 5.63)*F*(1,52) = .210; *p* = .648
Diastolic blood pressure	−3.00 (7.91)	−4.16 (8.23)	.31 (CI95% −4.11 | 4.73)*F*(1,52) = .019; *p* = .890
**Fitness**			
6 Minute Walk Test (m)	15.68 (41.65)	15.25 (39.92)	−.25 (CI95% −27.14 | 22.14)*F*(1,49) = .042; *p* = .839
- Post 6MWT dyspnea	−.40 (.93)	−.68 (.90)	−.26 (CI95% −.69 | .16)*F*(1,49) = 1.537, *p* = .221
- Post 6MWT fatigue	.045 (1.32)	−.26 (1.09)	−.18 (CI95% −.77 | .40)*F*(1;49) = .401, *p* = .530
**Behaviour**			
Protein intake (g)	−20.51 (28.04)	−21.48 (61.38)	
Fat intake (g)	−17.97 (33.28)	−53.56 (103.68)	
Carbohydrate (g)	−29.67 (56.82)	−128.81 (274.10)	
Alcohol (g)	−2.38 (16.51)	−.59 (3.51)	
Kcal	−373.0 (510.3)	−1051.2 (2175.8)	
Physical activity (G-LTEI)	9.55 (19.01)	16.92 (14.85)	

Note. Only n = 9 participants in the control and n = 16 participants in the intervention condition completed the Food Frequency Questionnaire and n = 11 controls and n = 20 intervention participants completed the Godin physical activity index. Due to these limited sample sizes, no *F* statistics for these measures are reported.

Six months after baseline assessment, 21 (75%) control participants and 32 (60.4%) intervention participants completed Time 3 measurements. The difference in attrition between both groups was not significant (*χ* ^2^(1,80) = 1.732; *p* = .142). A logistic regression was conducted to test if Time 3 completion was associated with age, sex, SIMD, BMI, and distance walked in 6 minutes at baseline. None of these variables was predictive of attrition from the study. Since the sample size did not allow multiple imputation of missing values, an intention-to-treat analysis based on available Time 3 measures was conducted.

Table 4 reports the changes in outcome measures for control and intervention groups and ANCOVAs comparing Time 3 outcome measures between groups, controlled for sex, BMI and the respective outcome measure at baseline. Participants in the intervention group lost 4.24 cm (4.12) of waist circumference (controls: M_change_ = 1.64 cm; SD = 3.86; *F*(1,52) = 4.171, *p* = .047), 2.58 kg (3.91) of body weight (controls: M_change_ = 1.28; SD = 4.21; *F*(1,52) = 1.311, *p* = .258), reduced their body fat by 0.72% (1.72) (controls: M_change_ = −0.10; SD = 1.52; *F*(1;52) = 3.200; *p* = .081), resting heart rate by 5.09 (9.04) beat per minute (controls: M_change_ = 2.90; SD = 13.72; *F*(1,52) = .525; *p* = .472), reduced systolic blood pressure by 10.47 mmHg (13.56) (controls: M_change_ = 8.10; SD = 14.86; *F*(1,52) = .210; *p* = .648) and reduced diastolic BP by 4.16 mmHg (8.23) (controls: M_change_ = 3.00; SD = 7.91; *F*(1,52) = .019; *p* = .890). Both groups slightly improved 6 minute walk test performance and showed minimal changes in post walk test dyspnea and fatigue.

Participants in the intervention group reported increased physical activity (increase of 16.92 points (14.85) on the Godin Leisure Time Physical Activity Index vs. 9.55 (19.01) for controls). Similar pattern was found for dietary behaviours. Participants in the intervention group reported a reduction of energy intake by 1051.19 kcal (2175.8) compared to 373.00 (510.3) for controls mirrored by similar differences for fat and carbohydrate intake, while protein and alcohol intake showed fewer change. However, since fewer participants completed the food frequency questionnaire (see Table 3) these trends must be interpreted with caution.

## Discussion

This study tested the acceptability and feasibility of the recruitment, measurement, allocation and intervention procedures for an RCT of a behaviour change intervention designed for obese adults with additional obesity related risk factors and conditions.

### Feasibility and acceptability of recruitment procedures

Identification of eligible individuals from primary care General Medical Practice (GP) lists and sending study invitations on practice headed letters through SPCRN staff was feasible and acceptable to GP practice staff. Two GP practices in areas at different ends of the socio-economic spectrum were purposively chosen for recruitment to provide insight into the potential effects of relative affluence or deprivation on recruitment and participation. A total of 16.5% of invitees provided consent. As the researchers did not have personal information about invitees it was not possible to confirm how many invitees actually received the invitation letter and materials and to what degree the primary care centre patient list data relevant to the eligibility criteria were accurate. Since the initial consent rate was high, no reminder letters were sent. Out of the n = 133 consenting individuals, only n = 106 returned the baseline questionnaire which was a prerequisite for entering the study. This reduction in numbers suggests that the baseline questionnaire was not sufficiently acceptable to participants. Of 106 participants who returned the baseline questionnaire, an unexpectedly high number could not be randomised; due to inaccurate/outdated GP medical records, participants withdrawing or inability to contact patients. Therefore 81 (10.0% of those initially invited), rather than 90 individuals were randomised. These figures can inform estimates of the number of invitations required for a definitive trial.

We also found that people from more deprived neighbourhoods were slightly less likely to consent to taking part in this research. This small effect replicates prior research [51]. More research is needed to understand the mechanisms between this common effect and more extensive user involvement in the development of recruitment procedures might help further decreasing social inequalities in participation in randomised trials [52]. Overall, these findings suggest a) sending reminders to invitees b) decreasing the measurement burden and c) involving users in the redraft of participant information procedures to reduce bias in socio-economic status for a definite RCT.

### Feasibility, acceptability and reliability of outcome measures

All main outcome measures taken in a face-to-face setting in the Clinical Research Facility were found to be acceptable and feasible to participants and staff conducting the measurement as indicated by completion rates. The study found clear limitations in taking measures by postal questionnaires. The response rates for the questionnaires at Time 2 and Time 3 were lower than for the anthropometric and performance assessment conducted face to face. While there were relatively few missing values for the psychological process variables measured in the same questionnaire, outcome data for physical activity and diet at Time 3 were available for >50% of the initial sample. The Food Frequency Questionnaire was long and required detailed reporting for a day's worth of dietary intake. The high levels of missing values for the Godin Leisure Time Physical Activity Index were more unexpected as this measure has frequently been used in similar populations before. Overall, the questionnaire might have been too long and in parts too complicated for participants. For a future trial, it would be advisable to collect the behavioural outcome data face-to-face and to reduce the measurement burden through process measures.

The psychological process measures showed better completion rates and reliability. These measures are acceptable and feasible. On the IPQ-R PS, participants reported low levels of coherence (e.g., understanding their obesity problem) and low perceptions of personal control over their obesity. The intervention addresses the latter, by targeting people's sense of control over their food intake and physical activity, but not the former, since education and provision of information where not found to be effective techniques in the systematic review informing this intervention [27]. However, in the light of this evidence, it might be sensible to add an advice session to the intervention to ensure the necessary and accurate knowledge and feeling of control over their weight problem. Motivation to engage in physical activity and dietary changes was generally high, and slightly more favourable for diet than for physical activity. Generally, participants reported low baseline levels of self-regulation and planning. These factors were explicitly targeted as part of the intervention and were addressed extensively during training of the group facilitator.

### Feasibility and acceptability of trial and intervention procedures

Important lessons about the trial procedures were learned. Overall loss to follow-up of 34.6% is unacceptably high to proceed to a full RCT. In addition to the excessive measurement burden introduced through the food frequency questionnaire, specific issues in both conditions were identified as contributing to attrition. Some participants allocated to the control condition dropped out of the trial because they were disappointed not to be allocated to the face-to-face intervention. Consequently, for a full trial, a more attractive control intervention or a waiting list design should be considered.

For the behaviour change intervention group, patterns of retention were more complex and two main challenges emerged. Firstly, out of 53 individuals allocated to the intervention only 49 were available to be scheduled for an intervention group meeting. The relative small scale of this pilot study did not allow offering more alternative time slots for group sessions and in a future definitive trial it would be critical to either clarify the times of group sessions at the invitation stage, or to match group sessions to the availability of participants. Secondly, only 40 participants attended at least one meeting. The fact that nearly 20% of those who agreed to attend a session at a particular time never attended a single meeting indicates a substantial problem frequently observed in health service delivery and clinical trials that individuals do not attend scheduled appointments. Recently, several studies have shown that inexpensive theory based intervention based on simple planning interventions, letters or leaflets may be effective in improving attendance [53], [54] and for the present line of research it might be sensible to explicitly add an intervention focussing on attendance to the first meeting in order to improve participation. For those participants who attended at least one meeting, the intervention was found to be highly acceptable as indicated by intervention retention and material completion. Smaller groups had higher attrition, and the group facilitator reported better group dynamics in larger groups. The fact that it was not found feasible in this small scale study to form larger groups due to the relatively small number of participants, might have further increased attrition. In the future conducting a larger scale trial with more participants and sufficient staff time would enable the composition of larger groups. In addition, particular events (facilitator illness; parking problems, major snow disruptions, small groups, pandemic swine flu) had an impact on the study. Where no particular events impacted on the proceedings, the acceptability and feasibility of the intervention procedures were high, replicating the prior pilot study in secondary care. Material completion rates were high, indicating intervention engagement as intended.

This study has shown that there are sufficient numbers of potential participants living in the geographic area served by two primary care medical practices meeting the eligibility criteria to conduct a definitive RCT of the intervention delivered in this study. The main implications for a full trial are twofold. First, it would be advisable to hold intervention sessions in a community rather than hospital setting, reducing travel and parking problems. Second, it is critical to ensure availability of sufficient staff for a future trial, an issue which proved problematic in this feasibility study. Limited capacity in baseline assessment resulted in slow participant intake and smaller than expected intervention group sizes. Limited capacity in staff delivering the intervention resulted in several sessions being cancelled due to illness of the facilitator. Both factors were associated with attrition. In this regard, managing a larger trial using similar method might indeed prove easier to manage.

### Changes in outcomes

Despite robust randomisation procedures we found considerable imbalances between intervention and control groups. In particular, the intervention group included participants who were more obese and more men, reflecting the small scale nature of this study. In a definitive trial a minimisation procedure with BMI included in the minimisation variables should be used to allocate participants to groups.

For all objectively recorded outcomes of body composition and cardiovascular function, positive changes favouring the intervention were observed. Intervention participants lost on average 2.58 kg (3.91) of weight at 6 months and 4.24 cm (4.12) waist circumference. Only changes in 6 Minute Walk Test performances did not show a trend favouring the intervention group, although trends favouring the intervention group for pre-post changes in dyspnoea and fatigue were found. The study was not powered to detect group differences, therefore trends should not be overemphasised even though the intervention group lost significantly more waist circumference than controls. The weight change is slightly lower compared to the mean weight difference of our systematic review which includes considerably more intensive interventions. Further trends for increased physical activity favouring the intervention and decreased calorie intake in both groups were found based on very small sample sizes.

### Conclusion

We found that a protocol for a RCT of a relatively brief six session behaviour change intervention for obese adults with additional risk factors based on a systematic and evidence based development process needs further modification to be viable for a definitive effectiveness trial. Based on the data from this feasibility trial, changes to recruitment, measurement and retention procedures are proposed.

The intervention procedures were shown to be acceptable to participants attending at least one session and trends in changes in outcome variables favoured the intervention group but the trial that was not powered to detect group differences. Methods to further improve feasibility and acceptability of the trial procedures were identified and will be implemented in a subsequent definitive RCT.

## Supporting Information

Checklist S1 CONSORT Checklist.
(DOC)

Protocol S1 Protocol Main Document.
(DOC)

Protocol S2 Protocol appendix 1: General Practice Invitation Letter; version 1.
(DOC)

Protocol S3 Protocol appendix 2: General Practice Fact Sheet; version 2.
(DOC)

Protocol S4 Protocol appendix 3: Patient Invitation from GP; version 2.
(DOC)

Protocol S5 Protocol appendix 4: Participant Information; version 4.
(DOC)

Protocol S6 Protocol appendix 5: Reply Slip; version 2; 21 07 2009.
(DOC)

Protocol S7 Protocol appendix 6: Consent Form; version 3.
(DOC)

Protocol S8 Protocol appendix 7: Standard Operating Procedures for Blood pressure and Heart Rate Measurement, Clinical Research Facility, University of Aberdeen.
(DOC)

Protocol S9 Protocol appendix 8: 6 Minute Walk Test Standard Operating Procedures.
(DOC)

Intervention Manual S1 Intervention Manual Session 1.
(PDF)

Intervention Manual S2 Intervention Manual Session 2.
(PDF)

Intervention Manual S3 Intervention Manual Session 3.
(PDF)

Intervention Manual S4 Intervention Manual Session 4.
(PDF)

Intervention Manual S5 Intervention Manual Session 5.
(PDF)

Intervention Manual S6 Intervention Manual Session 6.
(PDF)
