# Supplementary material for: Randomised Controlled Feasibility Trial of an Evidence-Informed Behavioural Intervention for Obese Adults with Additional Risk Factors
Source: PLoS One. 2011 Aug 29;6(8):e23040. doi: 10.1371/journal.pone.0023040 (PMC3163575; doi:10.1371/journal.pone.0023040)
Supplement: Protocol S1 — Protocol Main Document. (DOC) [file pone.0023040.s002.doc]

**Protocol: Feasibility Study for a New Behavioural Intervention for Weight Loss**

**Purpose**: The purpose of the proposed study is to test the feasibility and acceptability of the intervention, measurement and trial procedures for a Randomised Controlled Trial of a newly developed behaviour change intervention in a sample of obese adults with additional risk factors for disease recruited from GP practice lists.

**Recruitment of collaborating GP practices**:

The Scottish Primary Care Research Network (SPCRN) will support us in finding 1-3 (depending on list size) GP practices in Grampian (preferably Aberdeen) taking part in the General Medical Services Quality & Outcomes Framework (QOF) to identify potential participants. Amanda Cardy from SPCRN is our collaborator and has kindly advised us in putting together this application.

*Documents: GP INVITE.doc and GP FACT SHEET.doc*

Once a GP has agreed to collaborate, SPCRN staff would visit the practice, select eligible patients and leave the list for the GP to check in case there is anyone it would be inappropriate to invite for any reason.

Included participants are adult patients of the lists of GP practices in Grampian

who have a BMI ≥30 and co-morbidities such as type 2 diabetes, impaired glucose tolerance or hypertension.

Exclusion criteria: Insufficient knowledge of the English language to take part in group interventions and use written materials, conditions preventing participants from engagement in mild-moderate physical activities such as walking.

In collaboration with SPCRN we will seek to access 'support for science' funds (as the study is CSO funded to reimburse the practices and we anticipate that about £130-£180 would cover the costs for each practice.

**Recruitment of patients**:

After the GP has approved the list of potential participants, SPCRN will return to the practice to run mailmerge and send invitations out (packs including informed consent sheets being provided by researchers). After two weeks, reminders will be send to patients who have not responded to the initial invitation.

As we plan to include 90 participants in the study, we anticipate based in SPCRN's experiences that at least 300 invitation letter would need to be send out.

*Documents: Invitation letter from GP ; PATIENT INFORMATION SHEET.doc; Response slip.doc; CONSENT FORM.doc*

Participants can either complete, sign and return the consent sheets using provided freepost return envelopes, or they can contact a member of the study team via phone/email to ask for further information ans ask questions about the research. Members of the research team who are GCP (Good Clinical Practice) trained, will then make telephone contact to schedule the initial assessment visit.

**Design**: Randomised pilot study with measurements pre intervention, three and six months post randomisation. Participants will be randomised to either standard care + written information (leaflets) or standard care + the intervention. An experienced researcher will deliver the intervention in a group setting in five weekly 1.5 hour sessions, and one follow-up session 3 weeks after the fifth session. This pilot investigation will test procedures, materials and measures of the intervention in order to further develop and adapt the current intervention with a view to a full randomised controlled trial. The design has been chosen in line with MRC guidelines on complex intervention development and following the systematic development and pre-piloting of the intervention.

**Measures**: The primary measures are acceptability and feasibility of intervention procedures. Furthermore, changes in weight, waist-hip ratio, body composition, 6Minute Walking test performance, psychological determinants of behaviour, diet, and physical activity will be assessed.

Table 1: Measures used and time of measurement

| Questionnaire | Baseline | 3 months follow-up | 6 months follow-up |
| --- | --- | --- | --- |
| *Personal Background Information* | X |  |  |
| *Behaviour* |  |  |  |
| Godin Leisure Time Physical Activity Questionnaire ^1^ | X | X | X |
| Adult Food Frequency Questionnaire ^2^ | X | X | X |
| *Cognitions* |  |  |  |
| Theory of Planned Behaviour ^3^ | X | X | X |
| Action Planning and Coping Planning Scales ^4^ | X | X | X |
| Action Control Scale ^5^ | X | X | X |
| ENRICHd Social Support Instrument ^6^ | X | X | X |
| Illness Perception Questionnaire –R (Short form) ^7^ | X | X | X |
| *Proportion of body fat* (Body Fat Monitor) using an Omron BF306 handhold body fat monitor which measures the body’s resistance to a high frequency pulse. | X |  | X |
| Resting heart rate and blood pressure (SOP in appendix) |  |  |  |
| 6 minute Walking test (Protocol in appendix) | X |  | X |
| 1. Godin G, Shephard RJ. A simple method to assess exercise behavior in the community. *Can J Appl Sport Sci*. 1985;10( 3):141-146.  2. Jia X, Aucott L, Milne AC, Craig LCA, McNeill G. Validation of a food frequency questionnaire in later life. *J Nutr Health and Aging*. in press.  3. Ajzen I. Constructing a TpB Questionnaire: Conceptual and Methodological Considerations. http://people.umass.edu/aizen/pdf/tpb.measurement.pdf. Updated 2006. Accessed April 2008, 2006.  4. Sniehotta FF, Schwarzer R, Scholz U, Schuz B. Action planning and coping planning for long-term lifestyle change: Theory and assessment. *Eur J Soc Psychol*. 2005;35( 4):565-576.  5. Sniehotta FF, Scholz U, Schwarzer R. Bridging the intention-behaviour gap: Planning, self-efficacy, and action control in the adoption and maintenance of physical exercise. *Psychol Health*. 2005;20( 2):143-160.  6. Vaglio, J., Conard, M., Poston, W.S., O'Keefe, J., Haddock, C.K., House, J. & Spertus, J. A. (2004). Testing the performance of the ENRICHD Social Support Instrument in cardiac patients. Health Qual Life Outcomes, 2: 24.  7. Moss-Morris, R., Weinman, J., Petrie, K. J., Horne, R., Cameron, L. D., & Buick, L. (2002). The revised illness perception questionnaire (IPQ-R). Psychology and Health, 17, 1–16. | | | |

**Intervention**

In intervention is based on a detailed manual.

*Document: Manual complete 19 01 09a-f.doc*

Scientific foundation of the intervention: The proposed intervention is based on a systematic review of 44 relevant randomised controlled trials. This systematic review identified successful theory−linked behaviour change techniques leading to beneficial weight loss outcome. These behaviour change techniques centre on the theoretical framework of self−regulation theory and include forming detailed action plans, barrier identification, self−monitoring, planning self−reward, planning social support, and relapse prevention.

Intervention description:

The intervention focuses on changing activity and dietary behaviours, and in turn weight and waist/hip ratio, in a sample of obese participants with additional risk factors. An experienced nurse will deliver six group sessions (five weekly sessions in the beginning of the intervention and a refresher session 3 weeks after session 5). The nurse will be trained and instructed in delivering the intervention in accordance with a detailed manual consisting of behaviour change techniques that have been identified as successful in terms of weight loss in our systematic review. Groups will include up to ten participants. The introduction of behaviour change techniques will follow a logical pattern with the introduction of action planning and self−monitoring in the first few sessions, followed by the introduction of subsequent techniques on a weekly basis concluding with relapse prevention towards the end of the intervention.

In addition, participants in the intervention group will receive brief encouraging letters, offering to send additional study materials (e.g. self-monitoring or goal setting sheets) and the leaflets 'So you want to lose weight... for good - A guide to losing weight for men and women' and 'Get Active' issued by the British Heart Foundation.

Intervention sessions will be recorded and anonymously transcribed. In the transcription, reference will only be made to facilitator and participants. The aim of the recording is to test the fidelity of the intervention delivery, i.e., if the intervention has been delivered as specified in the manual.

Control group (standard care plus written information from the British Heart Foundation)

Participants in the control group will receive the leaflets 'So you want to lose weight... for good - A guide to losing weight for men and women' and 'Get Active' issued by the British Heart Foundation.

Hypotheses: We hypothesise that the recruitment, intervention, measurement and trial procedures will be feasible and acceptable, thus allowing us to proceed with a full randomised trial.

Anticipated risks: There are no anticipated risks associated with participating in the current study. The cost to participants will be the time spent in group sessions and completing questionnaires. We believe that this topic will be of interest to the participants and that they may appreciate the opportunity to participate in an evidence based behaviour change research.

Timeline: The current project is intended to last 1 year. The initial preparation stage is assumed to take 3 months and data collection; analysis and write−up will take a maximum of 9 months.
