# Supplementary material for: Randomised Controlled Feasibility Trial of an Evidence-Informed Behavioural Intervention for Obese Adults with Additional Risk Factors
Source: PLoS One. 2011 Aug 29;6(8):e23040. doi: 10.1371/journal.pone.0023040 (PMC3163575; doi:10.1371/journal.pone.0023040)
Supplement: Protocol S2 — Protocol appendix 1: General Practice Invitation Letter; version 1. (DOC) [file pone.0023040.s003.doc]

Dear xxxxx

**The Aberdeen Behaviour Change (ABC) Study:**

**A pilot trial of a weight loss programme**

We are writing to ask if the practice would be willing to participate in a small exploratory study testing a new behaviour change intervention for obese adults with additional risk factors. It is being conducted jointly by the University of Aberdeen and Robert Gordon University, funded by the Scottish Government and approved by the North of Scotland Ethical Committee. The main thing that we are asking practices to do is help us to identify eligible patients. We do not envision that this project will generate additional work for the practice.

Obese patients that agree to take part, will be randomly assigned either to take part in **group intervention** sessions at Aberdeen Royal Infirmary, or to receive a **written information** package. All participants will be seen by one of our research nurses / health care assistants at the beginning and end of the study to measure their height, weight, waist and hip circumference. If required, we can make this information available to the practice.

What would be involved for your practice?

1. Generate a list of all patients aged over 18 years with a Body Mass Index (kg/m^2^) of 30 or higher and at least one additional risk factor (a full list is enclosed). If possible, and with your permission, a member of SPCRN staff would visit the practice to perform the search.
2. A GP to screen the list and remove any patients whom they felt it would be inappropriate to approach.
3. The SPCRN staff member (supplied by us) would send letters of invitation to potential patient participants, on behalf of the practice, seeking their consent to be involved in the study.

The practice will receive £xxx to reimburse the costs of participation.

If you are interested, please contact the SPCRN Co-ordinator, Amada Cardy, on 01224 55xxxx (email a.h.cardy@abdn.ac.uk) to arrange a practice visit. If you would like any further information about the study, Dr Falko Sniehotta (Tel: 01224 273216 [f.sniehotta@abdn.ac.uk](mailto:f.sniehotta@abdn.ac.uk) ) will be happy to discuss any specific issues.
