# Supplementary material for: Randomised Controlled Feasibility Trial of an Evidence-Informed Behavioural Intervention for Obese Adults with Additional Risk Factors
Source: PLoS One. 2011 Aug 29;6(8):e23040. doi: 10.1371/journal.pone.0023040 (PMC3163575; doi:10.1371/journal.pone.0023040)
Supplement: Protocol S3 — Protocol appendix 2: General Practice Fact Sheet; version 2. (DOC) [file pone.0023040.s004.doc]

**GP FACT SHEET**

**The Aberdeen Behaviour Change (ABC) Study: A pilot trial of a weight loss programme**

**Name of Study**

Feasibility Study for a randomised controlled trial of a behavioural intervention to reduce weight in obese adults with additional risk factors for chronic disease: **the Aberdeen Behaviour Change (ABC) Study**

**Funder**

Scottish Government (Chief Scientist Office)

**Aim**

To test if a novel group-delivered behaviour change intervention (six sessions of 90 minutes) and written leaflets is more effective in changing body weight, waist-hip ratio, calorie intake and physical activity than an active control group receiving written leaflets alone about physical activity and weight loss. This particular study is a pilot preceding a full randomised trial in which we seek to test the procedures for the full trial.

**Inclusion and exclusion criteria**

*Inclusion*: Age ≥ 18 years; Body Mass Index (kg/m^2^) ≥ 30 AND one of the following conditions: type 2 diabetes mellitus; hypertension, impaired glucose tolerance; ischaemic heart disease, hyperlipidaemia, osteoarthritis.

*Exclusion*: Conditions preventing from participation in mild activities (e.g., walking) and insufficient knowledge of the English language to benefit from a group interventions and/or written materials; deemed inappropriate to include by own GP.

**Participants, randomisation and Treatments**

N=90 participants will be randomised in a 2:1 ratio to either a **group intervention** condition or an **active** **control** condition.

*Group intervention*

Six sessions of 90 minutes delivered over two months by a trained research nurse. The intervention focuses on teaching behaviour change strategies and techniques which have been shown to be effective in a prior large scale systematic review of behavioural interventions for obese adults with additional risk factors for chronic disease. Participants will be provided with materials to assist behaviour change and a modern step counter (pedometer) to monitor their physical activity.

*Active Control*

Participants will receive ‘GET ACTIVE’ and ‘So you want to lose weight... for good - A guide to losing weight for men and women’ issued by the British Heart Foundation via post.

**Measures & Timing**

Height, weight, body fat, waist and hip circumference and fitness will be measured at baseline and three months after the completion of the intervention period. In addition, participants will be asked to complete a brief questionnaire at baseline, after the intervention period and three months later.

**Ethical approval**

North of Scotland Ethical Research Board (REC XXX)

**Researchers**

Dr Falko Sniehotta, Mr Stephan Dombrowski & Professor Marie Johnston (School of Psychology, University of Aberdeen); Dr Alison Avenell & Dr Craig Ramsay (Health Services Research Unit, University of Aberdeen); Dr Vera Araujo-Soares (Faculty of Health & Social Care; Robert Gordon University) and

Dr Peter Murchie (Centre of Academic Primary Care, University of Aberdeen)
