# Supplementary material for: Randomised Controlled Feasibility Trial of an Evidence-Informed Behavioural Intervention for Obese Adults with Additional Risk Factors
Source: PLoS One. 2011 Aug 29;6(8):e23040. doi: 10.1371/journal.pone.0023040 (PMC3163575; doi:10.1371/journal.pone.0023040)
Supplement: Protocol S4 — Protocol appendix 3: Patient Invitation from GP; version 2. (DOC) [file pone.0023040.s005.doc]

# Practice Header

**Name**

**Add 1**

**Add2**

**Postcode**

**Date**

**The Aberdeen Behaviour Change (ABC) Weight Loss Study**

Dear

We are helping researchers at the University with a study and would like to invite you to take part. The study will test a new intervention to help people to lose weight.

**What is involved?** People who take part will be divided into two groups. The group each person is put into will be decided by chance, like tossing a coin. One group will receive written information about weight loss and physical activity, and the other group will be invited to take part in six group sessions (90 minutes each) at Aberdeen Royal Infirmary. The sessions will give help and training in how to change physical activity and eating habits in order to lose weight.

If you decide to take part in the study you would be asked to visit the hospital to see a research nurse at the start of the study and again at the end. The nurse would measure your height and weight, and would ask you to take a simple fitness test. Volunteers would also be asked to complete a short questionnaire.

Please read the information sheet enclosed with this letter. If you would like to take part, please fill in the **CONSENT FORM** and send it to the researchers using the pre-paid envelope. If you would like more information before deciding, please fill in the **REPLY SLIP** and send to the researchers in the pre-paid envelope. A member of the research team will then contact you to speak to you about the study.

If you have any questions **please** **contact the ABC Weight Loss Study Office** on telephone 01224 272150, email [ABCstudy@abdn.ac.uk](mailto:ABC-study@abdn.ac.uk), or contact Dr Falko Sniehotta, School of Psychology, William Guild Building, King’s College, University of Aberdeen, AB24 2UB.

Thank you for taking the time to read this letter.

Yours faithfully

The Doctors

Xxx Medical Practice Study ID
