# Supplementary material for: Randomised Controlled Feasibility Trial of an Evidence-Informed Behavioural Intervention for Obese Adults with Additional Risk Factors
Source: PLoS One. 2011 Aug 29;6(8):e23040. doi: 10.1371/journal.pone.0023040 (PMC3163575; doi:10.1371/journal.pone.0023040)
Supplement: Protocol S5 — Protocol appendix 4: Participant Information; version 4. (DOC) [file pone.0023040.s006.doc]

**A**berdeen **B**ehaviour **C**hange for Weight Loss Study

PARTICIPANT INFORMATION SHEET

The purpose of this study is to test the effectiveness of a new behaviour change intervention for weight loss.

*Please take time to read this information leaflet and discuss it with your family and friends if you wish. Do not hesitate to contact us using the contact details below if there is anything you do not understand or if you would like more information.*


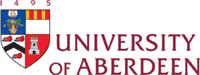


**ABC Weight Loss Study**

1. **Full title of project**Feasibility study for a randomised controlled trial of a behavioural intervention to reduce weight in obese adults
2. **Invitation**You are being invited to take part in a research study. Before you decide, it is important for you to understand why the research is being done and what it will involve. Please take time to read the following information carefully and discuss it with others if you wish. Do feel free to ask us anything that is not clear or if you would like more information.

   Thank you for reading this.
3. **What is the purpose of this study?**Losing weight involves making long-term changes to lifestyle behaviours such as diet and physical activity. These changes are difficult to achieve without assistance and we need to find new ways to help people with weight problems make these changes.
   We want to find out if a new behavioural treatment for obesity is more effective for weight loss compared to current NHS treatment. This new intervention focuses on learning effective strategies for long-term diet and physical activity changes.
4. **Do I have to take part?**No. It is up to you whether you decide to take part. We are happy to answer any questions you may have before you decide. If you wish to take part, you will be asked to sign a consent from, a copy of which will be given to you together with this information sheet. You can change your mind at any time and decide not to participate. The standard of care you receive will not be affected in any way if you decide not to take part or if you take part now and decide to withdraw from the study later. You need not give any reason for declining or withdrawing from the study. If you withdraw from the study any information already gathered from you will be either kept securely and confidentially or destroyed if you wish.
5. **What will happen if I take part?**Sometimes because we do not know which way of treating patients is best, we need to make comparisons. Study participants are therefore put into groups and then compared. The groups are selected by a computer which has no information about the individual (i.e. by chance). Patients in each group then have different treatments and these are compared.

   In this study you will have a 2 in 3 chance of being in a **face-to-face treatment group** and a 1 in 3 chance of being in a **written advice group**. Both treatments will be in addition to standard NHS care, you will not experience any changes in your regular health care.

   If you are in the **face-to-face treatment group** you will be invited to attend 5 weekly group sessions and one follow up meeting 3 weeks later in which you will learn about strategies for changing diet and physical activity behaviours, have the opportunity to discuss issues you experience and receive support in making these changes. You will receive a modern stepcounter (pedometer) to monitor your walking throughout the study.
   Each session will take about 90 minutes. These sessions will be delivered by a trained nurse, dietician or psychologist. Sessions will be tape-recorded and later written down for research purposes. Individual participants will not be identified from these transcripts.

   In the **written advice group** you will receive information leaflets about behaviour change and weight loss by post.

   All participants will also be seen by a qualified health professional at Aberdeen Royal Infirmary at the beginning and the end of the study, in order to measure your weight, height, waist and hip circumference. To understand how body composition changes over the study, we will also measure the overall body fat with a standard handhold monitor measuring the body’s resistance to a high frequency pulse. This measurement is completely safe and monitors similar to the ones we use can be bought in Boots the chemist.
   We will also ask participants at the beginning and end of the study to walk for six minutes choosing their own intensity and being allowed to rest. The distance walked in these 6 minutes is a good indicator of fitness that is important for everyday activities. If you decide to take part in this research, we will give you a call to arrange a convenient time for these meetings.

During these appointments we will also ask you to complete a brief questionnaire about: your experiences of the study; your everyday behaviours; your views and strategies towards these behaviours; and your weight at the beginning and the end of the study. In addition, we will send you a similar questionnaire about 12 weeks after the first questionnaire by post with a stamped return envelope. Each questionnaire should take less than half an hour to fill in and if needed, we will assist you completing it.

1. **What are the possible disadvantages of taking part?**
   There are no anticipated disadvantages or risks from this study.
2. **What are the possible benefits of taking part?**You will contribute to improving treatment for people with weight problems. Without research we cannot know if a new treatment is more effective or not. Effective weight-loss interventions are needed to help people change their lifestyle behaviours and address weight and health problems.
   You might also find that participating in this study helps you making desired behaviour changes and losing weight.
3. **What if new information becomes available?**Sometimes during the course of a research study, new information becomes available about the treatment that is being studied. If that happens, a member of the research team will contact you to let you know about the choices available to you. However, we are not aware that any new information is likely to become available before the end of this study.
4. **What if something goes wrong?**We do not expect any harm to come to you by taking part in this research. However in the unlikely event that you would be harmed by taking part in this research there are no special compensation arrangements. If you are harmed due to someone’s negligence, then you may have grounds for a legal action but you may have to pay for it. If you wish to complain about any aspect of the way you have been approached or treated during the course of this study, the normal National Health Service complaints mechanisms may be available to you.
5. **Will my taking part in this study be kept confidential?**Yes! All information that is collected about you during the course of the research will be kept **strictly confidential**. The identification information that you give us will be separated from your answers to the questionnaires and will only be linked using a secret unique study number. Any information about you that leaves the hospital or research unit will have your name and address removed so that you cannot be recognised from it.
6. **What will happen to the results of the research study?**Results obtained in this study will be published in medical and academic journals, and presented at academic conferences. You will not be identified personally in any report or publication. The findings of this study will inform a subsequent bigger study to test the effectiveness of this new intervention.
7. **Why have I been invited?**
   You GP practice has agreed to help with this project. You have been invited as your records show that you might benefit from losing weight.
8. **Who is organising and funding this research?**This research is funded by the Scottish Government, Chief Scientist Office. The study is being organised by the staff of the ABC Weight Loss Study office at the University of Aberdeen.

   The funds are only available for the expenses necessary to run this study, the salaries of the researchers and staff employed. No-one will benefit financially from this research.
9. **Who has reviewed the study?**
   This study has been approved by the North of Scotland Research Ethics Service. The science has been reviewed and approved by the Scottish Government Chief Scientist Office.
10. **What do I do now?**
    If you are happy to take part in this research, please sign the enclosed CONSENT form and return it to the researcher using the freepost envelope.
    If you would prefer to speak to one of the researchers before making a decision, please return the attached REPLY SLIP to the researchers or call/email the study office using the details below.

**You should keep this information sheet.**

**If you should agree to enter this study, please sign the enclosed consent form and we will return a copy to you.**

Thank you very much for reading
this information sheet.

If you have any questions or would like more information, please contact

ABC Weight Loss Study Office
phone: 01224 272150
email [ABCstudy@abdn.ac.uk](mailto:ABC-study@abdn.ac.uk).

or the Chief Investigator
Dr Falko Sniehotta, College of Life Sciences and Medicine, School of Psychology, William Guild Building, King’s College, Aberdeen AB24 2UB.
