# Supplementary material for: Randomised Controlled Feasibility Trial of an Evidence-Informed Behavioural Intervention for Obese Adults with Additional Risk Factors
Source: PLoS One. 2011 Aug 29;6(8):e23040. doi: 10.1371/journal.pone.0023040 (PMC3163575; doi:10.1371/journal.pone.0023040)
Supplement: Protocol S6 — Protocol appendix 5: Reply Slip; version 2; 21 07 2009. (DOC) [file pone.0023040.s007.doc]

**A**berdeen **B**ehaviour **C**hange for Weight Loss Study

## STUDY REPLY SLIP

This is an invitation to take part in the ABC study. Please read the attached information carefully. Please then tick one of the boxes below and send to us using the pre-paid envelope.

Please tick one box

Yes, I would be interested in taking part in this study. Please contact me

If you would like to take part, please also complete and return the CONSENT FORM

| My telephone number: |  |
| --- | --- |
| or |  |
| Best time to call |  |
| My email address: |  |

No thank you, I do not want to take part in this study

Please return this slip in the enclosed postage paid envelope

**Thank you for considering taking part in this study**

ID
