# Supplementary material for: Randomised Controlled Feasibility Trial of an Evidence-Informed Behavioural Intervention for Obese Adults with Additional Risk Factors
Source: PLoS One. 2011 Aug 29;6(8):e23040. doi: 10.1371/journal.pone.0023040 (PMC3163575; doi:10.1371/journal.pone.0023040)
Supplement: Protocol S7 — Protocol appendix 6: Consent Form; version 3. (DOC) [file pone.0023040.s008.doc]

**CONSENT FORM**

**Please initial the**

**boxes**

1. I confirm that I have read and understand the information sheet for the above study. I have had the opportunity to consider the information, ask questions and have had these answered satisfactorily.
2. I understand that my participation in this study will involve two appointments with a health professional at Aberdeen Royal Infirmary and completing questionnaires at the beginning, in the middle and at the end of the study. My responses will be anonymised. I understand that once the study is complete the questionnaires will be destroyed and I give consent that my anonymised data will be made available to researchers of the University of Aberdeen.
3. I understand that this study will involve allocation by chance to group treatment or receiving written information. I may be invited to take part in a six sessions of a group treatment aiming to change dietary and physical activity behaviours relevant to weight loss. These group sessions will be tape-recorded and later anonymously written down for research purposes. During this treatment, I would also be asked to wear a step-counter (pedometer). I consent for my weekly step count data and body weight measures to be made available to the researchers from the University of Aberdeen in an anonymous manner.
4. I understand that relevant sections of my medical notes, and data collected during the study, may be looked at by individuals from the University of Aberdeen, from regulatory authorities or from the NHS Trust, where it is relevant to my taking part in this research. I give permission for these individuals to have access to my records for research, audit and monitoring.
5. I understand that my participation is entirely voluntary and that I am free to withdraw at any time without giving any reason, without my legal rights being affected. I understand that all information provided by me will be held confidentially. The information will be securely stored in the University of Aberdeen. In accordance with the Data Protection Act I can have access to my information at any time.
6. I agree to take part in the above study.

| Name (please print) |  | |
| --- | --- | --- |
| Signature |  | |
| Date |  |  |
| Address |  | |
|  |  | |
| Postcode |  |  |
| Telephone no |  |  |
