# Supplementary material for: Randomised Controlled Feasibility Trial of an Evidence-Informed Behavioural Intervention for Obese Adults with Additional Risk Factors
Source: PLoS One. 2011 Aug 29;6(8):e23040. doi: 10.1371/journal.pone.0023040 (PMC3163575; doi:10.1371/journal.pone.0023040)
Supplement: Protocol S8 — Protocol appendix 7: Standard Operating Procedures for Blood pressure and Heart Rate Measurement, Clinical Research Facility, University of Aberdeen. (DOC) [file pone.0023040.s009.doc]

**
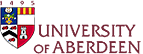

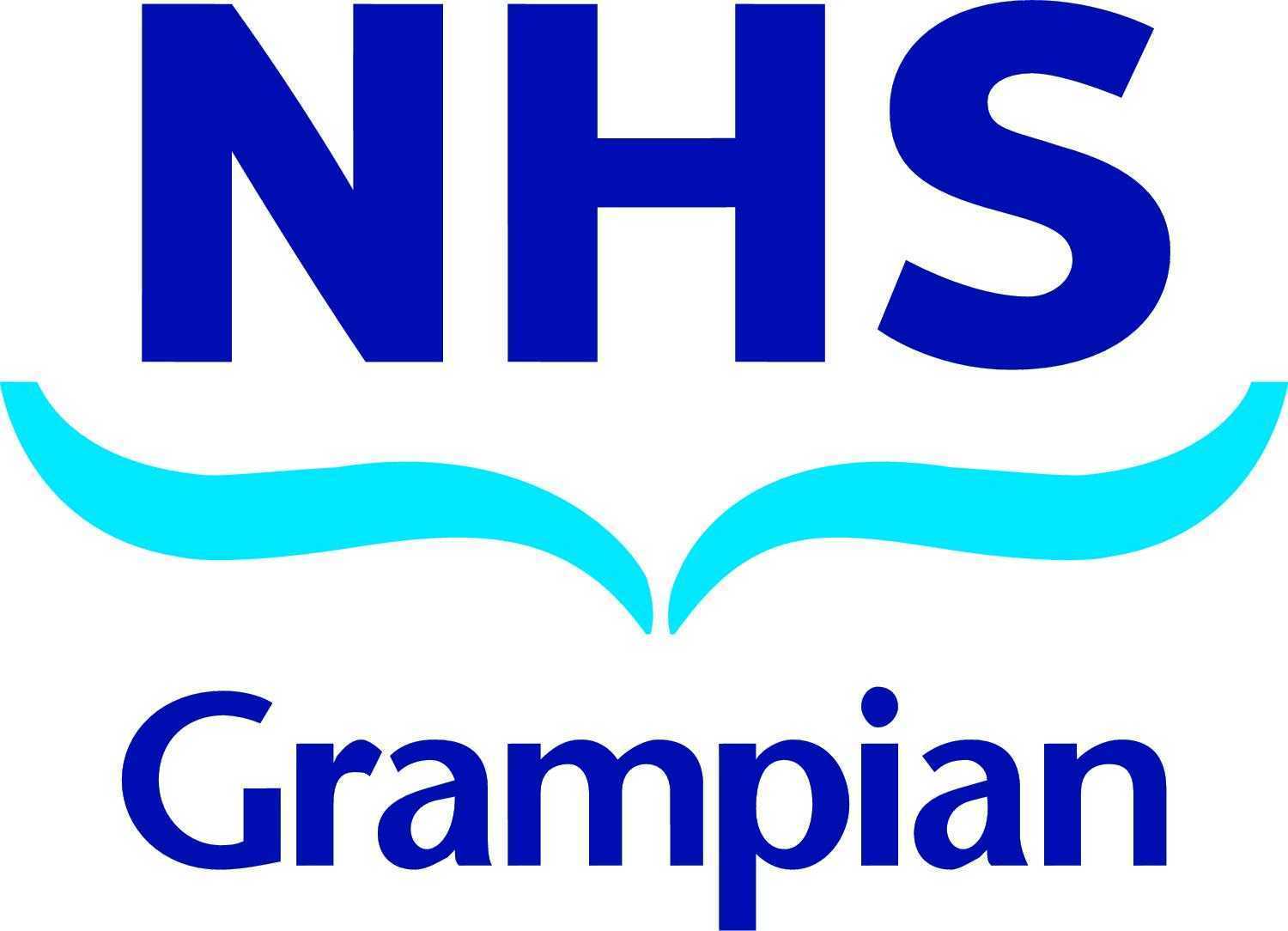
**

**Standard Operating Procedure**

**SOP No.: Version No.:**

**Title: Standard Operating Procedure for measuring and recording blood pressure and resting pulse using electronic devices**

Author: Dr Marie Labus, Clinical Facilities Manager

Approved by (1): Date:

(Professor Phil Hannaford, University of Aberdeen)

Approved by (2): Date:

(Professor Alison Macleod, R & D Director, NHS Grampian)

QA Approval: Date:

(QA Manager, University of Aberdeen & NHS Grampian)

Issue Date:

Date Effective:

**This Standard Operating Procedure will be reviewed 2 years from Issue Date.**

Review Due Date:

Document History:

| **Version No.** | **Description of Changes** | **Date Approved** | **Date Effective** |
| --- | --- | --- | --- |
| **01** | **New Document** |  |  |

1. **PURPOSE/INTRODUCTION**

1.1 The measurement of blood pressure (BP) and resting pulse gives an overall general assessment of the state of the cardiovascular system. Blood pressure is however susceptible to physical stress and emotion and a measure of the usual blood pressure will only be obtained if the participant is comfortable and relaxed. It is important to obtain as accurate a reading of usual blood pressure as possible.

1. **SCOPE**

2.1 To describe the procedure for the measurement and recording of blood pressure and resting pulse at the study clinic visit in accordance with section 2.13 of ICH GCP guidelines and to ensure uniformity within the Clinical Research Facility.

**3. RELATED DOCUMENTATION**

- Obtaining informed consent from incapacitated adults and children (**INSERT SOP NUMBER**)

1. **REFERENCES**

- ICH/GCP Directive/guidelines [**http://www.ich.org/LOB/media/MEDIA482.pdf**](http://www.ich.org/LOB/media/MEDIA482.pdf)
- User manual for the Omron HEM-7051T digital BP

1. **RESPONSIBILITIES**

5.1 Research staff should be trained in the measurement of blood pressure and resting pulse using the standardised equipment supplied. The recording of these measurements should be accurate and follow section 2.10 of the ICH GCP guidelines.

1. **PROCEDURE**

6.1 **Equipment**

- Omron HEM-7051T digital BP monitor. (N.B. If using different study specific Blood Pressure monitors, refer to appropriate Instruction Manual)
- BP cuff × 2: 1 medium and 1 large
- Mains adaptor also provided but if using remove batteries.
- Batteries × 4: AA size, rechargeable or non rechargeable
- Chair next to a table for the participant to sit on

6.2 **General points**

- Ensure written informed consent for the specific study has been obtained before performing any procedure with participants.
- The readings of systolic and diastolic blood pressure and pulse rate are usually required to be entered directly into the appropriate section of the study specific computer or paper Case Report Form (CRF). If a paper CRF is used, use a black ballpoint pen and print all entries legibly. Print off results for source data. These paper results should be photocopied every so often as it may fade over a period of time.
- Accurate readings of usual blood pressure can only be obtained after the participant has been sitting comfortable and quietly for at least 10 minutes.
- Every effort should be made to put the participant at ease.
- Measurement is generally taken from the left upper arm as this is nearest to the heart.

**6.3** **Preparation**

**Before the clinic visit:**

- Check mains adaptor correctly fitted to machine and electricity supply, ensuring no batteries in machine.

OR

- Check that the batteries are charged. Turn on the monitor. If the device is displaying the symbol of a battery with a cross above it, this indicates a low battery. The batteries should be replaced.

**During the clinic visit:**

- Explain to the participant that you are going to perform the procedure and obtain verbal consent to continue
- Ask the participant if he/she has had any of the following in the previous hour
  - Smoked either a cigarette or pipe
  - Drank any coffee, tea or cola
  - Participated in any arduous activity

If the participant has answered yes to any of the above, measurement of BP may have to be delayed until later in the procedure schedule. Any changes to the schedule should be documented in the comments section of any study specific computer or paper CRF being used for the study to include at what point in the visit the measurement was actually taken

- Ask the participant to sit in a chair next to the blood pressure monitor and relax. Legs should not be crossed.

Ask him/her to remove or loosen any clothing covering the upper arm to be used for the blood pressure reading. This would normally be the LEFT arm. Ask the participant if they are right or left handed. It is preferred that the cuff is placed on the non-dominant arm (usually the left), unless there is an indication that this might not be appropriate i.e. previous breast surgery.

- If the right arm is used, this should be recorded in the comments section in the study computer or paper CRF being used for the study.
  1. **Process**
     - - - Explain briefly to the participant what is involved i.e. that they will feel the cuff tighten and relax on their arm but that this should not be too uncomfortable.
         - Apply the appropriate cuff to the upper arm by slipping the cuff over the hand and forearm. (If there is uncertainty as to which cuff to use, use a tape measure to measure the circumference of the mid point of the upper arm and select the correct cuff using the table below). It is important that the cuff covers 80% of the circumference at the midpoint of the upper arm.

| **Circumference of upper arm** | **Size of cuff** | **Size of Cuff bladder** |
| --- | --- | --- |
| 22-32cm | Regular | 12 x 22cm |
| 32-42cm | Large | 15 x 29cm |

- - - - - Rotate the cuff round so that the green marker is over the brachial artery. The brachial artery is located by pressing with 2 fingers about 1 inch above the elbow crease on the inside of the left arm and where the pulse can be felt most strongly.
        - Make certain that the cuff is snug around the arm and making good contact with the skin.
        - Rest the arm, with the palm facing upwards on a pillow on the table next to the participant to ensure that the arm is at heart level.
        - Press the “On/Off” button to turn the monitor on.

**REST THE SUBJECT FOR FIVE MINUTES AT THIS STAGE**

- After 5 minutes ask the participant not to move or speak for a minute while the recording is being taken.
- When heart symbol is displayed on the monitor display screen, press the ‘Start’ button
- A reading of both blood pressure and pulse will be obtained.
- If E appears on the screen and the cuff deflates remove the cuff and re-position ensuring that the green marker is over the brachial artery. Check all connections. See Omron 705 IT instruction manual for more help if the E appears again
- If the participant is feels uncomfortable, stop the procedure and try again later in the visit if the participant is willing. Reassure the participant and record the time when the results were obtained, in the appropriate section of any study specific computer or paper CRF being used.
- Read off the first readings for blood pressure (in mmHg) and pulse rate (in beats per minute) from the monitor and enter them into the appropriate fields of the study specific computer or paper CRF being used

**REST THE SUBJECT FOR A MINIMUM OF A FURTHER TWO MINUTES BEFORE REPEATING THE PROCEDURE**

- Repeat the process of measuring the blood pressure and pulse rate and print off and record as described above.
  1. **Completion**
- Press the “On/Off” button to turn the monitor off.
- Remove the cuff from the participants arm.

**7.0 Maintenance**

**7.1 Cleaning**

The cuffs should be cleaned at regular intervals with a damp cloth.

**7.2Calibration**

The Omron HEM-7051T digital BP monitor does not require calibration but remove batteries when not in use.

**7.3Error Messages**

Incorrect measurement is indicated by the letter **E** on the screen. **Refer to instruction manual.**

**Some Reasons for an error could be:**

- Measurement disturbed due to patient moving.

Try: Repeat and ask patient to keep as still as possible.

- The cuff is not fitted correctly.

Try: Reposition cuff and repeat measurement

- Clothing has constricted blood flow.

Try: Remove clothing causing constriction

- There is still air in the cuff when the monitor is switched off.

Try: The unit may be faulty and needs to be examined by Omron.

- The start button was pressed before the heart symbol was displayed.

Try: Check the batteries if the display light does not light up when the start button is pressed or if a sign of a battery with a cross appears through it. The batteries may need changed.
