# Supplementary material for: Randomised Controlled Feasibility Trial of an Evidence-Informed Behavioural Intervention for Obese Adults with Additional Risk Factors
Source: PLoS One. 2011 Aug 29;6(8):e23040. doi: 10.1371/journal.pone.0023040 (PMC3163575; doi:10.1371/journal.pone.0023040)
Supplement: Protocol S9 — Protocol appendix 8: 6 Minute Walk Test Standard Operating Procedures. (DOC) [file pone.0023040.s010.doc]

**Six-Minute Walk Test**

The 6MWT is a test of functional exercise capacity and physical fitness. It is easy to administer, better tolerated, and more reflective of activities of daily living than other walking tests. This test measures the distance that a patient can walk quickly on a flat, hard surface in a period of 6 minutes (the 6MWD). The self-paced 6MWT assesses the submaximal level of functional capacity. Most patients do not achieve maximal exercise capacity during the 6MWT; instead, they choose their own intensity of exercise, and are allowed to stop and rest during the test. However, because most activities of daily living are performed at submaximal levels of exertion, the 6MWD may better reflect the functional exercise level for daily physical activities.

**CONTRAINDICATIONS**

Absolute contraindications for the 6MWT include the following: unstable angina during the previous month and myocardial infarction during the previous month.

A resting heart rate of more than 120, a systolic blood pressure of more than 180 mm Hg, and a diastolic blood pressure of more than 100 mm Hg, are circumstances in which particular care should be taken and the patient should be asked if they feel fit to perform the test. Stable exertional angina is not an absolute contraindication for a 6MWT, but patients with these symptoms should perform the test after using their antiangina medication, and rescue nitrate medication should be readily available.

**SAFETY ISSUES**

1. Oxygen trolley needs to be available on site. A telephone or other means must be in place to enable a call for help.

2. The staff should be trained in cardiopulmonary resuscitation.

3. In the case of patient collapse research staff will immediately commence basic life support and an ambulance will be called.

4. Basic life support will be continued until further instructions by emergency services.

Reasons for immediately stopping a 6MWT include the following:

1. chest pain,
2. intolerable dyspnea,
3. leg cramps,
4. staggering,
5. diaphoresis, and
6. pale or ashen appearance.

If a test is stopped for any of these reasons, the patient should sit or lie. The following should be obtained based on the judgment of the staff conducting the test: blood pressure, pulse rate, and oxygen saturation. Oxygen should be administered as appropriate.

**REQUIRED EQUIPMENT**

1. Countdown timer (or stopwatch)

2. Mechanical lap counter

3. A chair that can be easily moved along the walking course

4. Worksheets on a clipboard

5. A source of oxygen

6. Sphygmomanometer (Blood Pressure Monitor)

7. Telephone

8. Automated electronic defibrillator

**PATIENT PREPARATION**

1. Comfortable clothing should be worn.

2. Appropriate shoes for walking should be worn.

3. Patients should use their usual walking aids during the test (cane, walker, etc.).

4. The patient’s usual medical regimen should be continued.

5. A “warm-up” period before the test should not be performed.

**TEST PROTOCOL**

1. Have the patient stand and rate their baseline dyspnea and overall fatigue using the Borg scale (see laminated print)

*Borg scale instructions:*

“Please grade your level of shortness of breath using this scale.”

Then ask this: “Please grade your level of fatigue using this scale.”

2. Set the lap counter to zero and the timer to 6 minutes. Assemble all necessary equipment (lap counter, timer, clipboard, Borg Scale, worksheet) and move to the starting point.

3. Instruct the patient as follows:

“The object of this test is to walk as far as possible for 6 minutes. You will walk back and forth in this hallway. Six minutes is a long time to walk, so you will be exerting yourself. You will probably get out of breath or become exhausted. You are permitted to slow down, to stop, and to rest as necessary. You may lean against the wall while resting,

but resume walking as soon as you are able. You will be walking back and forth the corridor. You should briefly touch the door before you turn around and continue back the other way without hesitation. Now I’m going to show you. Please watch the way I turn without hesitation.”

Demonstrate by walking one lap yourself. Walk and pivot around the corridor.

“Are you ready to do that? I am going to use this counter to keep track of the number of laps you complete. I will click it each time you turn around at this starting line. Remember that the object is to walk AS FAR AS POSSIBLE for 6 minutes, but don not run or jog. Please start now, or whenever you are ready.”

4. Position the patient at the starting line. You should also stand near the starting line during the test. Do not walk with the patient. As soon as the patient starts to walk, start the timer.

5. Do not talk to anyone during the walk. Use an even tone of voice when using the standard phrases of encouragement. Watch the patient. Do not get distracted and lose count of the laps. Each time the participant arrives at either side of the corridor, click the lap counter once (or mark the lap on the worksheet). Let the participant see you do it. Exaggerate the click using body language, like using a stopwatch at a race.

- After the first minute, tell the patient the following (in even tones): “You are doing well. You have 5 minutes to go.”
- When the timer shows 4 minutes remaining, tell the patient the following: “Keep up the good work. You have 4 minutes to go.”
- When the timer shows 3 minutes remaining, tell the patient the following: “You are doing well. You are halfway done.”
- When the timer shows 2 minutes remaining, tell the patient the following: “Keep up the good work. You have only 2 minutes left.”
- When the timer shows only 1 minute remaining, tell the patient: “You are doing well. You have only 1 minute to go.”

Do not use other words of encouragement (or body language to speed up).

If the patient stops walking during the test and needs a rest, say this:

“You can lean against the wall if you would like; then continue walking whenever you feel able.”

Do not stop the timer. If the patient stops before the 6 minutes are up and refuses to continue (or you decide that they should not continue), wheel the chair over for the patient to sit on, discontinue the walk, and note on the worksheet the distance, the time stopped, and the reason for stopping prematurely.

When the timer is 15 seconds from completion, say this: “In a moment I’m going to tell you to stop. When I do, just stop right where you are and I will come to you.”

When the timer rings (or buzzes), say this: “Stop!” Walk over to the patient. Consider taking the chair if they look exhausted. Note the spot they stopped by writing down the number on the wall closest to the point of stopping.

6. Post-test: Record the postwalk Borg dyspnea and fatigue levels, and ask this: “What, if anything, kept you from walking further?”

7. Record the number of laps from the counter (or tick marks on the worksheet).

8. Record the additional distance covered (the number of meters in the final partial lap) using the markers on the wall as distance guides. Calculate the total distance walked, rounding to the nearest meter, and record it on the worksheet.

Congratulate the patient on good effort and offer a drink of water.

Any questions call the ABC study office Tel. 01224 272150
