# Supplementary material for: Randomised Controlled Feasibility Trial of an Evidence-Informed Behavioural Intervention for Obese Adults with Additional Risk Factors
Source: PLoS One. 2011 Aug 29;6(8):e23040. doi: 10.1371/journal.pone.0023040 (PMC3163575; doi:10.1371/journal.pone.0023040)
Supplement: Intervention Manual S1 — Intervention Manual Session 1. (PDF) [file pone.0023040.s011.pdf]

The ABC Weight Loss Study -Session 1-

Health Psychology Group
University of Aberdeen - August 2009

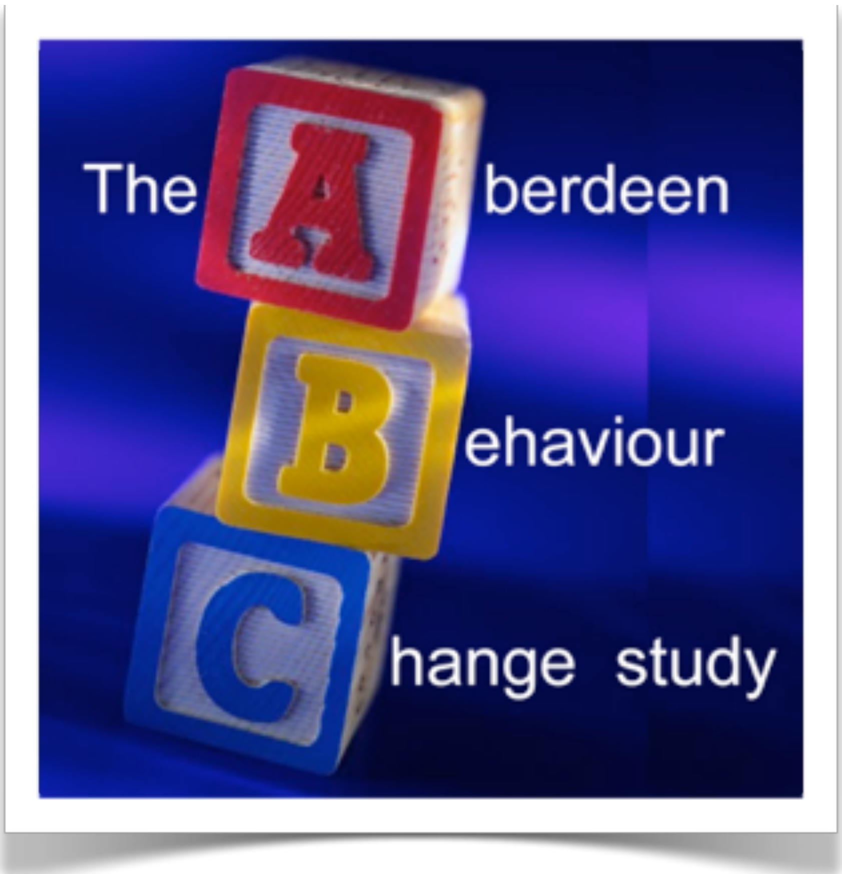

Authored by

Vera Araújo-Soares, Stephan Dombrowski & Falko Sniehotta

(in alphabetical order)

© University of Aberdeen

Table of Contents

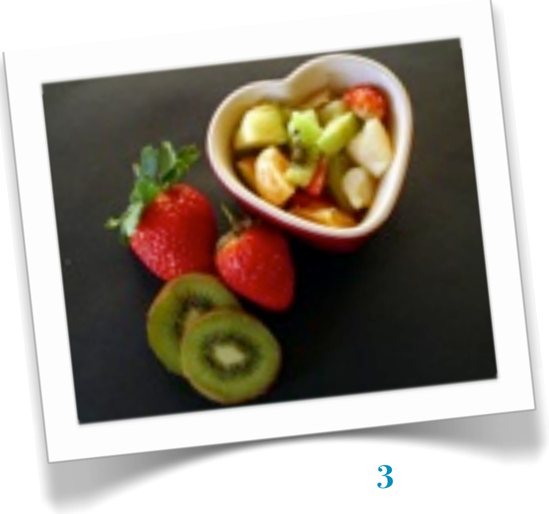

Overview 3

The Session Basics 3

Summary 3

Goals 3

Techniques 3

Materials 4

Recommended time 4

Before the first meeting 4

Activities and procedures 4

Content 5

Introduction: What to expect from the ABC Study 5

Activity 1: Welcome, housekeeping and overall instructions (15 minutes) 5

Activity 2: Explore expectations and outline programme objectives (15 minutes) 5

Activity 3: Formulate an overall goal for the programme (10 minutes) 10

Activity 4: How behaviour affects health - Energy Balance (10 minutes) 11

Activity 5: Introducing the information for specific behaviour change (10 minutes) 13

Activity 6: Discuss group rules and establish agreement (10 minutes) 14

Activity 7: Self-monitoring (15 minutes) 15

Activity 8: Close of session, summary and weekly challenges (5 minutes) 18

Appendix: Session Slides 19

Appendix: Group Norms Sheet 22

Appendix: Pedometer Instruction Sheet 23

Appendix: Healthy Eating Tips 24

Overview

Detailed overview of session content

THE SESSION BASICS

Summary

The first session aims at familiarising the participants with each other and the programme. The programme will be introduced including the rational underlying its development and key assumptions such as a lifestyle approach to sustainable weight loss. Differences between this intervention and other programmes will be established. The main purpose of the first session is that participants form an intention to change (eating behaviours, physical activity (PA) and/or body weight) by setting an overall goal. The overall goal is something that participants would like to achieve through attending the programme. Participants will be asked to self-monitor their eating and PA behaviour over the next week in preparation for the following weeks.

Goals

1. Develop a trusting environment for participants and the facilitator (establishing rapport);
2. Discuss participants' expectations, explain the programme goals and objectives and present an overview of the next five sessions to ensure that participants' expectations and the current programme fit together;
3. Formulate an overall goal participants want to achieve through the programme;
4. Outline the intervention's purpose by explaining its rational and underlying concepts;
5. Define the group rules;
6. Develop an understanding of the relevance of self-monitoring and how to self-monitor eating and PA behaviour over the next weeks.

Techniques

- Intention formation/overall goal setting
- Self-monitoring of behaviour

Materials

- Weekly booklet
- British Heart Foundation (BHF) booklet "So you want to lose weight ... for good"
- Healthy Eating Tips
- Flip-chart
- PowerPoint Slides
- Pedometers
- Pedometer instruction sheet
- Group norms sheet

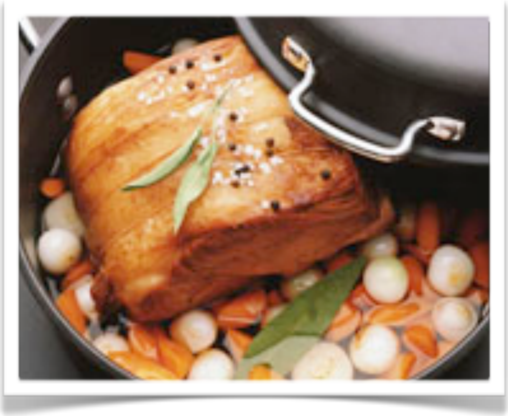

Recommended time

- 90 minutes

Before the first meeting

- Know how to introduce your role and the overall session structure;
- Have a clear idea about the structure of the meeting;
- Have a clear understanding of the behaviour change techniques;
- Get materials ready (see materials section above).

Activities and procedures

In the remainder of this session description you will find a detailed guide to the activities you should facilitate in Session 1.

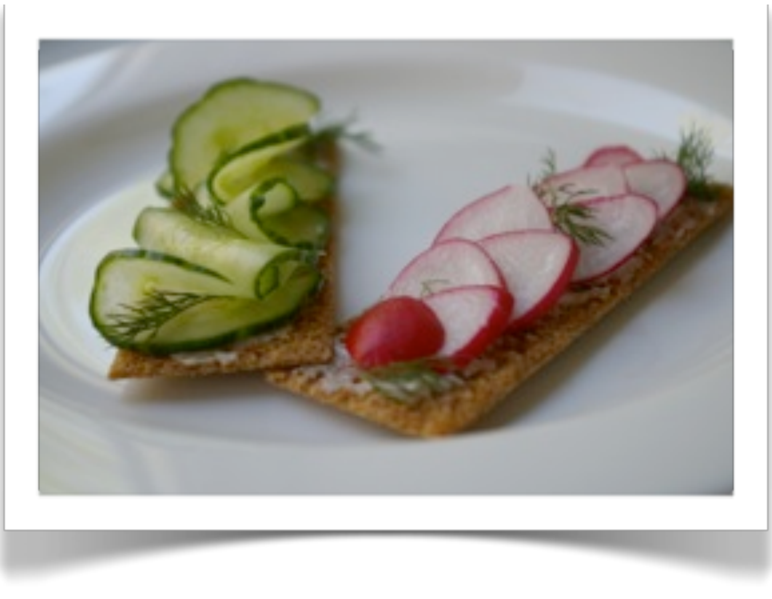

Content

Rationale of the intervention and the importance of self-monitoring

INTRODUCTION: WHAT TO EXPECT FROM THE ABC STUDY

Activity 1: Welcome, housekeeping and overall instructions (15 minutes)

General description: The programme starts with a brief introduction of the housekeeping rules and regulations. Afterwards, each member presents him/herself to the group. Participants could for example share the following information with the group:

See PowerPoint Slide 2&3

- their name (and how they like to be addressed),
- where they are from,
- how they spend their time,
- a brief history about their weight problem,
- what they have done previously to solve this problem,
- If they are on any specific diet.

The facilitator will start the round of introductions to set an example. The goal is to raise a trusting and open climate amongst the participants and the facilitator in order to assist the exchange of experiences. After the introductions the facilitator will briefly outline the plan for the first session before moving on to the next exercise. All further sessions will formally start by an outline of the session content for participants to be aware what to expect at each session.

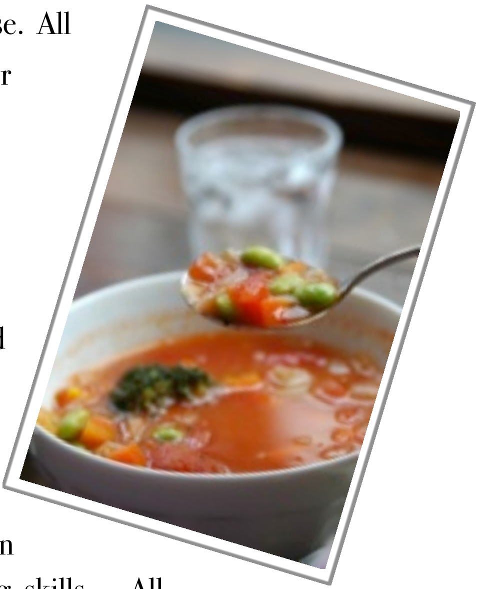

Activity 2: Explore expectations and outline programme objectives (15 minutes)

General description: Group expectations will be elicited, the goals and objectives of this programme will be presented, and misleading expectations will be addressed through a group discussion.

Participants' expectations and general attitude toward the intervention should be elicited individually and listened to using active listening skills. All expectations will be listed on the flip-chart in a concise manner, irrespective of whether these appear realistic or not. The facilitator will then explain that these expectations will be revisited towards the end of the intervention.

The facilitator will then outline the goals and objectives of the current programme and explain the process of change. Furthermore, the novelty of this intervention programme will be stressed at this point. After this exercise, the facilitator will outline the specific goals and objectives of the current programme and explore the fit between what is expected and what is on offer.

The **goals of the programme** are:

- To learn strategies (behaviour change techniques) that have been shown to help participants achieve:
    - long term weight loss
    - weight loss maintenance
    - overall improvements in health (e.g. blood pressure, blood glucose, cholesterol) by inducing lifestyle changes in:
        - eating and drinking behaviours and;
        - PA behaviours

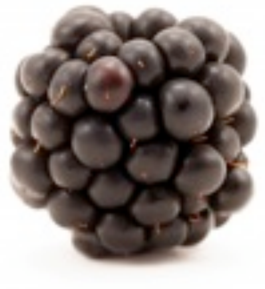

This **programme aims** to achieve these goals by helping participants:

- Plan realistic goals and set them in motion;
- Monitor behaviours, assessing them against a self-set standard;
- Accurately anticipate barriers;
- Develop successful ways of dealing with barriers;
- Maintain achieved lifestyle changes permanently.

By the end of the programme participants should be able to set, follow and maintain realistic goals for behaviour change (eating and PA). They will be able to formulate specific plans and use effective strategies to assist in achieving these plans. By learning the skills needed to establish realistic goals, translate these goals into practice and deal with obstacles participants will be able to face the lifestyle change demands necessary to decrease weight and improve health in a sustained way.

Long-term **objectives of this programme** (i.e. long after the programme finishes) are for participants to have:

- Attained and maintained a healthy diet;
- Attained and maintained regular PA;
- Learned strategies that help maintain changes;
- Lost weight and improved overall well-being;
- Shown improvements in disease risk indicators related to excess weight.

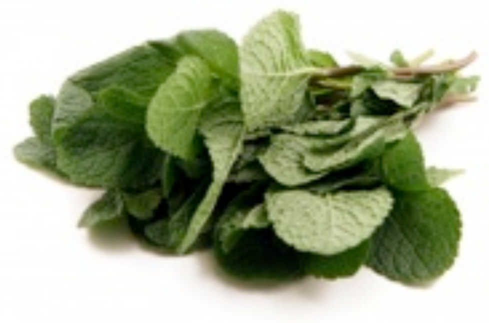

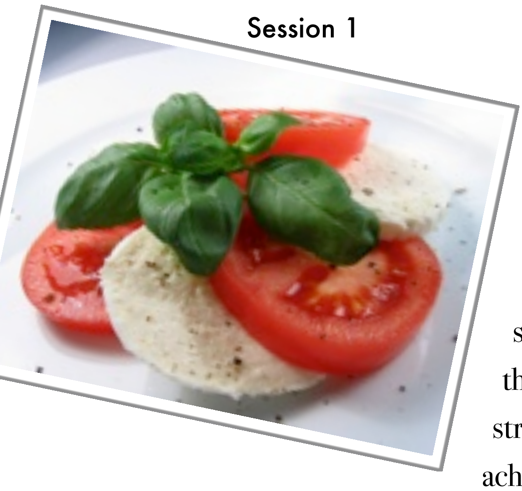

This programme is aimed at people who are interested in changing their weight related behaviours and is designed to help people to self-manage their lifestyle. The intervention manual has been carefully developed by a systematic approach using the best scientific evidence. The best available research over the past 20 years has been systematically reviewed (Dombrowski et al, in submission) to identify the most effective strategies (behaviour change techniques). The strategies presented in this manual were found to help participants achieve significant long-term weight loss and weight-loss maintenance by inducing lifestyle changes in eating and PA behaviours.

Participants will be made aware by the facilitator that there is no magic pill that will help change behaviour and lose weight. Change is not a smooth process and requires time and personal effort. The facilitator will stress this vitally important **long-term perspective on behaviour change**.

See PowerPoint Slide 4

How to perform Activity 2 : Participant expectations, programme goals and objectives

Participant expectations and goals

"Some of you might have attended programmes aimed at helping you to lose weight. All of you must have some expectations of what you want to achieve by attending this programme. Some might even have goals, or aims that they want to achieve. Please take a brief moment to think what it is that you personally expect to, or would like to achieve by coming here" [*facilitator notes responses on flipchart*]

Goals and objectives of the programme

"Thank you very much for sharing you expectations and goals. To make sure that we are all on the same page let me outline what the goals and objectives of this programme are. You will see that these will fit in with what we have listed on the flip-chart."

See PowerPoint Slide 5

"The **programme goals** are:

- To learn strategies (behaviour change techniques) that have been shown to help participants achieve:
    - long term weight loss
    - weight loss maintenance
    - overall improvements in health (e.g. blood pressure, blood glucose, cholesterol) by inducing lifestyle changes in:
        - eating and drinking behaviours
        - physical activity behaviours"

See ppt. slide 6

"To achieve these goals the **programme aims** to help you to:

- Plan realistic goals and set them in motion;
- Monitor behaviours assessing them against a self-set standard;
- Accurately anticipate barriers;
- Develop successful ways of dealing with barrier;
- Maintain achieved lifestyle changes permanently"

continued on next page

"The **long-term objectives**, and by long-term objectives we mean long after the programme finishes, are for you to have:

- Achieved and maintained a *healthy nutrition*.
- Achieved and maintained *regular PA*.
- Learned strategies helping you to *maintain changes*.
- *Lost weight* and feel great.
- Shown *improvements in health*."

See ppt. slide 7

"Before we continue with the next exercise I would like to quickly mention one thing: Whatever our goals and objectives are that we want to achieve, we have to be absolutely clear that behaviour change is not a smooth process. We can only achieve our goals through investing *time* and *personal effort* and by *learning from set-backs*. We will learn to build *slowly and steadily* on each small success to achieve permanent change."

"This programme is not going to suggest a new diet or any miracle cure. Some of you might have seen GPs or a dietician and received a lifestyle advice. All of you will have received leaflets from the British Heart Foundation with information and practical tips on how to improve eating and activity behaviours and habits. This programme is aimed at assisting you to follow this lifestyle advice and to achieve your own goals, helping you to feel in control of your own behaviour change. This programme will also support you in acquiring strategies that will help you maintaining the new behaviours that you choose. We know how difficult changing behaviour is, but these group meetings will provide you with the most effective strategies that more than 20 years of research has brought to light!"

See ppt. slide 8

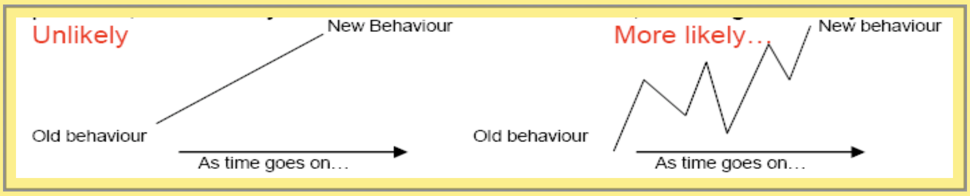

Figure 1: The process of behaviour change over time (*adapted from Health Trainer Manual, 2005 p.11*)

See ppt. slide 9

<u>The programme structure</u>

"So here is the content of the programme for the next weeks. The first five sessions will take place weekly and the last session will take place 3 weeks after the fifth session. All sessions are in this location, and are 90 minutes long. *Session 1* today y is about getting to know each other and the programme. We have just briefly reviewed your expectations and had a look at the goals and objectives of the programme. In the remainder of today we will do two more things: establish group rules for our work together and discuss the value of self-monitoring of behaviour (be your own Agatha Christie's 'Poirot' or 'Miss Marple'; or Arthur Conan Doyle's 'Sherlock Holmes' and 'Doctor Watson', or even 'Taggart')"

continued on next page

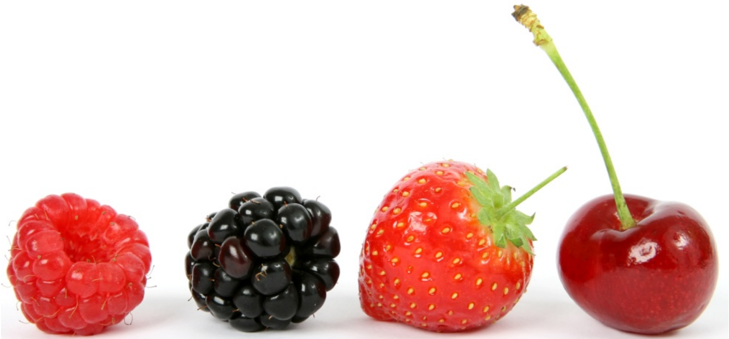

"During *Session 2* we will be assessing your eating behaviours by looking into your self-monitoring records which you will keep over the next week. On the basis of this observation/analysis you will be able to identify your "Opportunities for Change (OC's)". Focusing on the OC's you will be asked to formulate a specific plan for at least one eating behaviour. You will also identify barriers that might get in the way of this specific goal and plan on ways to overcome them."

"In *Session 3* you will review your eating goal and how well you have managed to stick to it during the past week. Possible barriers that might have got in the way of your goals will be identified and new ways of dealing with these will be found. We will then identify and plan at least one new eating goal for the next week."

"In *Session 3* we will also look at the importance of planning for PA."

"During *Session 4* we will be reviewing your eating and PA goals and formulate new plans for both, as well as plans to protect these goals. We will also plan rewards for your achievements. We will learn how to adjust goals and strategies over time to achieve sustainable behaviour change."

"*Session 5* will review the achievements of the 5 previous weeks and highlight all the positive changes that have taken place. During this session we will also talk about falling off the change path, and ways to help you to come back on track."

"Session 6 is the final session of the intervention and will take place 3 weeks after Session 5, and we will have a look at all your achievements over the previous weeks, to see how you managed to integrate the things you have learned during the programme into your everyday lifestyle, and how you managed to keep up all the good changes that you will have made. You will be instructed to think about the weight loss as a process that will take a long time until it produces effects. The great thing about it is: there is something YOU CAN DO in order to reach your weight loss goal."

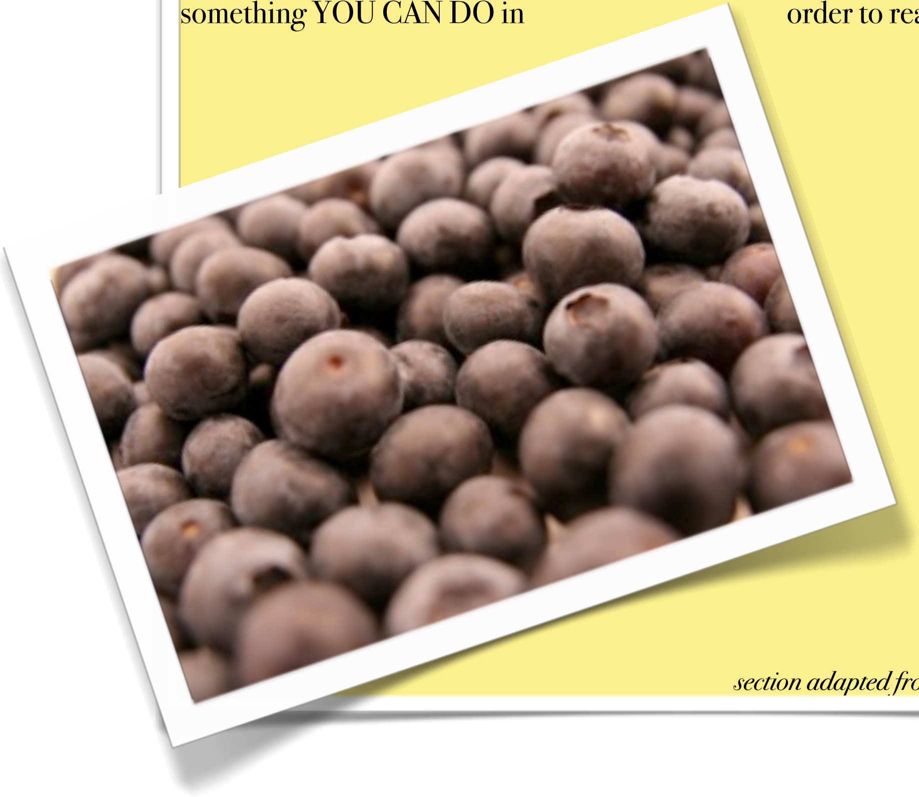

section adapted from Health Trainer Manual, 2005

Activity 3: Formulate an overall goal for the programme (10 minutes)

> *Handy tip*
>
> **Scaling questions** are a very useful tool for assessing the participant's current relationship towards his/her goals and can be used very flexibly in many ways. Typically scales are used to either measure the participant's current state ('I see that you are highly motivated to achieve this goal') or to generate plans regarding how to accomplish a goal ('You rated yourself 5 out of 10. What would need to happen for you to move to 7?').

General description: After clarification of programme goals, objectives and outlining the programme structure the facilitator will ask participants to formulate an overall goal which participants would like to achieve through attending the programme. It should be clear from the previous exercises that the goal should be achievable through changes in eating and PA behaviours. The facilitator will briefly introduce the concept of overall goal setting and participants will note their personal goal in their weekly booklet.

Technique description: What is an overall goal?

Overall goals are things that the participants would like to achieve in the future, by changing behaviour. Having a concrete reason for change, and a long-term goal to aspire to helps participants to prioritise their behaviour change goal over other, often competing behaviours. Identifying overall goal is therefore an important source of motivation for the participant. It helps to put things into perspective and gives a purpose to the ABC study. The participant's general goal is his/her motivator to attend the study in the first place.

How to perform Activity 3: Overall goal setting

See ppt. slide 10

"Now that you are familiar with the goals and objectives of this programme I would like to ask you to formulate an overall goal of what it is that you want to achieve through attending this programme. Please have a look at this weekly booklet (*distribute booklet to participants*). On page 3 you will see a box in which you can note down your overall goal."

"An overall goal can be something that is very general and doesn't necessarily have to be something to do with eating or PA, but it should be achievable through eating and PA. Examples of overall goals are "Fitting into the old cloths again", "Being able to walk up the stars without taking a break" or "Feeling healthy". Make sure that you put something down that can be achieved. The overall goal of "Become as thin as Kate Moss" or "Be able to run 100 metres in under 9 seconds" might not be the best overall goals. Make also sure that you don't put yourself under too much pressure by specifying a deadline. "Fitting into my old cloths within the next 5 weeks" is a very good goal but you would pressure yourself very much. Just omit any time frame on your goals and it should feel a lot easier and more realistic".

"You can check how good your overall goal is by assessing how confident you are that you can achieve it. If you are not confident in your ability to achieve the goal then it might be a better idea to choose something that you think you have a better chance of achieving. Remember, you are the boss and get to decide which goals get set and which ones don't."

Activity 4: How behaviour affects health - Energy Balance (10 minutes)

General description: The facilitator will explain to participants the rationale of the programme. It will be stressed that excess weight is a complicated interaction between social, biological and psychological factors. The importance of individual behaviour in terms of balancing energy intake (e.g. eating and drinking) and energy expenditure (e.g. PA) will be introduced. The facilitator will suggest that despite multiple influences on weight, people are actually in control of that they do. Following on from this it will be stressed that due to this controllability the intervention will focus on behaviour.

See ppt. slide 11

How to perform Activity 4: Behaviour and Excess Weight

"There is no single cause for weight gain, it is the result of complex interactions between social (e.g. environment), biological (e.g. genes), and psychological (e.g. experience) factors."

One key factor in the development and maintenance of obesity is the 'energy balance equation'.

"If the intake of calories from food exceeds the output of energy through metabolism and PA then this leads to the storage of excess energy in the form of fat within the body. In other words, if people eat more than they burn off and do this over long periods of time then obesity will result. It is therefore best to *focus on behaviour as the primary target for change*." (introduce and show Figure 2)

See ppt. slide 12

"Nature is unfair: some people put on weight more easily and have more difficulties in losing it than others. And, yes, genes and social factors play a role. But as you cannot control your genes or society, the energy balance, - what you eat and how much you move - is the only sustainable way for you to lose weight. The good news is that both, a balanced diet and PA are good for your health, over and above their effect on weight."

continued on next page

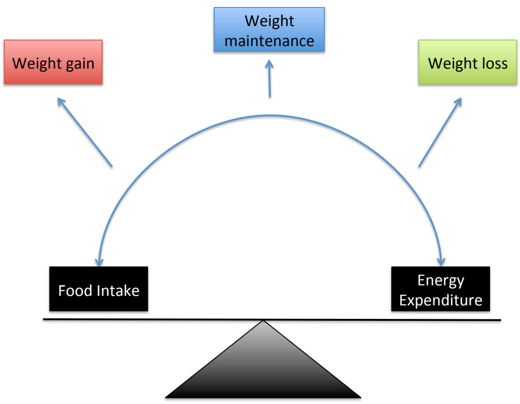

Figure 2: Energy Balance

"To focus attention on behaviour does not mean that we ignore social and biological influences. Rather, this behavioural approach singles out behaviours which help cause and maintain obesity as the prime target for modification. *The major advantages of the behavioural approach are that it can be undertaken and pursued by you according to your own desire and knowledge*. You will be in charge and in control of your treatment!

See ppt. slide 13

Following on from this there are *three fundamental assumptions* that underlie this programme:"

1. *People are able to decide what they want to do (despite influences of social and biological factors).*
2. *People can learn how to change their behaviour should they want to do this.*
3. *People can maintain behavioural changes.*

See ppt. slide 14

"In order to successfully and permanently change lifestyle habits we need *knowledge* and *motivation/willingness* to change. However, knowledge and motivation alone are not enough to change behaviour. In addition we also need a whole *set of skills and strategies* which have to be systematically and consistently used. For example, by setting personal goals to change eating and activity behaviours and using learning strategies to achieve these, you can develop a greater sense of control and improve your health and well-being. *Lifestyle change is a skill that can be learned.*"

"Although the aim of this programme is to lead to positive changes in weight and other health variables (e.g. blood pressure) the primary focus lies on changing eating and PA behaviours. The main reason for focusing on behaviour change is simple: *people can control what they do*. The evidence of more than 20 years of research in the area compiled by this team suggests that weight management interventions mainly achieve changes in weight through altering eating and PA. Therefore, increasing your confidence and skills to be able to change your own behaviour is the primary aim of this programme. The main aims of this programme are to assist you in:

- deciding what to change
- learning how to change
- maintaining these changes in the long term"

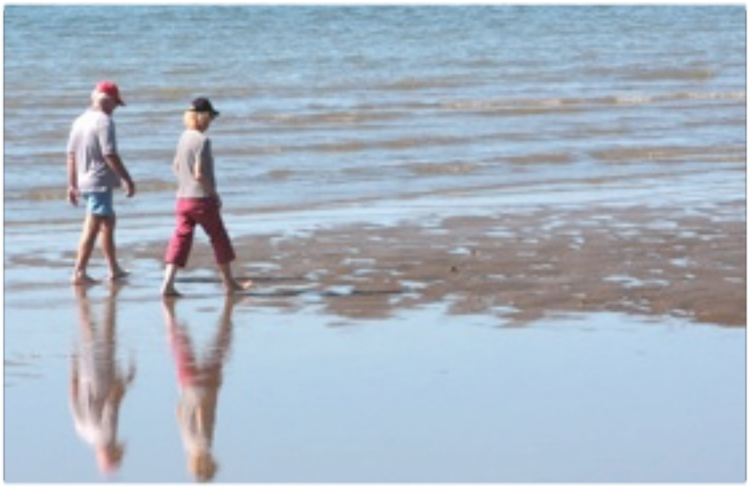

Activity 5: Introducing the information for specific behaviour change (10 minutes)

General description: Following the detailed rationale for why the programme focuses on behaviour change, participants will be introduced to some detailed information about the behaviours that could be changed to lose weight. The facilitator will use/distribute the booklet "So you want to lose weight for good" from the British Heart Foundation (BHF). This booklet contains relevant information about the knowledge needed to change specific behaviours related to weight. This booklet forms the knowledge basis for the intervention. The facilitator will briefly go through the booklet together with participants, and encourage them to read it in detail at home.

How to perform Activity 5: Introducing information on specific behaviours

See ppt. slide 15

"So now that we have established how important behaviour is I would like to hand out a booklet that includes the background information that is needed if we want to change our behaviour to lose weight."

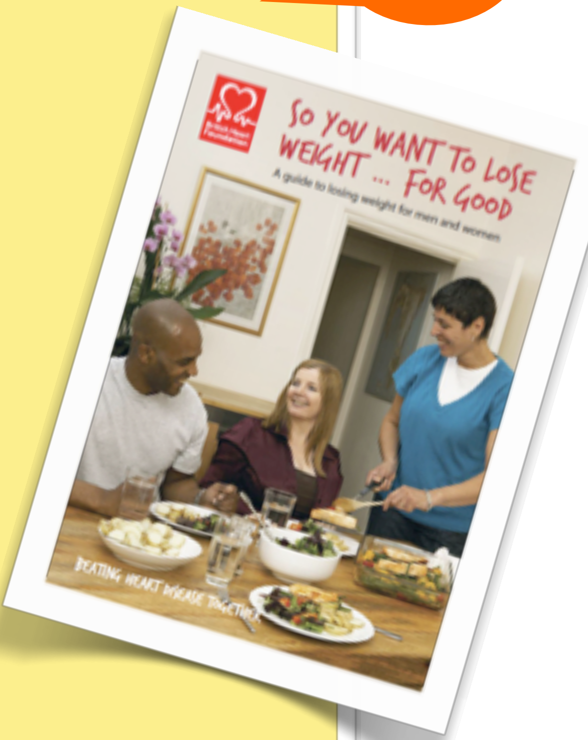

"Some of the things in this booklet from the British Heart Foundation called "So you want to lose weight for good" (see picture on the right), I have already mentioned before. This booklet includes all the information on food that you need to know. For example information on the different food groups you can eat, are presented on the Eat Well Plate as you can see on page 8. It also tells you how much you should eat of each of these groups, and page 10 and includes some information on portion sizes with page 13 illustrating how to measure these. Remember that weight gain is mainly a result of too much energy intake compared with energy expenditure. One way of making sure that you don't consume too much energs, is by making sure that you stick to your food portions in the relevant food groups, as outlined in this leaflet"

"The booklet also has a small section about physical activity on page 23, which we will talk about in detail in Session 3."

See ppt. slide 16

"We won't have time to go through everything that is in this booklet today, and that's why you can all take home your own copy and read it. Also, here I have a summary of some of the most important things that you might want to think about changing [*facilitator distributes the Healthy Eating Tips sheet*]. You can ask me any questions that you have about this next week. Also, if you have any questions during next week you can always call the research office, where Kim will be able to help you out. The number is 01224 272150. Please don't worry if this seems like a lot of information that I am giving you at the moment. There is no knowledge test of this programme and you don't need to know everything that's in there and can still lose some weight. Also, I am sure that you will be surprised about how much you already know and do anyway. "

Activity 6: Discuss group rules and establish agreement (10 minutes)

General description: Next, the group will be briefly introduced to the advantages of working together in a group context, with a view of establishing working rules. Group functioning rules will be introduced, stressing the advantages of these, and each participant should sign a agreement. The rights and duties of each participant and the facilitator will be made clear and mutually agreed. There will also be the possibility for the group to generate new rules not initially included in the document provided.

See ppt. slide 17

How to perform Activity 6: How can the group help

[hand out group rules sheet]
"Group sessions offer multiple *benefits*:
- the group members offer ideas that are different from your own ideas and that might help;
- the relationships established between group members is a valuable experience. You will see that other people have similar difficulties, and you can learn a lot from them."

How to be a good group member?

"To get the best out of your group experience you should try to listen to the suggestions by other members and the facilitator, and at the same time help the other group members by sharing what strategies have helped you in the past. Next, there are some rules that can help working together over the next four weeks."

- "*Listen to what the other group members have to say* with the same attention and care as you listen to the group facilitator.
- Every time you want to state your opinion, please *waiting your turn* to do it.
- *Avoid taking the whole time of the group*. Remember that everyone needs to have a little of the group's time and attention, and that you can learn a lot from the barriers and problems others identify, and with the ways they have used to solve it.
- When you say something to a person in the group, first *think on how you would feel* if someone was to say the same thing to you.
- *Give suggestions instead of criticising.* When you want to say something about the behaviour of another person within the group, please try t to express your opinions and observations in a way that will help the person, instead of judging what the person said or did.
- *Confidentiality* of what is shared within the group is crucial. Please care for it. This will encourage the maximum participation of each person. So please do not share with anyone (e.g. your work mates, friends or family) the content or the author of some of the things said here, unless you have permission to do so.
- *Arriving on time and not missing sessions* are also equally relevant guidelines.
- *Please make sure that your mobile phones are switched off during the session*.

group rules adapted from: McIntyre, T., McIntyre, S., Araújo-Soares, V., Figueiredo, M., & Faria, F. (2004). Programa de Gestão de Stress Laboral [Work Stress: a management programme for professionals]. Universidade do Minho, Portugal

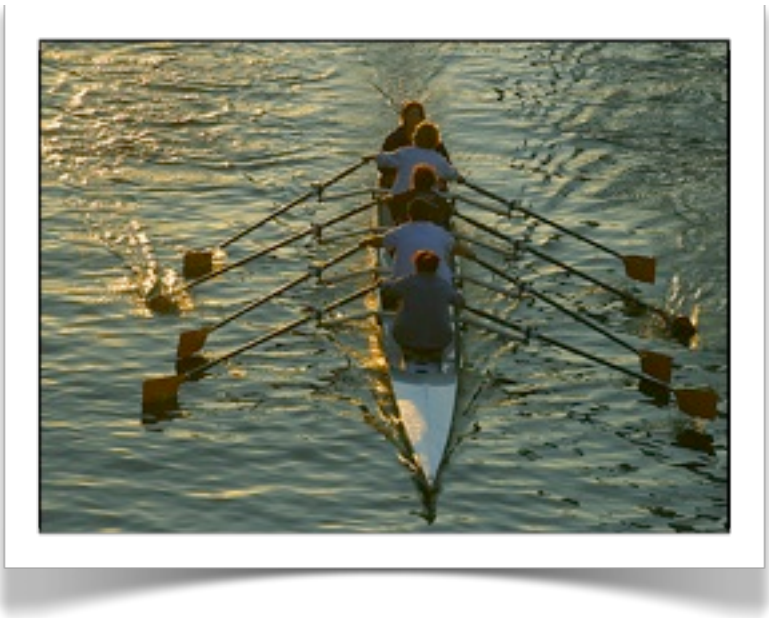

The facilitator will discuss the advantages of these rules and encourage the group to discuss them, and enquire if any important rules are missing and need to be added. Each participant should then sign their own agreement with the rules, which is theirs to keep.

Activity 7: Self-monitoring (15 minutes)

General description: The group will be presented with information on self-monitoring (participants will be referred back to the self-monitoring pages within the weekly booklet). By the end it should be clear to participants that self-monitoring involves the recording and observation of specific behaviours and the contexts in which they occur. This programme will focus on monitoring eating and PA behaviours.

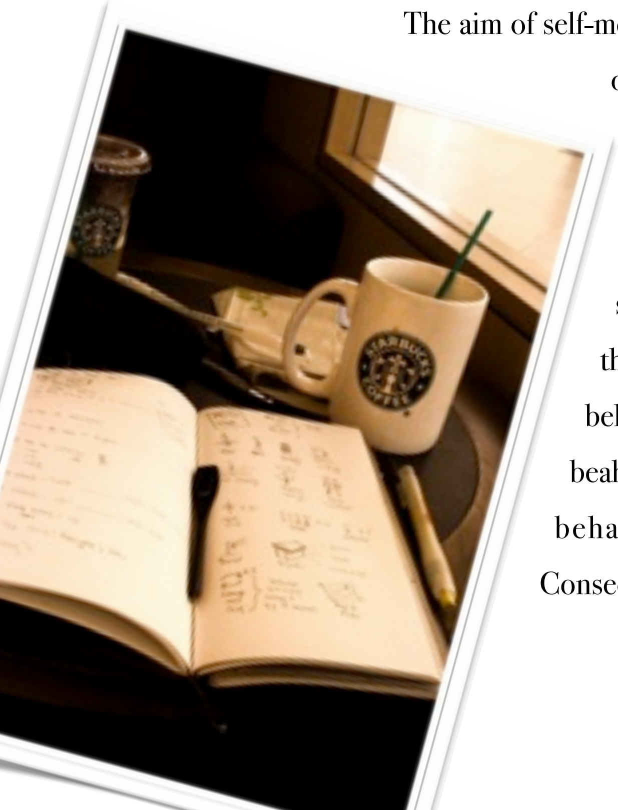

The aim of self-monitoring is for participants to become aware of some of their own behavioural patterns. Self-monitoring also raises awareness how specific behaviours relate to other behaviours (e.g. snacking whilst cooking), emotions/ feelings (e.g. snacking when feeling upset), situations (e.g. snacking when at parties) and thoughts (e.g. snacking when thinking about dinner). What happens before a specific behaviour, called antecedent, and what happens after the specific beahviour, called consequences, have been called the ABC of behaviour change (At first/Antecedent - Behaviour - Consequences). Becoming aware of the things that can trigger a

behaviour (At first/Antecedent), and those that maintain it (consequence) can help to fully understand influences on behaviour. Becoming aware of these influences can help participants identify possible barriers for and opportunities to change.

Description of Technique: What is self-monitoring?

Self-monitoring is to keep a record of behaviour and can be done before and during behaivour change. Self-monitoring can be useful for people who are not sure what changes to make. Just recording your behaviour can help to change it in a desired direction. When a goal has been set, the diary is used to record the changes. Using the diary can make people more aware of when they succeed, or don't succeed, in achieving their desired behaviours (for example, what situations, times of day, associated feelings etc.). This can help inform future plans to make behaviour change easier.

Throughout the programme participants will be asked to monitor their eating behaviour, including "Observations" of important antecedents and consequences of these behaviours. The monitoring pages that participants receive at every session help participants to keep track of their specific dietary behaviours (i.e. food, drinks and snacks and deserts consumed). In addition, participants will also monitor their hunger before every meal, and observations on what happened before and after their eating. Monitoring hunger and observations in addition to specific eating behaviours will help clients to identify antecedents and consequences of their behaviours. It might therefore help to identify behaviours, emotions, situations or thoughts that could either help or be in the way of behaviour change. This information is very crucial in order to identify the behavioural patterns that are important for behaviour change. In addition to detailed monitoring of dietary behaviours, participants will also monitor their PA behaviour (which will be addressed in more detail in Session 3).

See ppt. slide 18

How to perform Activity 7: Why Self-monitoring is so Important

"There are many reasons why it is important to monitor what you do when you are changing your behaviour. Self-monitoring:

1. helps us understand what we do;
2. increases our motivation to change;
3. helps us to understand what triggers and/or reinforces potential problem behaviours;
4. helps us to become aware of our habits.
5. allows us to recall what we have done during the week;
6. helps us to be aware if we are still on track for our goals;
7. helps us to find solutions for our problems."

continued on next page

"You can be your own Sherlock Holmes or Jim Taggart and collect information on your own behaviours. These detectives succeed because they care for the details, these include what comes before and after the case which they are trying to solve, in our case the behaviour change! Self-monitoring helps us understand why we eat, when we eat, know what we eat, and to appreciate the consequences of eating. This will allow us to better understand the opportunities for change and the potential barriers we encounter. It also allows us to reflect upon our own behaviour and inspires us with ways to solve potential barriers for behaviour change. Self-monitoring is therefore a very important part of this programme"

[*facilitator tells participants to look at monitoring pages in the weekly booklet and explains details on filling it in using the provided example page*]

See ppt. slide 19

Additional tips for introducing self-monitoring: The facilitator should introduce the self-monitoring activity following some steps:

1. The monitoring pages should be described in simple terms
   - Participants should collaborate in defining how to do this;
   - Participants should be encouraged to record the event immediately after its occurrence;
   - Support/encourage the recording of thoughts as they are first thought of (e.g. "I feel so fat and ugly, I might as well feed my face, nothing will help me" instead of "I thought that I felt fat and ugly, and that nothing could ever help me, so I have feed my face")- Emphasis should be on the "Here and now".
2. The facilitator should make sure that the participants understood the task;
3. Is important to exemplify how to do it using the flip chart;
4. Reasons for non-adherence to this task should be anticipated and solutions presented. The participant should be informed that he/she could call the facilitator in case of difficulties [give times of availabilities], or participants could team up and be responsible for calling the other.
5. The facilitator should also emphasise that the amount participants invest in self-monitoring in order to understand personal behaviour patterns will be associated with what they can gain from participating in the intervention.
6. To organise the task in a way that it is not considered as a "win or lose" situation, for this:
   - Inform that is hard to do all that we plan to do;
   - Comment that this task *per se* will not lead to immediate change;
   - Reinforce the commitment in trying, and not only the result.

Yoss et al. (1986) suggests some useful questions that can favour a co-operative approach to self-monitoring:

"What thoughts come to your mind when you think of keeping monitoring pages?

Do you think this is important/relevant for your life?

How do you think that monitoring what you eat every day can help you?"

Activity 8: Close of session, summary and weekly challenges (5 minutes)

- "Being a detective": Self monitoring: participants will be self-monitoring their eating as well as their PA levels. For PA monitoring will also include noting step-counts using the pedometer.
- Look into the BHF leaflet "So you want to lose weight for good" in order to remember this information during the next session when discussing their eating goals.

See ppt. slide 20

How to perform Activity 8: Closing session

"So today we have talked about the intervention and the outline of the following weeks. You all set an overall goat that you want to achieve by attending this programme. We discussed the importance that your behaviour has on your weight, and looked at the British Heart Foundation booklet for some more detailed information on which specific behaviours to change. You will all go away and read this booklet in some more detail than we did today. Any questions that you have you can ask me next week. We also established group rules for working with one another over the next weeks. Finally we also talked about the importance of self-monitoring and you will all go away and monitor your eating and PA behaviour over the next week on your diary pages. Does this sound like a fair summary of this session?"

"Before we all leave I would like to mention one last thing: I challenge you to be here next week. Often, in studies like this one where people try to lose weight some people drop out in the initial sessions, and we find that those who drop out do less well compared to those that attend the whole intervention. I would be very pleased to see all of you in the next session, regardless of whether or not you have missed some goals, or whether or not you feel that this is the right thing for you. Just give it a chance and I am sure that you will not be disappointed with what you can achieve."

"See you all next week."

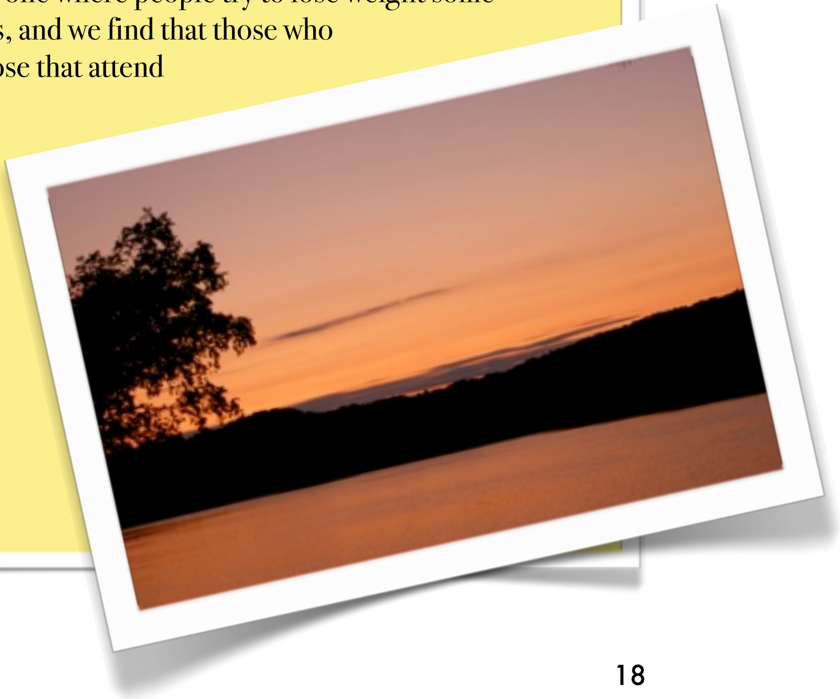

APPENDIX: SESSION SLIDES

Week 1

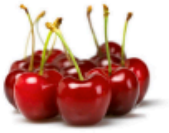

Rationale of this programme and the importance of self-monitoring

UNIVERSITY OF ABERDEEN | NHS Grampian | THE ROBERT GORDON UNIVERSITY ABERDEEN

1

Welcome

- Introduce yourself to the group.
  - Some suggestions on what to share:
    - What's your name?
    - Where are you from?
    - How do you spend your time?
    - Brief history of weight problem/attempted solutions
    - Any specific diet?

2

Today

1. Goals, objectives and expectations
2. Set an overall goal
3. The programme rationale
4. Group rules
5. The importance of self-monitoring
6. Next week

3

Objectives and expectations

What brought you here?

What are your objectives and expectations for this programme?

4

Programme Goals

- Learn strategies that help improve:
  - Long-term weight-loss
  - Weight-loss maintenance
  - Health overall (e.g. blood pressure, cholesterol)
- Through lifestyle changes in:
  - eating and drinking behaviours
  - physical activity behaviours

5

Achieving these goals

This programme will teach you to:

- Plan realistic goals and act on them.
- Monitor your behaviours and compare them with your goals.
- Realistically anticipate barriers.
- Develop ways of dealing with barriers.
- Maintain lifestyle changes permanently.

6

Programme objectives

Long after this programme you will have:

- Achieved and maintained a healthy nutrition.
- Achieved and maintained regular physical activity.
- Learned strategies to maintain changes.
- Lost weight and feel great.
- Shown improvements in health.

7

The change process

- **There is no magic pill leading to automatic change**

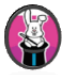

- **Change is not a smooth process**
  - It takes **time** and **personal effort**, and **learning from set-backs** to succeed.
  - We build **slowly and steadily** on each small success.

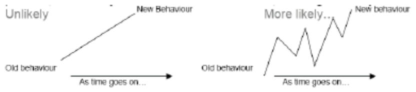

8

The next 5 weeks

Week	Topic
2	Goal setting for eating behaviour
3	Review of eating goals and goal setting for physical activity behaviours
4	Review of eating and physical activity goals and plan rewards
5	Reviewing achievements, generalising activities and supporting maintenance
6	The future starts now

9

Your Overall Goal

- Get out your Weekly Booklet.
- Open p. 3 and write down your Overall Goal:
  - What do you want to achieve through this programme?
- This goal will be your motivator.

10

Programme rationale

- Genes and social factors play a role in weight gain.
- You can't control your genes or society.
- But **you *can* control what you do**:
  - What you eat
  - How much you move.
- You are in charge and in control of your treatment!

11

Programme rationale

Behaviour and Excess Weight

- If energy intake (e.g. eating) is greater than energy expenditure (e.g. metabolism, physical activity) then we gain weight.

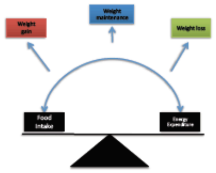

12

Programme rationale

Three Main Ideas:

1. You **decide** what you want to do.
2. You can **learn** how to change your behaviour if you want to do this.
3. You can **maintain** these changes.

13

Programme rationale

- To change lifestyle habits we need **knowledge** (what to change) and **motivation** to change.
- Knowledge and motivation are not enough.
- We also need **strategies** *how* to change.
- Setting goals and using strategies to achieve these, you put you in control of what you do and improve your health and well-being.
- **This programme helps you do what you want to do.**

14

Eating: the Do's

- **British Heart Foundation:**

"So you want to lose weight … For good"

- **It includes all information needed on food:**
  - **Food groups;**
  - **Eatwell plate;**
  - **Portion sizes;**
  - **Label reading;**
  - **Practical tips on some behaviours.**
- **This book is yours to read and use as you please!**

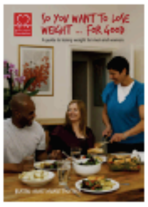

15

Eating: the Do's

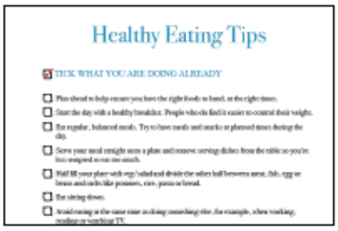

16

The Advantages of a Group

- **Benefits of a group:**
  - **New ideas/inspiration;**
  - **New relations;**
  - **Different/creative ways to solve similar problems;**
  - **Someone who understands**
  - **Sharing the "boat".**
- **These are the proposed rules to function as a group**
- **Any other suggestions?**

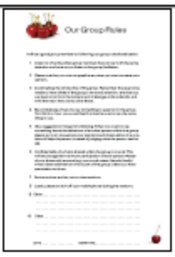

17

The Importance/Advantage of Self-monitoring

Why is Self-monitoring so important?

1. Helps us understand what we do;
2. Increases our motivation to change;
3. Helps us to understand what triggers and/or reinforces potential problem behaviours;
4. Helps us to become aware of our habits.
5. Allows us to recall what we have done during the week;
6. Helps us to be aware if we are still on track for our goals;
7. Helps us to find solutions for our problems.

18

APPENDIX: GROUP NORMS SHEET

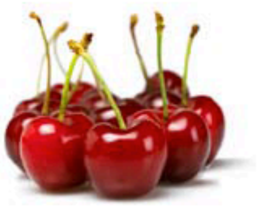

Our Group Rules

I will be a good group member by following our group rules listed below:

1 Listen to what the other group members have to say with the same attention and care as you listen to the group facilitator.

2 Please wait for your turn to speak every time you want to state your opinion.

3 Avoid taking the whole time of the group. Remember that everyone needs to have a little of the group s time and attention, and that you can learn a lot from the barriers and challenges others identify and with the ways they use to solve these.

4 Be considerate when you say something to a person in the group, first think on how you would feel if someone was to say the same thing to you.

5 Give suggestions instead of criticising. When you want to say something about the behaviour of another person within the group, please try to try to express your opinions and observations in a way that will help the person, instead of judging what the person said or did.

6 Confidentiality of what is shared within the group is crucial. This will encourage the maximum participation of each person. Please do not share with anyone (e.g. your work mates, friends, family) what s been said here or who s part of the group unless you have permission to do so.

7 Arrive on time and try not to miss sessions.

8 Lastly, please switch off your mobile phone during the sessions.

9 Other . . . .

. . . .

. . . .

10 Other . . . .

. . . .

. . . .

DATE: . . . . SIGNATURE:. . . . .

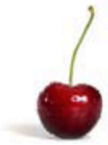

group rules adapted from: McIntyre, T., McIntyre, S., Araújo-Soares, V., Figueiredo, M., & Faria, F. (2004). Programa de Gestão de Stress Laboral [Work Stress: a management programme for professionals]. Universidade do Minho, Portugal

APPENDIX: PEDOMETER INSTRUCTION SHEET

The Study Pedometer Instructions

UNIVERSITY OF ABERDEEN | THE ROBERT GORDON UNIVERSITY ABERDEEN | NHS Grampian

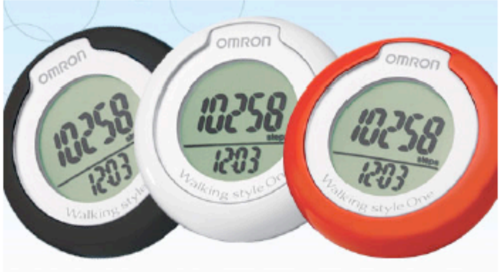

Your pedometer uses the latest technique to count the approximate number ***steps*** you walk every day & stores these for 7 days.

Your pedometer also counts your *aerobic steps* (walking more than 60 steps per minute for more than 10 minutes), the *time* engaged in aerobic activity, the amount of *calories* and grams of *fat* burnt through walking.

All these measures are very interesting, but please **focus on counting your overall steps** for this study . Your programme facilitator will help you set your pedometer to your individual characteristics so that it records the most accurate information for you.

Some tips for using your pedometer:

- Clip your pedometer to your belt or top of your waistband (make sure that it's not too much on an angle).
- Once you attached the pedometer start walking and your pedometer will count your steps. Be patient as it might not count every step straight away, but might count a few steps at a time.
- Using the **MODE** button you change the display from steps counts to aerobic steps counts to kilocalories burnt, to distance walked in kilometres and back to steps.
- Using the **MEMO** button you can look at the 7 day recall of your steps (as well as aerobic steps counts, kilocalories burnt, and distance walked).
- Walking at a consistent pace with regular movements leads to the most accurate step counts.
- You cannot switch your pedometer off. To save energy the step counter switches itself off after 5 minutes (but it still counts your steps!) – to view your recent counts simply press the **MEMO** or **MODE** button and the pedometer switches back on.
- You do not need to reset the pedometer as this occurs automatically.
- See instruction leaflet that came along with your pedometer for additional information.

Your pedometer might not record every single step you take, but it will be accurate enough to show the increase of your walking in relation to previous days, which is most important.

Please remember to wear the pedometer all day long!
Make it a habit by keeping it at the same place every night where you will not forget it next morning!

APPENDIX: HEALTHY EATING TIPS

Healthy Eating Tips

☑ TICK WHAT YOU ARE DOING ALREADY

- ☐ Plan ahead to help ensure you have the right foods to hand, at the right times.
- ☐ Start the day with a healthy breakfast. People who do find it easier to control their weight.
- ☐ Eat regular, balanced meals. Try to have meals and snacks at planned times during the day.
- ☐ Serve your meal straight onto a plate and remove serving dishes from the table so you're less tempted to eat too much.
- ☐ Half fill your plate with veg/salad and divide the other half between meat, fish, egg or beans and carbs like potatoes, rice, pasta or bread.
- ☐ Eat sitting down.
- ☐ Avoid eating at the same time as doing something else, for example, when working, reading or watching TV.
- ☐ Do nothing else while eating (Don't waste the calories – taste and enjoy them.)
- ☐ Eat slowly, concentrate on and really taste what you are putting in your mouth.
- ☐ Pause during meals and put your knife and fork down between mouthfuls.
- ☐ Aim to be the last to finish.
- ☐ Wait at least five minutes after finishing your meal before deciding whether to have second helpings.
- ☐ Clear away any leftovers quickly after the meal.
- ☐ Aim to include at least five portions of fruit and vegetables each day - have some at every meal.
- ☐ Clean your teeth after a meal or when you get the urge to overeat.
- ☐ Write a shopping list and stick to it.
- ☐ Shop on a full stomach.
- ☐ Keep healthy snacks easily to hand (eg, fresh fruit in a bowl, chopped salad or vegetables in the fridge).
- ☐ Choose foods and drinks that are low in fat and sugar and limit sweet and salty snacks.
- ☐ If you drink, moderate alcohol intake. It's high in calories and dissolves good intentions.
- ☐ Watch your portion sizes, especially when eating out.
- ☐ Practise refusing offers to overeat. Learn to say 'No thank you', politely but firmly and convincingly.

adapted from:
British Dietetic Association leaflet 'Want to lose weight and keep it off…?' http://www.bda.uk.com/foodfacts

British Heart Foundation booklet "So you want to lose weight for good" http://www.bhf.org.uk/publications/view-publication.aspx?ps=1000161
