# Supplementary material for: Randomised Controlled Feasibility Trial of an Evidence-Informed Behavioural Intervention for Obese Adults with Additional Risk Factors
Source: PLoS One. 2011 Aug 29;6(8):e23040. doi: 10.1371/journal.pone.0023040 (PMC3163575; doi:10.1371/journal.pone.0023040)
Supplement: Intervention Manual S2 — Intervention Manual Session 2. (PDF) [file pone.0023040.s012.pdf]

The ABC Weight Loss Study -Session2-

Health Psychology Group
University of Aberdeen - August 2009

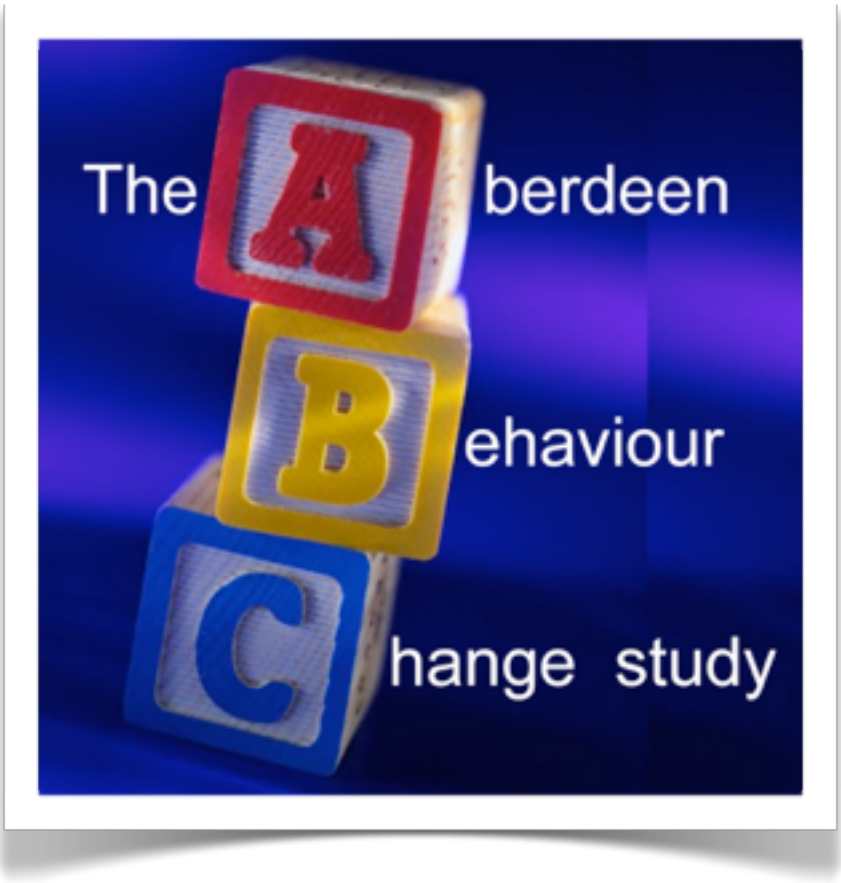

Authored by

Vera Araújo-Soares, Stephan Dombrowski & Falko Sniehotta

(in alphabetical order)

UNIVERSITY OF ABERDEEN

ABERDEEN Health ψ

THE ROBERT GORDON UNIVERSITY ABERDEEN

© University of Aberdeen

Table of Contents

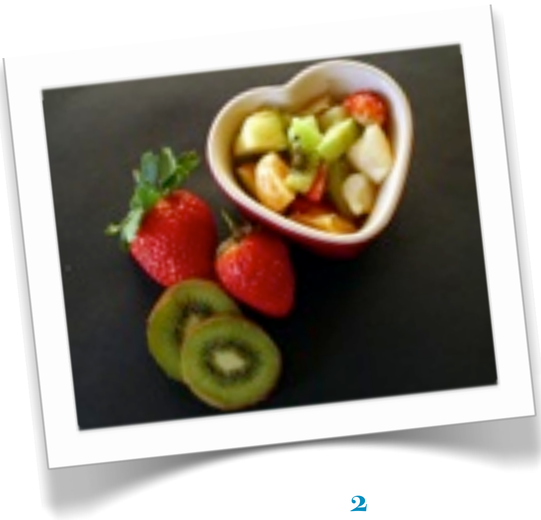

Overview 2

The Session Basics 2

Summary 2

Goals 2

Techniques 2

Materials 2

Recommended time 3

Before the second session 3

Activities and procedures 3

Content 4

Goal Setting for Dietary Behaviours 4

Activity 1: Review of previous week (15 minutes) 4

Activity 2: Formulate an action plan (SMART goal) (20 minutes) 6

Activity 3: Identify and discuss eating barriers (15 minutes) 10

Activity 4: Identify and discuss eating facilitators (15 minutes) 13

Activity 5: Formulate plans to deal with barriers and involve facilitators (15 minutes) 14

Activity 6: Close of session, summary and weekly challenges (10 minutes) 15

Appendix: Session Slides 16

Appendix: Barrier Sheet 19

Appendix: Charting Sheet 21

Overview

Detailed overview of session content

THE SESSION BASICS

Summary

The second session focuses on eating behaviour change and introduces the concepts of specific goal setting and barrier identification. Self-monitoring records for eating and drinking from the previous week will be discussed. Based on these records participants are asked to formulate a specific action plan for at least one eating behaviour. Possible barriers that might get in the way with this specific goal will be identified and a plan for overcoming these barriers will be devised. Participants are asked to monitor their behaviour as well as their goal achievement over the next week.

Goals

1. Self-assessment of eating patterns and identification of "opportunities for change" (using the self-monitoring records of the previous week).
2. Formulation of a specific plan of a desired behaviour that is currently not performed (preferably based on the self-monitoring records).
3. Identification and discussion of *barriers* to maintain healthy food choices.
4. Identification and discussion of *facilitators* to maintaining healthy food choices.
5. Formulation of a coping plan (establishing a personal commitment specifying which barriers might impede goal attainment and what to do when they are encountered).

Techniques

- Specific goal setting *New*
- Barrier identification *New*
- Self-monitoring

Materials

- Weekly booklet
- Flip-chart

- PowerPoint
- Barrier sheets
- Charting sheet

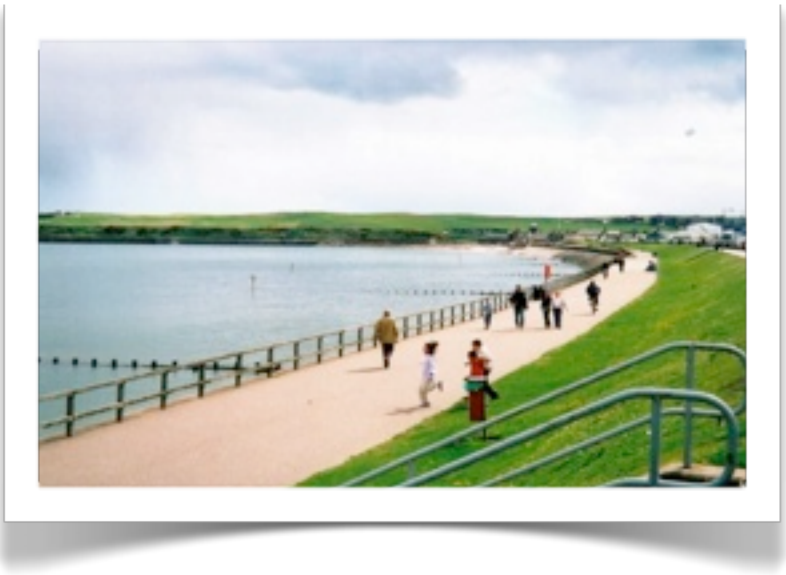

Recommended time

- 90 minutes

Before the second session

- Know how to introduce the second session;
- Have a clear idea about the structure of the second session;
- Have a clear understanding of the behaviour change techniques;
- Get materials ready (see materials section above).

Activities and procedures

In the remainder of this session description you will find a detailed guide to the activities you should facilitate in Session 2.

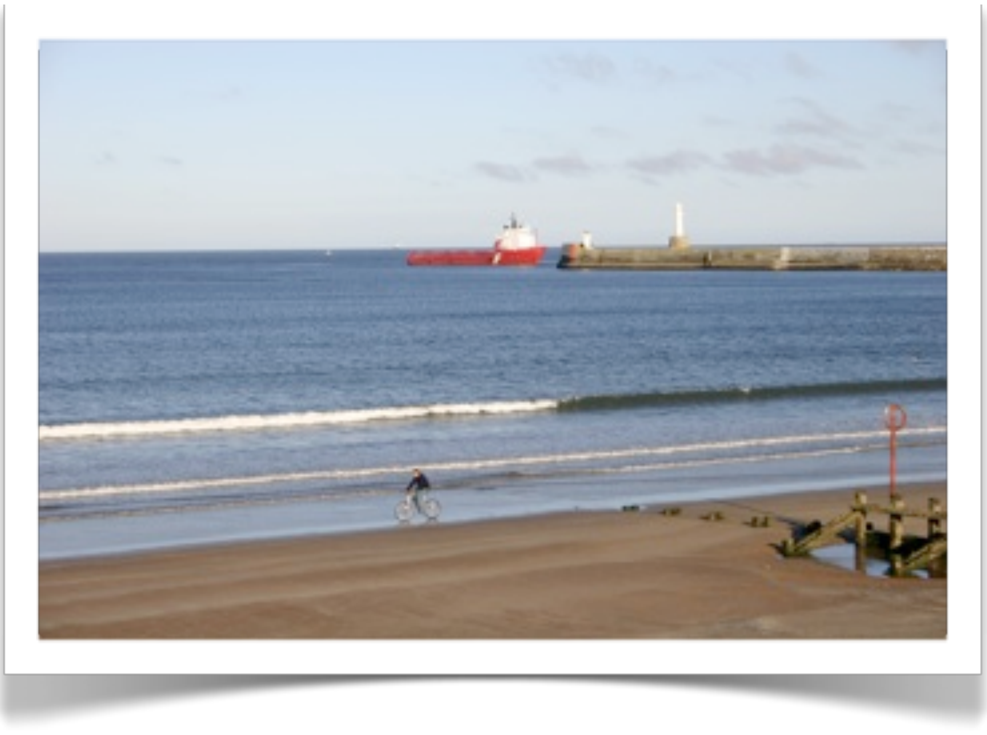

Content

What I Plan to Eat

GOAL SETTING FOR DIETARY BEHAVIOURS

See ppt. slide 2

Activity 1: Review of previous week (15 minutes)

General description: After a warm welcome and a quick overview of the current session, the second session begins with a review of the previous week. Participants were asked to read the British Heart Foundation (BHF) booklet "So you want to lose weight for good" over the last week. Any questions in relation to the information in the booklet will be addressed first, and answered by the facilitator. If questions become too specific (e.g. which type of fruit has the least amount of sugar) the facilitator will remind participants that losing weight is not about becoming a nutritionist, who knows everything there is to know about food, but to manage an energy balance, where energy intake does not exceed energy output. All the relevant information to manage the energy balance is in the BHF booklet. Following the review of the information, self-monitoring records (me as a Detective) will be examined. It is very important to assess participants' reactions to the self-monitoring activity. Participants will be informed that since this session will focus on eating the diary for PA will not be thoroughly assessed in this session. After discussing the monitoring for eating the facilitator should ask who monitored PA, and what main difficulties and patterns were encountered, leaving the full and in-depth discussion of the PA monitoring discussion to Session 3.

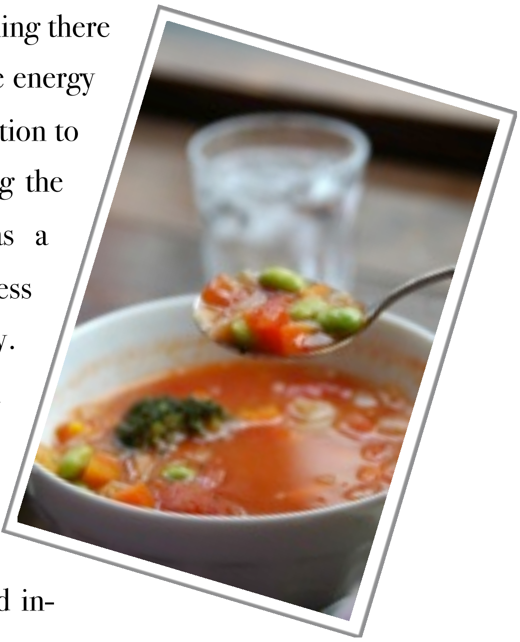

The discussion about the self-monitoring records will focus on what participants ate and drank (types of food and the amount) and what happened before or after their eating (Observations) as recorded in the diary. Participants should try to identify patterns (e.g. fat, carbohydrates/sugar and fruit and vegetable consumption; cooking processes –fried, stew, boiled, steamed- quantities, times), and underline the "best bits" as well as "opportunities for change". This could for example focus on situations, days or times when participants rated their healthy eating as particularly good ("best bits") or as less good ("opportunity for

change") with their healthy eating. Participants that have not brought back the study materials should try and fill it in retrospectively for two starting with today.

It will also be reflected upon what participants were doing/thinking/feeling before starting the eating episode (sometimes called behavioural antecedents) and similarly what was done/ though/felt after the behaviour (sometimes called behavioural consequences). This will allow the identification of possible triggers and/or barriers for healthy eating. It might also be relevant to identify the existence of binge eating episodes during their week.

Each participant will analyse his/her self-monitoring pages individually. After a first analysis he/she will share this with another participant sitting next to him/her, describing the "best bits", "opportunities for change" and possible barriers to follow these opportunities. Participants will report back to the group on these 3 topics. The facilitator will write on the flip chart the top "best bits"; "top opportunities for change" and barriers. Participants will also note their eating and activity in the behaviour change chart.

See ppt. slide 3

How to perform Activity 1: Review of previous week

"Well done everybody for coming back to our second session. I hope that you all had a good week. Today's session will be mainly about changing eating behaviour. Before we start talking about that, lets first see if you have any questions about the booklet that you have read and I would also like to talk about how you got on with your self-monitoring."

"Who has got any questions they would like to ask about the booklet after reading it."

"And how did you get on with your self-monitoring?"

"What I would like to do now is for you to take out your self-monitoring pages and have a look at the eating behaviours you have noted down. Try to identify your "best bits" where your eating has been very healthy and your "opportunities for change" where your eating has been less healthy. If you feel very ambitious you could even calculate the food portions that you have eaten, using our BHF booklet"

"If you haven't filled in your booklet or you have forgotten them, that's not a problem."

"I would like you to pair up now and tell your neighbour about your "best bits" where you were happy with your healthy eating and about an opportunity to change, where you might be able to improve on your diet. If you don't have your filled in booklet just try to do this from memory. For the opportunity to change, also say what you think was the barrier that made it difficult for you to not eat healthily"

"Lets list some of the bet bits, opportunities for change, and barriers on this flip-chart."

[*following this exercise the facilitator distributes behaviour change chart*] Here is a charting paper that you can use to chart how healthy your eating has been and the number of steps each day [*facilitator demonstrates how to fill in the chart*].

Activity 2: Formulate an action plan (SMART goal) (20 minutes)

Based on the assessment of the self-monitoring diary (and in line with dietary recommendations of the BHF booklet) participants will decide on an "opportunity for change" goal. Once it is decided what to change, participants will be required to set a goal to change their behaviour, supported by the facilitator. These goals should be SMART, which stands for **S**pecific; **M**easurable; **A**chievable; **R**elevant, and; **T**ime bound (*adapted from the Health Trainer Manual 2005, p.31-32*).

Technique description: What is a SMART goal?

Formulating and action plan (i.e. SMART goal) increases the likelihood of doing what one wants to do. SMART stands for:

Specific – some goals can be vague and difficult to measure. It is important to set goals that are clear and precise. For example, a vague goal would be "eating healthily". A clear and specific goal would be "I will eat a maximum of 2 muffins a week, one when out for my Wednesday tea with my friend Sarah and the other on Sunday after lunch." To help participants formulate a specific goal they should answer:
What am I going to do?
How am I going to do it?
When am I going to do it?
Where am I going to do it?

Measurable – making the goal specific means that it should be easy to measure whether or not the goal has been achieved. The example, "I will eat a maximum of 2 muffins a week,: one when out for my Wednesday tea with my friend Sarah and the other on Sunday after lunch" is measurable. The participant can record the number of times he/she ate a muffin during the week. It would be hard to measure a vague goal like "eating healthily".

Achievable – Goals should be within the participant's reach. Failing to achieve a goal can have a negative effect on motivation and decrease confidence levels. An example of an unrealistic goal could be: "Eat no chocolate or sweets for the next 7 days". A more realistic goal would be: "Eat no more than 3 portions of chocolate or sweets in the next 7 days". It is important that the first goals that participants set for themselves are quite easy to achieve to boost self-confidence, which encourages them to carry on. It should be remembered that the best way to change behaviour and maintaining this change is to "build on small successes".

continued on the next page

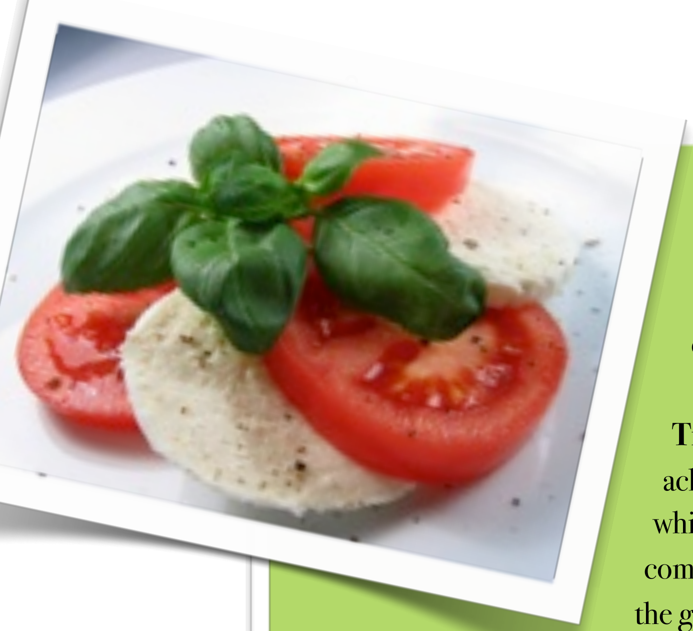

Relevant - Does the participant think that the goal is relevant? There needs to be clear link between the established goal, their health, and his/her overall goal.

Time bound – Is this goal the right thing to try to achieve at this moment in time? If so, set a time frame in which the goal can be achieved. If no target date for completion is set for completing a goal, trying to achieve the goal could go on (and on) without the client ever achieving it. If the next session is one week away, the goal completion should be aimed to be achieved within that week. If the goal requires a longer time-frame, it should be broken down into mini-goals that the client could achieve in time for the next session.

(*adapted from the Health Trainer Manual 2005, p.31-32*)

Health Trainer Manual (2005)

http://www.dh.gov.uk/en/Publicationsandstatistics/Publications/PublicationsPolicyAndGuidance/DH_085779

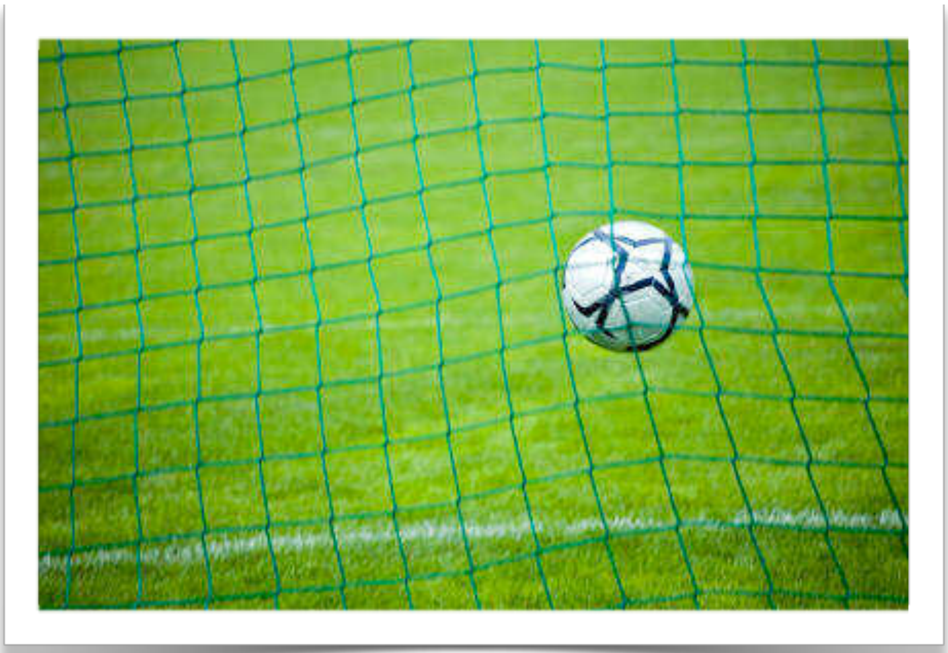

Planning is bringing the future into the present so that you can do something about it now

-Alan Lakein

See ppt. slide 4

How to perform Activity 2: Setting SMART Goals

"After analysing your self-monitoring pages, you've probably decided on at least one eating behaviour that you'd like to change or that you are currently not doing enough. For example you may have decided you'd like to decrease your consumption of sugary or fatty foods. At this point you need to set your first goal. Goals need to be SMART:

Specific
Measurable
Achievable
Relevant
Timely"

See ppt. slide 5

Specific – "Your goal needs to be clear and detailed, not vague. For exampl[illegible] vague goal would be 'eating healthily', whereas a clear, specific goal would be 'I will only eat 2 muffins a week, one when out for tea with the friends and the other on Sunday at lunch.' Ask yourself the following questions:

What am I going to do?
How am I going to do it?
When am I going to do it?
Where am I going to do it?

See ppt. slide 6

Measurable – "Making the goal specific means that it should be easy to measure. The example above, 'I will only eat 2 muffins a week, one when out for tea with the friends and the other on Sunday at lunch.' is measurable. You can record the number of times you eat muffins in one week, and also where, when and with whom. It would be hard to measure a vague goal like 'eating healthily'."

See ppt. slide 7

Achievable – "Try to set goals that are achievable for you. If you set yourself a really hard goal and don't achieve it, it can make you feel bad and you may want to give up. Make your first goal quite easy to achieve and this can give your self-confidence a boost."

See ppt. slide 8

Relevant – "Is this an important goal for you? Is it a behaviour that you really want to change? You are much more likely to succeed in reaching your goal if you can see the important difference that changing this behaviour will make to your health and your overall goal that you set last week."

See ppt. slide 9

Time bound – "Is this the right time to try to achieve this goal? Give yourself a set amount of time in which to complete your goal. If you don't give yourself a target date, it's easier to keep putting it off not starting to change your behaviour, and you may never reach your goal. Since our next session is next week, aim to have reached your goal in one week. If you think your goal will take longer than a week, try breaking it down into 'mini goals' so that you can achieve something that you specifically planned each week. For example, if your goal is to eat 5 portions of fruit and vegetables a day, a mini goal could be to eat at least 1 portion of fruit and vegetables each day."

See ppt. slide 10

"Now that we all know what SMART goals are I would like you to formulate your own SMART goal, which you would like to achieve over the next week." [*provide participants with the new weekly booklet and direct to the goal setting pages*]

(*adapted from the Health Trainer Manual 2005, p.31-32*)

Session 2

After explaining SMART goals, the participants attend to the goal setting pages in the booklet where they can write down not only their eating goal (what they want to do) but also to plan on how to put their goal into practice (how, when, and where they are going to do it).

The facilitator will need to stress that behaviour change is helped by a detailed plan of how they are going to change their behaviour. A plan includes questions on:

- **What** to do? E.g. "Eating more fruit and vegetables during the week"
- **How** to do it? E.g. "I will prepare a shopping list, there I will name my favourite 3 fruits and 2 vegetables, I will buy these. Each time I prepare a meal I will look into the refrigerator for vegetables that I can include in the meal and I will also look into the fruit that I have and either eat it as desert or save it for a mid afternoon snack". Or "If it is a weekday morning, then I will prepare a healthy lunch to take to work". This can be used to help the client remember to do their new behaviour. The situation becomes a trigger, or reminder to perform the behaviour. The idea is that eventually the behaviour will become automatic, a habit, so that, for example, the act of getting dressed for work in the morning will prompt you to prepare a healthy lunch.
- **When** to do it? "I will eat fruit at each meal: breakfast, lunch and dinner/tea, at lunch and dinner/tea I will eat at least one portion of vegetables"
- **Where** to do it? "Every time I am home for a main meal, or every time I go out for dinner/ tea; If at work I will pack some fruits and vegetables to take and have either as a snack or with my lunch; when at friends/family house I will make sure to also pack with me some fruit, just in case there is none available"

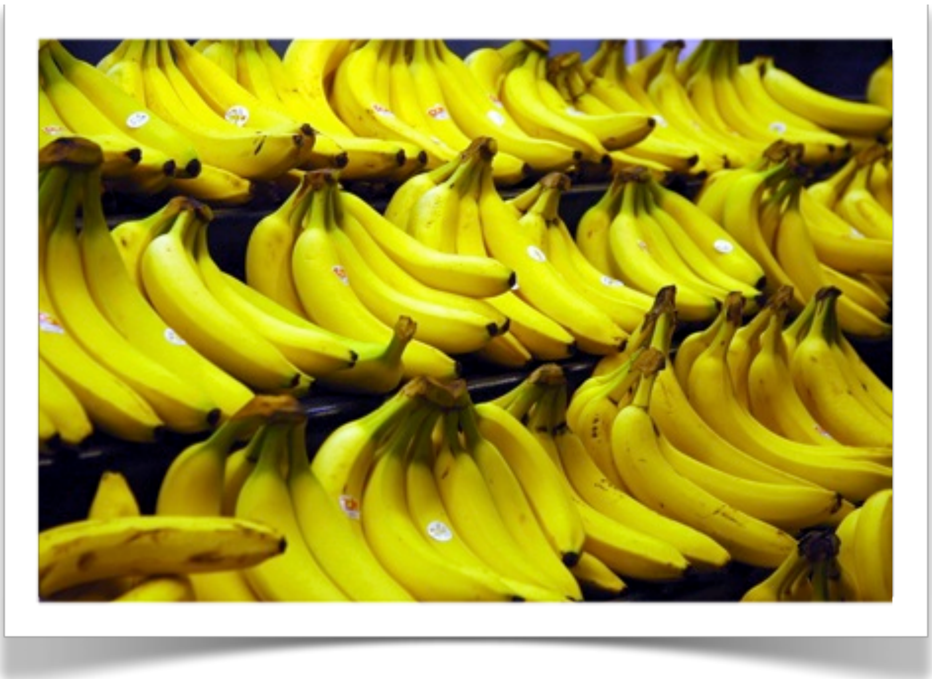

Activity 3: Identify and discuss eating barriers (15 minutes)

Technique description: What is a barrier identification?

People face many barriers when they try to change their behaviour, whether they want to or not. These barriers are often the main reason why people give up trying to change. The more barriers a person faces, the more likely he/she is to fall back into old habits.

However, once we know our barriers, we can come up with ways of overcoming these which makes it easier to change our behaviour. This is called barrier identification.

This activity will pave the way to activity 5 (formulating plans to deal with barriers and involve facilitators). It is essential that participants understand that in order to achieve goals, it is important to think what may make it difficult to achieve goals (the barriers). The facilitator will distribute the barrier worksheet for eating behaviour, this lists potential barriers to healthy eating. Participants will be asked to form pairs (*Note: every time a group task like this is presented in a manual the facilitator should take in consideration the limits of the context where the group sessions take place. If it is impossible to reorganise the room the facilitator should ask each of the members to work in pairs*). Each pair will read the list and discuss the barriers amongst each other keeping in mind questions such as "Would this barrier and solution apply to me?" and "Are there other potential solutions for this barrier that might work?". This can be done referring back to the eating diary.

Participants will also be provided with an additional list of potential barriers without any pre-specified solutions and should generate solutions for these (see other side of the worksheet). Following this, a discussion amongst the whole group will occur. During this discussion the facilitator should go over barriers and generate, collaboratively, possible viable solutions for these. For example, a participant wants to increase fruit and vegetable consumption, but is worried that the family, especially their picky kids, will not be supportive of this. However, if the participant manages to establish clear ground rules: "I will not prepare 2 different meals" or if the participant engages the whole family in the decision making process, deciding between different possible meal offers from a variety of recipes, and agreeing on a weekly menu, this will no longer constitute a barrier.

Situations that are likely to cause set-backs for the participant are also barriers. For example, a person trying to stop eating muffins might find it disturbing if the partner frequently stocks

the cupboards with these. The facilitator will ask the participants to think of any situations where the risk of a set-back would be high, and together think of some strategies for managing them. If the participant comes up with too many obstacles and does not engage in any of the problem solving suggestions presented by the facilitator and or members of the group the facilitator should notice the group rule (not taking up all of the time of the group) and state that other persons will be given time to speak and maybe in the end some solution will arise.

How to perform Activity 3: Identifying barriers

"Now that you all have written down a specific action plan, lets have a quick look at some of the things that might get in the way. As you can see on your goal setting form, there is also some space for writing down the things that might get in the way of your goals and ways of dealing with these. Everyone has times when it is difficult to do what they planned to do, so we need to be aware of these barriers and know exactly how we can deal with them."

"Next I would like you to to pair up and have a look at this barrier sheet [*facilitator distributes barrier sheet*], which lists potential barriers that people sometime face when changing their behaviour and ways of overcoming these."

See
ppt. slide 11

Examples of barriers

1. Unsupportive friends/relatives;
2. A picky family that does not want to embrace your life change;
3. Situations that make it especially difficult to perform the behaviour: e.g. attending a party
4. Feeling sad and depressed and wanting to fall back into old habits;
5. Not having time to prepare for the behaviour change;
6. Not having the time to embrace your goals.

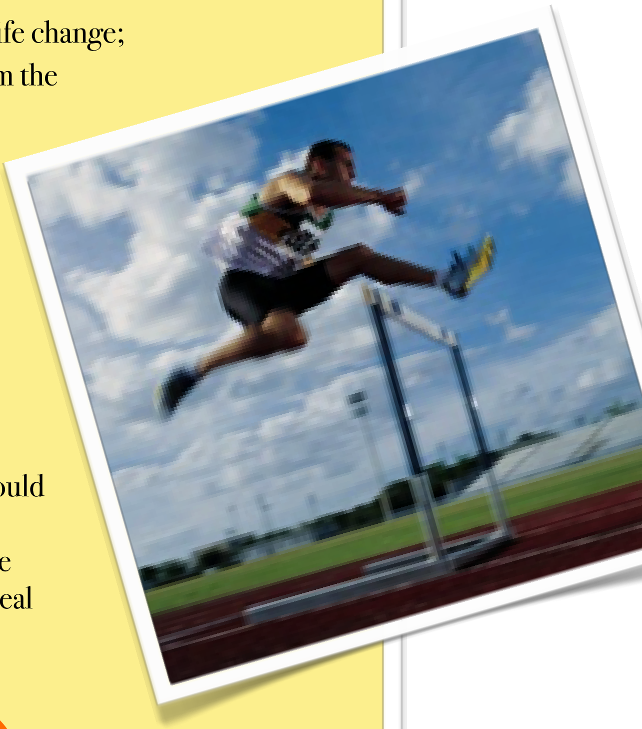

"I would like you to think about whether these barriers would also apply to yourself? After that we can have a group discussion about some other barriers, before you can write down in your booklet your own barrier and how you will deal with it"

See
ppt. slide 12

continued on next page

Some suggestions to overcome these barriers:

1. Explain your goals and request their open support making sure that they understand that this is a lifestyle change, and that it would be easier with their support.

2. If your family is picky and does not want to support your lifestyle change establish clear ground rules: "I will do___, even if you're not used to it". You can also involve them in the decision process of behaviour change. For example if the overall goal is to eat healthily, ask your family to select from a variety of healthy recipes that you can share with them, and establish a weekly menu agreed amongst the family.

3. If you are invited to a party where you know a lot of temptations to not stick to your goal will be present, make sure that you prepare yourself for these temptations before you leave the house. Tell yourself: "If this ___ is tempting me then I will do ___."

4. You feel sad and or/nervous and want to fall back into your old unhealthy habits? Then find yourself an alternative activity or a distraction. This way you will not be 'over indulging'.

5. If you feel you don't have the time to prepare for your behaviour change then think about the way you organise your day. Draw a clock on a blank page and divide this clock into the different things you have to do in a day (e.g. sleeping, personal hygiene, the kids etc.). After doing this look at the clock from a different perspective: what are your priorities? You will see that once you have established your priorities you will be better able to stick to them, and do less of the things that you don't really need.

6. Not having the time to embrace your goals is also related to time management. Organise your day so that your goals fit into your lifestyle.

Handy tip

Talking about goals is very handy because goals are nothing personal. They are only something the person wants to do. Goals have nothing to do with the personality or the identity of the person. Even if a person does not achieve a goal, it can be attributed to the goal being at fault rather than the person. This can be communicated to clients who tend to be hard on themselves.

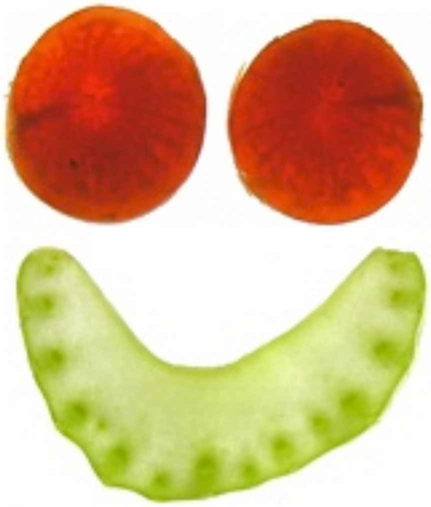

Activity 4: Identify and discuss eating facilitators (15 minutes)

Technique description: What are facilitators?

People can draw on facilitators which make changing their behaviour easier. These facilitators are often the main reason why people manage to maintain their behaviour change. The more facilitators a person encounters, the more likely he/she is to stick to the behavioural change and develop new habits.

How to perform Activity 4: Identifying facilitators

Examples of Facilitators

"Facilitators are for example people that encourage you/prompt you and/or support to act; Think of people who are likely to be supportive and encourage you to change, and think of ways to get the most benefit from these people by, for example, spending more time with them, having a specific person to phone when you need encouragement."

See ppt. slide 13

"The community can also be a great source of support for example, self-help cooking groups. Can you think of any useful sources of support that you could use?"

"Having access to local facilities such as a nearby supermarket (near home or work) that can supply you with the ingredients you need to prepare your meals; Reminders or situations that can trigger you to do the behaviour; e.g. placing your goal on the refrigerator door, as well as a list of foods to include, and avoid, in your plan. This will help you change your behaviour."

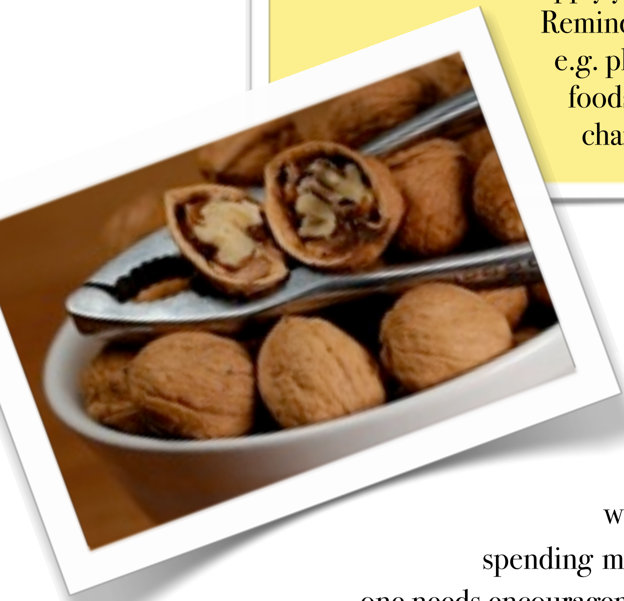

See ppt. slide 14

In order to continue preparing for activity 5 the facilitator will prompt a group discussion on what/whom could facilitate/support their goal. Participants should be encouraged to think of people who are likely to be supportive and encourage them to change, and think of ways to get the most benefit from these people, for example: spending more time with them, having a specific person to phone when one needs encouragement. The community can also be a source of support, such as self-help cooking groups for example. The facilitator should help participants to identify any useful sources of support and encourage their use. Participants will be asked to form pairs that look into the self-monitoring pages to identify facilitators. This is followed by a group discussion. During this group discussion the facilitator will summarise on the flip chart all the identified facilitators the group generated, this can serve as a pool of suggestions which each participant can use in the future to look for support.

Session 2

Activity 5: Formulate plans to deal with barriers and involve facilitators (15 minutes)

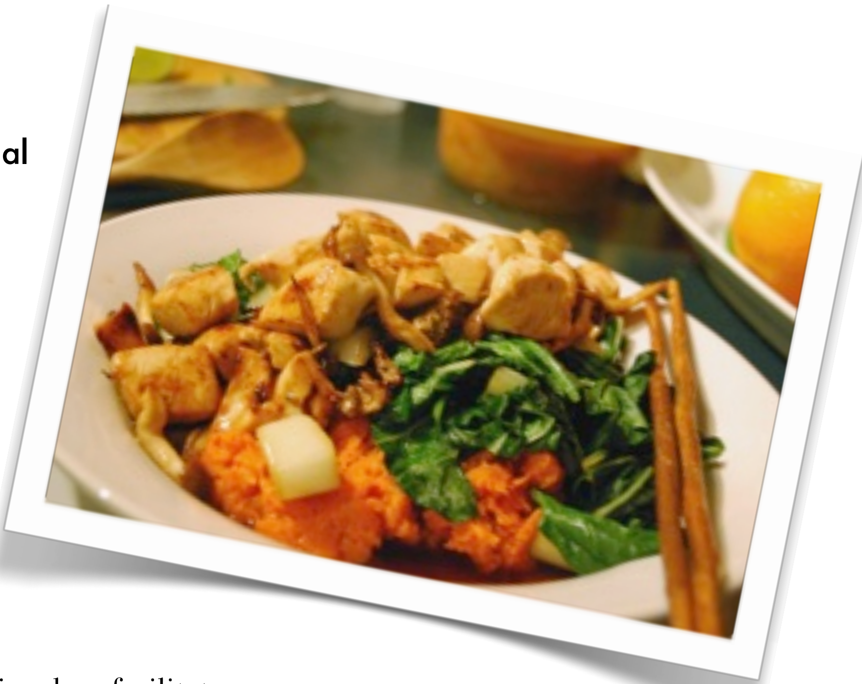

After the previous activities participants are aware of existing barriers and facilitators and possible ways to deal with these barriers and involve facilitators. At this moment participants form a so-called coping plan (Sniehotta et al., 2005) how to overcome potential barriers and involve facilitators throughout the forthcoming week (*facilitator will direct participants to the weekly booklet for this task*).

See ppt. slide 15

Each participant will be asked to look into their self-monitoring diary from the previous week and write down things, people, thoughts or feelings (barriers) that might make it difficult to stick to their goal and what could he/she do to overcome this, tackling any barrier in order to reach the goal, and what/whom could help to stick to their goal (how could I overcome this).

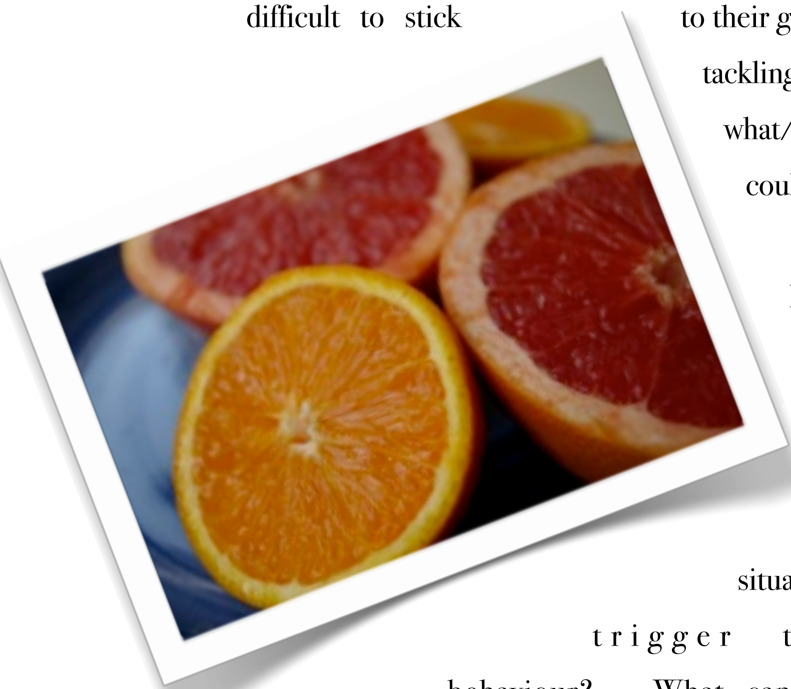

Participants should use their barrier pages in the weekly booklet to answer the question on "what/whom can make goal attainment easier?". Hence the participant should write "What actions, reminders or situations trigger this behaviour? What can the participant can do to increase these positive reminders? And how to get support from others for their behaviour change."

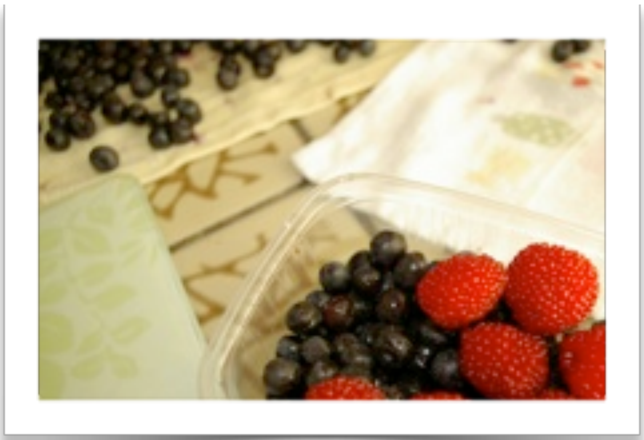

Activity 6: Close of session, summary and weekly challenges (10 minutes)

- "Being a detective": Self monitoring: participants will be self-monitoring their eating as well as their PA levels. For PA monitoring will also include noting step-counts using the pedometer.

See ppt. slide 16

How to perform Activity 6: Closing session

"So today we started off by reviewing last week. We talked about the British Heart Foundation booklet and discussed some of the questions that you had when reading it. We then looked at your self-monitoring records from last week and specifically focused on eating behaviour looking at what were the good things about what you ate and drank last week and also what the opportunities for change were, and the barriers that might get in the way of healthy eating. We then planned specifically what you would like to do next week, by setting a specific SMART eating goal. We made sure that this eating goal will be achieved by looking at some of the barriers that might get in the way, and how we could deal with these. I am certain that over the next week you will manage to achieve what you want to do. In any case, I am looking forward to seeing you again next week, whatever happens."

See ppt. slide 17

"And before you all take off, please don't forget to take your copy of this booklet which includes some very useful recipes. If you have any questions about this you can ask me next week." [*facilitator distributes the booklet "Food should be fun... and healthy"*]

"See you all next week."

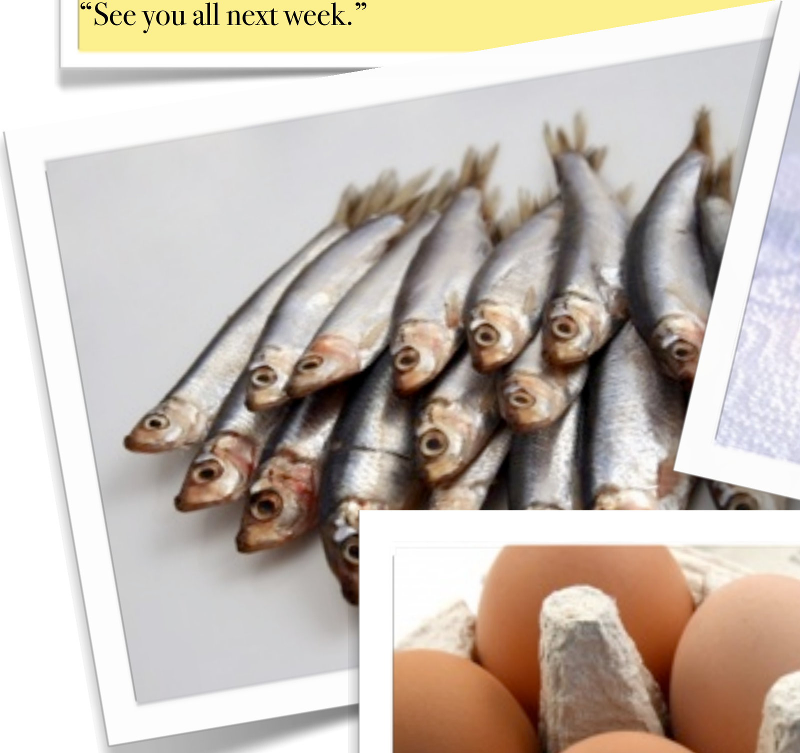

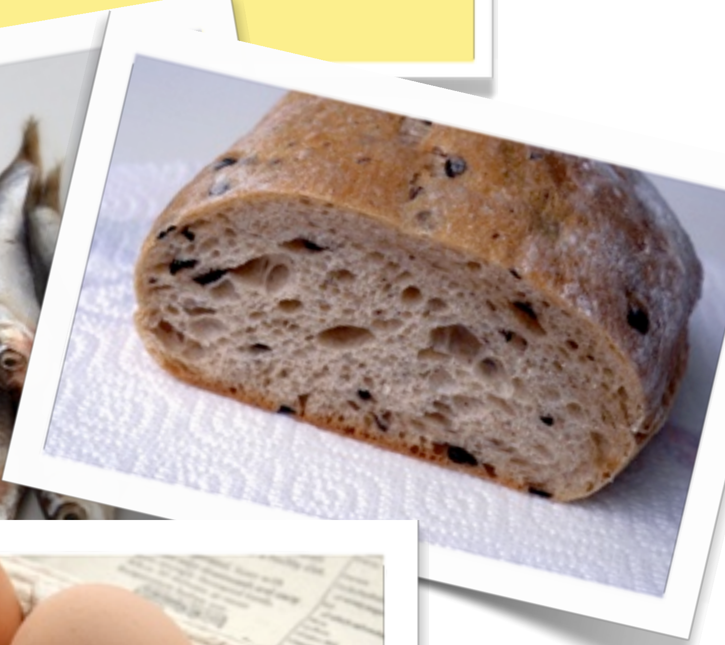

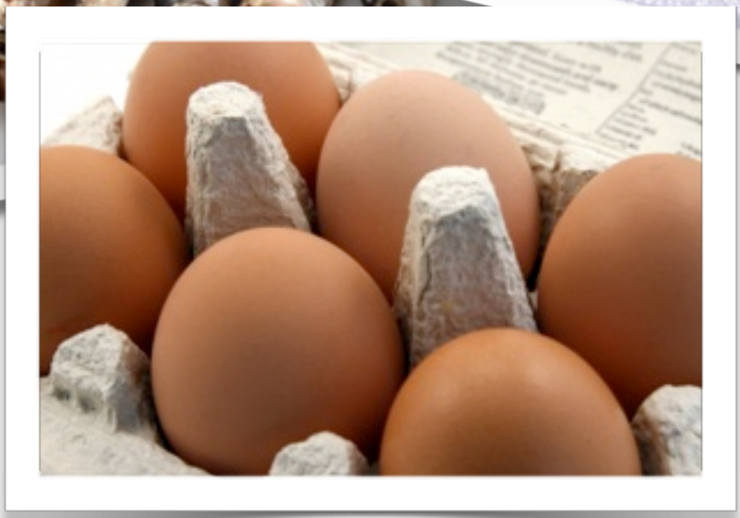

APPENDIX: SESSION SLIDES

Week 2

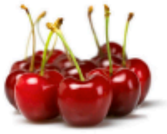

Goal Setting for Eating Behaviour

UNIVERSITY OF ABERDEEN · NHS Grampian · THE ROBERT GORDON UNIVERSITY

1

Today

1. Review of last week
2. Formulating an eating goal
3. Barriers
4. Facilitators
5. Protecting my eating goal: overcoming the barriers and involving the facilitators.

2

Review of last week

- How did you get on last week?
- Have a look at your self-monitoring sheet.
- Team up with your neighbour and share:
  1. Your "best bits"
  2. You "opportunities for change"
  3. What are the barriers to these opportunities?
- Report back to the group.

3

Formulating an eating goal

- After analysing your self-monitoring sheet:
  – decide on one eating behaviour you' d like to change.
  – set your first goal.
- Goals need to be SMART.
  – SMART stands for
    - Specific
    - Measurable
    - Achievable
    - Relevant
    - Timely

4

Specific

- Your goal needs to **be clear and detailed**, not vague.
- "Eating healthily" is vague.
- "I will eat only 2 muffins a week, one when out for tea with the friends and the other on Sunday at lunch." is specific.
- Ask yourself the following questions:
  – **What** am I going to do?
  – **When** am I going to do it?
  – **Where** am I going to do it?
  – **With whom** am I going to do it?

5

Measurable

- The goal should be **easy to measure**.
- "Eating healthily" is hard to measure.
- "I will eat 2 muffins a week, one when out for tea with the friends and the other on Sunday at lunch." is easy to measure.

6

Achievable

- Set goals that are **possible**.
- If you set a hard goal and don't achieve it, it can make you feel bad and you may want to give up.
- Make your first goal easy to achieve and this can give your self-confidence a boost.

Relevant

- Is this an **important** goal for you?
- Is it a behaviour that you really want to change?
- You will succeed in achieving your goal if you see the difference that changing this behaviour will make to your health and your overall goal that you set last week.

Timely

- Is this the **right time** to try to achieve this goal?
- Give yourself a set amount of time in which to achieve your goal.
- Aim to reach your goal in one week.
- If you think your goal will take longer than a week, break it down into 'mini goals'.
- For example, if your goal is to 'eat 5 portions of fruit and veg a day', a mini goal could be to 'eat at least 1 portion of fruit and veg each day'.

Formulating an eating goal

- Write down a goal in your new weekly booklet for this week (page 4).
- Be SMART about it.

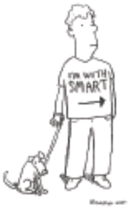

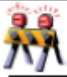

Barriers

1. Unsupportive **friends/relatives**;
2. A picky **family** that does not want to embrace your life change;
3. Unsupportive **situations** e.g. a party;
4. **Feeling** sad and depressed and wanting to eat comfort food;
5. Not having **time** to prepare food.

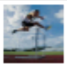

Barriers

- Have you come across these barriers before?
- How could you overcome these barriers?
- What other barriers could get in your way?
- How could you overcome these other barriers?

Facilitators

- **People** that encourage and support you;
- **The community** (e.g. self-help cooking groups);
- **Local facilities** (e.g. a nearby supermarket that can supply you with the ingredients you need to prepare your meals);
- **Reminders** or situations (e.g. placing your goal in the refrigerator, as well as a list of foods to include and avoid in your plan).

13

Facilitators

- How could you involve these facilitators?
- What other facilitators could help you?
- How could you involve these facilitators as well?

14

Achieving my eating goal

- Thinking about your potential barriers and facilitators formulate a plan:
  - How you could overcome barriers?
  - How could you involve facilitators?
- Use your booklet to note down your plan (weekly booklet page 5).

15

For next week

- Self-monitoring your eating and PA behaviour.
- Use your weekly booklet (Week 2).
- For eating: normal behavioural diary (p. 6-19). For PA: ABC diary (p.22-27)
- Bring your booklet and pedometer to the next session.

16

Facilitator: Source of recipes

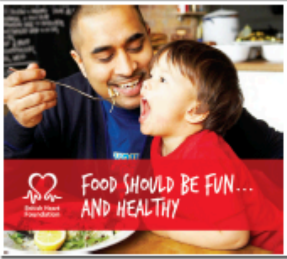

On your way out don't forget your copy of this leaflet for inspiration!

17

APPENDIX: BARRIER SHEET

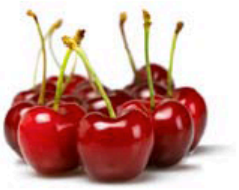

Typical EATING Barriers and Possible Solutions

Healthy Eating Barrier	One way of overcoming the barrier
Unsupportive friends/relatives.	Explain your goals and request their open support making sure that they understand that this is lifestyle change you have to make that would be easier with their support.
A picky family that does not want to embrace your life change.	Establish clear ground rules: "I will not prepare two different meals". If they are really hungry they will eat. You can also involve your family by asking them to select from a variety of healthy recipes and establish a weekly menu agreed amongst all members of the family.
Unsupportive situations, e.g. a party.	Make sure that you eat before you leave the house. Eating before going out could become part of your preparation to leave the house. Instead of only dressing for the party, you will also prepare and eat a meal that follows your goals and only then leave you the house. Give enough time for this to happen.
Feeling sad and depressed and wanting to eat comfort food.	If you feel sad and or/nervous and want to eat comfort food find yourself an alternative activity or a distraction. This way you will not be "over indulging", here are some examples:<br>· Do something that gives you pleasure - gardening, a warm bath, listening to music, reading a good book<br>· Try calling or visiting a friend that you know will encourage you in your goals.<br>· Think about how you feel and try to untangle the cause and find a better solution, if needed discuss this with your partner/friend/family.<br>· If you need more energy - go for a quick walk or do some exercise.<br>· Take up a hobby that you like: volunteer work; a craft work.
Not having time to prepare food.	Organise your day. Draw a clock in a blank page and divide this page into the different things you have to do in a day (including indispensable things such as time for sleeping and hygiene). After doing this look at this clock from a different perspective: what are my priorities? Once you establish your priorities you will be better able to stick to them eliminating from your day what you do not really need.

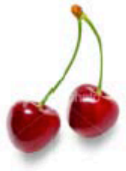

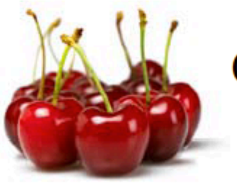

Classic Eating Barriers

Try and come up with good ways of dealing with these barriers

Healthy Eating Barrier	One way of overcoming the barrier
I don't like fruits and vegetables.	
I'm out of the habit of eating fruits and vegetables.	
I don't know how to serve fruits and vegetables to my family.	
I eat out a lot.	
Fresh produce spoils before I can eat it.	
Other snacks are more convenient.	
Fruits and vegetables cost too much.	
At the end of the day I am too tired to cook a healthy meal.	

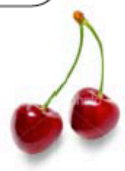

adapted from http://www.medicinenet.com

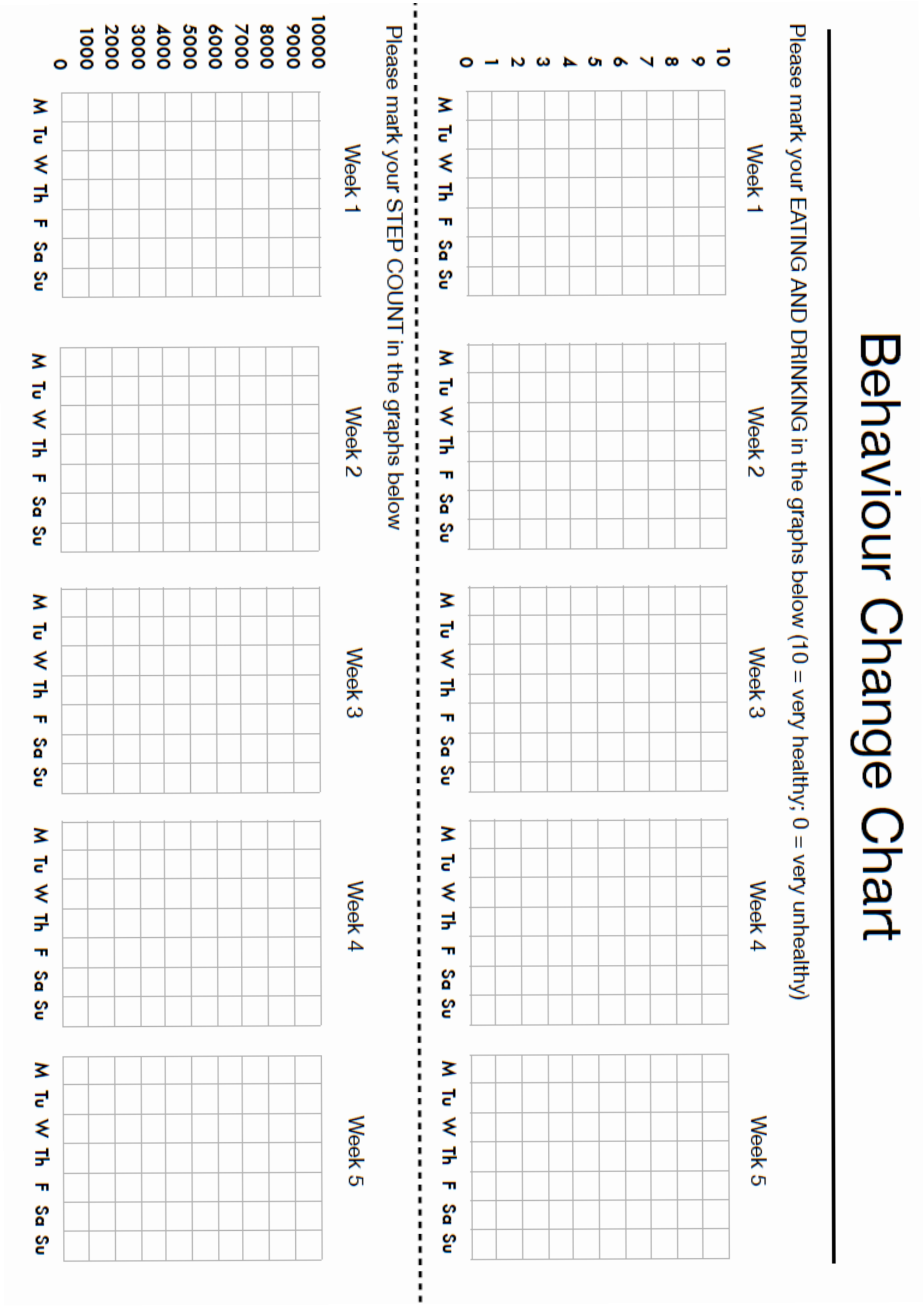
Behaviour Change Chart
Please mark your EATING AND DRINKING in the graphs below (10 = very healthy; 0 = very unhealthy)
Week 1
Week 2
Week 3
Week 4
Week 5
10
9
8
7
6
5
4
3
2
1
0
M Tu W Th F Sa Su
Please mark your STEP COUNT in the graphs below
10000
9000
8000
7000
6000
5000
4000
3000
2000
1000
0
