# Supplementary material for: Randomised Controlled Feasibility Trial of an Evidence-Informed Behavioural Intervention for Obese Adults with Additional Risk Factors
Source: PLoS One. 2011 Aug 29;6(8):e23040. doi: 10.1371/journal.pone.0023040 (PMC3163575; doi:10.1371/journal.pone.0023040)

The ABC Weight Loss Study -Session3-

Health Psychology Group
University of Aberdeen - August 2009

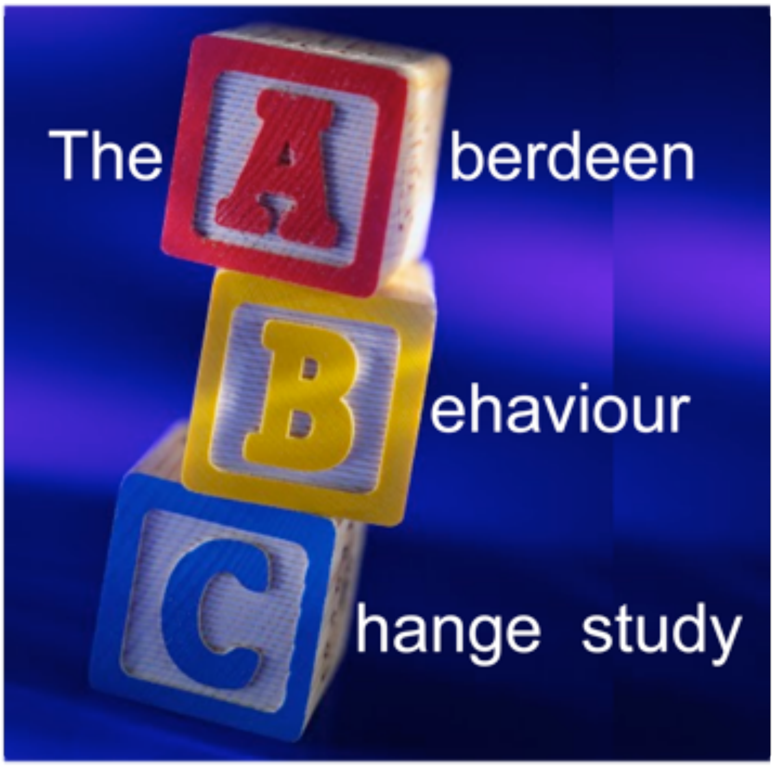

Authored by

Vera Araújo-Soares, Stephan Dombrowski & Falko Sniehotta

(in alphabetical order)

© University of Aberdeen

Table of Contents

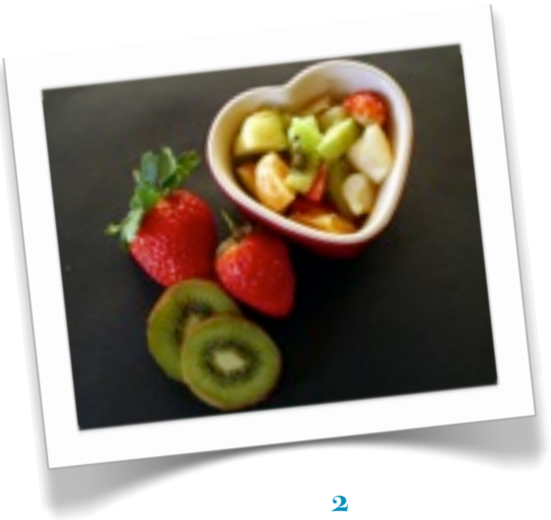

Overview 2

The Session Basics 2

Summary 2

Goals 2

Techniques 2

Materials 3

Recommended time 3

Before the third meeting 3

Activities and procedures 3

Content 4

Review of Eating, Introduction of Physical Activity 4

Activity 1: Review of last week (10 minutes) 4

Activity 2: Setting a new SMART eating goal (15 minutes) 6

Activity 3: Formulate a PA goal (15 minutes) 7

Activity 4: Identify and discuss barriers for PA (15 minutes) 9

Activity 5: Identify and discuss PA facilitators (15 minutes) 13

Activity 6: Formulate plans to deal with barriers and involve facilitators (15 minutes) 14

Activity 7: Weekly Challanges (5 minutes) 14

Appendix: Session Slides 15

Appendix: Barrier Sheet 18

Overview

Detailed overview of Session 3

THE SESSION BASICS

Summary

The third session aims to review dietary goal attainment from last week using the self-monitoring diaries (or recollection if diaries have been forgotten). Possible barriers that might have gotten in the way of achieving the goal will be identified and new dietary goals for the forthcoming week will be set taking into account possible barriers. In addition, the third session aims to introduce the importance of physical activity (PA) and encourages the setting of a specific PA goal and coping plans for the forthcoming week. In addition, participants will be asked to practice one PA behaviour over the next week, such as going for a walk, a swim, or to the gym. Participants will again be asked to self-monitor their behaviour.

Goals

1. Review whether established eating goals have been attained during the week.
2. Formulate a new goal and coping plan based on the achievements, barriers and facilitators of the previous week.
3. Formulate a first SMART goal for PA.
4. Identification and discussion of *barriers* to PA.
5. Identification and discussion of *facilitators* to PA.
6. Formulation of a coping plan for PA.

Techniques

- Review of behavioural goals *New*
- Action planning
- Coping planning
- Self-monitoring
- Prompt practice *New*

Materials

- Weekly booklet
- Barrier sheets
- PowerPoint
- British Heart Foundation leaflet (Physical Activity)
- Physical Activity information leaflet ("50+ activities" [local leaflet published by Aberdeen City Council])

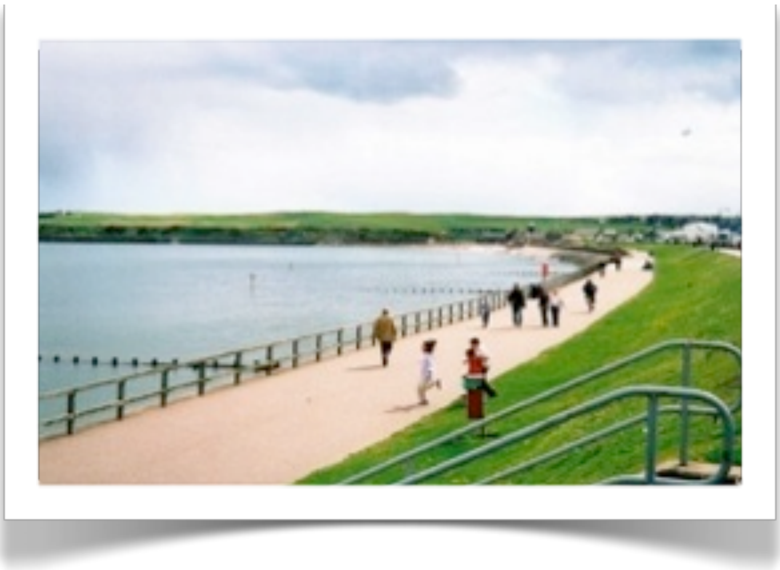

Recommended time

- 90 minutes

Before the third meeting

- Know how to introduce the third session;
- Have a clear idea about the structure of the first meeting;
- Have a clear understanding of the behaviour change techniques;
- Get materials ready (see materials section above).

Activities and procedures

In the remainder of this session description you will find a detailed guide to the activities you should facilitate in Session 3.

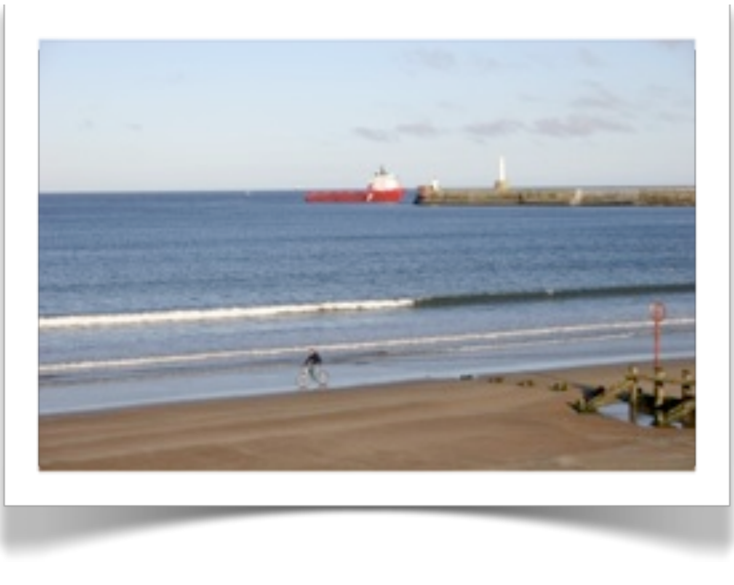

Content

Adding Physical Activity Plans: Slowly Changing my Life

REVIEW OF EATING, INTRODUCTION OF PHYSICAL ACTIVITY

See ppt. slide 2

Activity 1: Review of last week (10 minutes)

Following the overview of today's session, participants will review the goals set for eating behaviours during the last session using their self-monitoring pages. They will analyse how they managed to act on those goals. Participants will also be informed that eating and PA will be discussed sequentially with an initial focus on eating.

Technique description: What is reviewing behavioural goals?

Reviewing the goals that participants set for themselves is very important. It shows clearly how the intended behaviour change is going and helps participants to compare between what they planned to do with what they have done. This way it is possible to see what was achieved, what was partly achieved, or what was not achieved at all. This process usually leads to the formulation of new goals, keeping the previous experiences in mind. These new goals can be something completely different, the same goal, or a variation of the previous goal. Reviewing behavioural goals should be done in collaboration, it's not a way of controlling whether the participant has "been good". Participants should realise themselves how they have managed over the last week with the support of the facilitator. Facilitators selectively highlight any behaviour of the client that has been in line with the set goal.

Overall review of self-monitoring

The facilitator should start by asking participants if any problems occurred filling in the behaviour change diary over the previous week. If problems are reported these should be discussed and solved within the group context. Participants can share experiences of how they integrated self-monitoring in their daily life. The importance of self-monitoring in changing behaviour should be reiterated by the facilitator to increase motivation to continue monitoring.

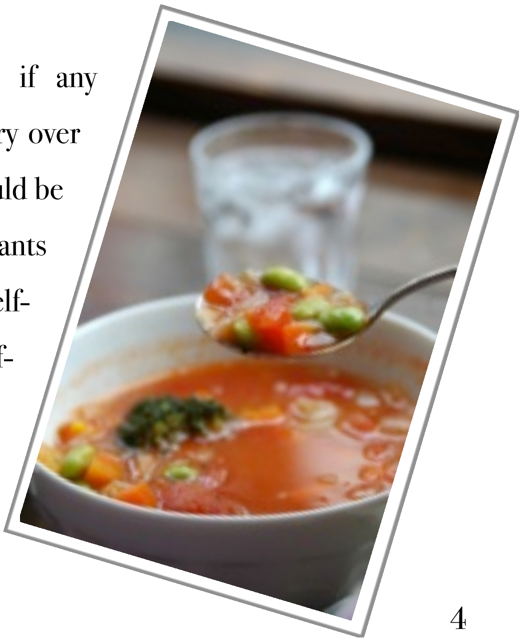

Session 3

Overall review of eating goal

In the group, participants will each in turn briefly review the attainment of their eating goal over the previous week. Participants will report on how many days they have managed to stick to their goal and any new and unforeseen barriers and facilitators they have encountered. The facilitator will help participants to assess if the goal established was helpful and if there is any need to redefine this goal or to break it down in smaller goals.

The facilitator should reinforce any efforts made to attain the behaviour goal and focus the group's attention on the positive feelings that emerged in the moment the goal was attained. It should also be stressed that even if the goal was only attained once, this still demonstrates that they are capable of achieving it. If the goal was not attained or only partially attained then it should be stressed that it is a problem of the goal that has been set, not of the person that set the goal.

Detailed analysis of achievements of the past week

After a first brief analysis participants will, in pairs, each in turn share in some detail what happened during the previous week, introducing their goal, their anticipated barriers and how they got on. The other person will reinforce any success (e.g. "well done"; "you have made it"). P articipants will be provided with a list of questions to reflect upon for guidance of this exercise .

- What did I try to do (what was my goal)?
- How much effort did I invest to achieve my goal?
- What impact/success did I have?
- What benefits have I experienced?
- What difficulties have I encountered?
- How did I manage these difficulties?
- What made it easier?
- Am I satisfied with what I did during last week?
- Am I getting enough support?
- Do I want to keep this goal or do I want to change it?

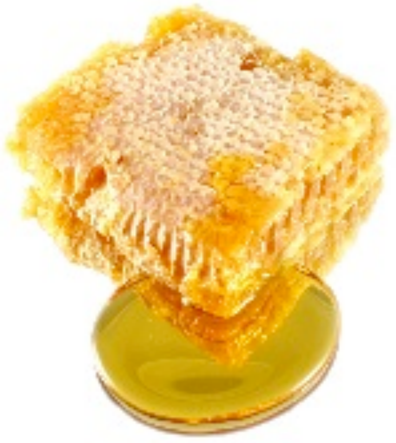

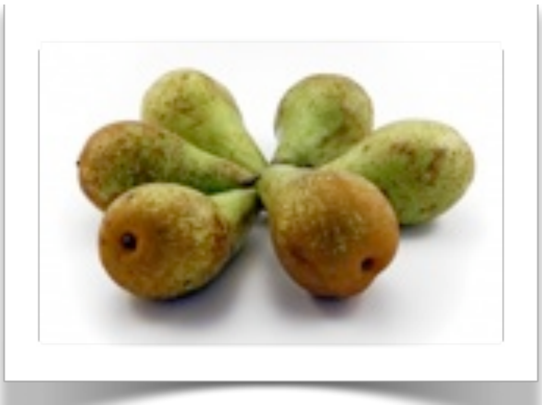

Handy tip

The provision of feedback provides the opportunity to stress the accomplishments and successes and allows the participants to obtain an outsider's perspective on the behaviour change efforts. This will keep the motivation high. It is important from early onwards to teach the client to self-identify success, to avoid becoming dependent on facilitator feedback. Another important feature could be to identify any social support in the client's environment that could provide this positive feedback

Three things to keep in mind for giving good feedback on performance:

1. **Descriptive rather than evaluative**: "You achieved your goal on four days last week" is more useful than "You are really good in changing your behaviour".
2. **Specific rather than general**: "Your physical activity [*used as an example*] seemed to be particularly high around Wednesdays and Fridays." Is more useful than "Great to see you increase activity at times."
3. **Focuses on the 'good bits' rather than the 'bad bits'**: "I think you did well managing to achieve your goal on Wednesday and Friday" is more useful rather than "You failed to achieve your goal on most days of the week".

Being descriptive, specific and positive in the feedback when giving feedback empowers to client to do more of the 'good stuff'.

How to perform Activity 1: Review of last week's eating goal

See ppt. slide 3

Overall review of self-monitoring

"How did you get on over the last week?
Did you manage to fill in your monitoring pages every day?"

If yes then praise anyone who has. If not or only partly then try and identify the barriers.

"What difficulties did you encounter filling in your booklet?
How could you overcome these difficulties?
Does anyone else have any suggestions of how you could overcome this difficulty? How did people manage to integrate the monitoring into their daily routine?"

Overall review of eating goal

"How did you get on with your eating goal last week? Have you managed to achieve the goals that you have set for yourself? What did it feel like on the day(s) when you achieved your goal? How many days did you manage to stick to your eating goal? Did you encounter any barriers and/or facilitators?"

Detailed analysis of achievements of the past week

"Please team up and have a look at your achievements from last week. Here are listed some questions which you can use to reflect on your achievements (facilitator shows slide 4 to the group)."

See ppt. slide 4 & 5

The pairs will then report back to the group focusing on: barriers; facilitators, successes and new goals. The facilitator will write on the flip chart the newly identified barriers (and facilitate discussion on possible answers). Participants will also be reminded to chart their healthy eating assessment and steps over the last week in the behaviour change chart. The facilitator will ask participants to note the behaviour change usually has ups and downs.

Activity 2: Setting a new SMART eating goal (15 minutes)

After the detailed analysis participants should set a new eating goal and plan how to overcome barriers and involve facilitators for the next week, keeping in mind what they have just analysed in the review of the past week. The facilitator should remind participants on how to set SMART

goals and formulate coping plans for achieving these goals. The self-monitoring pages from the previous week can be used as a basis for this exercise.

How to perform Activity 2: Setting a new eating goal and formulating a coping plan.

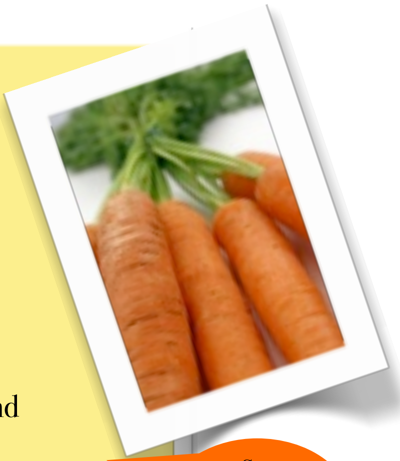

Setting of new eating goal

"Now that we all had a detailed look at the last week I would like to know:
- what barriers did you come across,
- were there any facilitators that you encountered,
- what could be your new eating goal for the next week and
- how could you deal with any potential barriers that might get in the way and involve possible facilitators?"

See ppt. slide 5

Activity 3: Formulate a PA goal (15 minutes)

Based on the assessment of the self-monitoring pages and the pedometer count of the past two weeks participants will decide on an "opportunity for change". Once they have decided what to change participants will again set a specific goal. The facilitator role will be to help the participant to set a goal that is detailed and likely to be achieved, again a SMART goal. Participants are familiar with this term by now. The facilitator will also distribute the "Get Active" information booklet outlining basic information on physical activity.

How to perform Activity 3: Setting a SMART PA goal

See ppt. slide 6

"After having recorded your PA and step count over the last two weeks, you've probably decided what you would like to change or increase. For example you may have decided you'd like to increase the number of steps that you do each day by 20% (e.g. you are walking an average of 1500 steps a day, in order to increase it by 20% you would need to increase your average by 300 more steps a day). At this point you need to set your first SMART PA goal. You remember what SMART stands for (**S**pecific, **M**easurable, **A**chievable, **R**elevant, **T**imely), but this time I am going to explain what this means in relation to PA."

See ppt. slide 7

Specific – Your goal needs to be clear and detailed, not vague. For example a vague goal would be "increasing fitness", whereas a clear, specific goal would be "I will walk to and from work 3 days a week" (a mini goal could be leaving two bus stations before reaching your work place 3 days a week). Ask yourself the following questions:
What am I going to do?
How am I going to do it?
When am I going to do it?
Where am I going to do it?
With whom am I going to do it?"

continued on next page

See ppt. slide 8

Measurable – "Making the goal specific means that it should be easy to measure. The example "I will walk to and from work 3 days a week" is measurable. You can record the number of times you actually managed to do this, and also where and with whom. It would be hard to measure a vague goal like "increasing your fitness".

See ppt. slide 9

Achievable – "Try to set goals that are achievable for you. If you set yourself a really hard goal and don't achieve it, that can make you feel bad, and you may want to give up. Make your first goal quite easy to achieve and this can give your self-confidence a boost.

For example the goal "I will walk to and from work 3 days a week" can be achievable for a person that lives relatively near work, but it can be very difficult to achieve for a person that lives miles from work. A more achievable goal might be "I want to increase my daily steps by 300", and instead of exiting at your regular bus stop you can stop two bus stops before your usual exit (either going from home to shopping, or from home to work and vice-versa)."

See ppt. slide 10

Relevant – Is this an important goal for you? Is this a behaviour that you really want to change? You are much more likely to succeed in reaching your goal if you can see the important difference that changing this behaviour will make to your health. As we have discussed during Session 1, a key factor in the getting and maintaining too much weight is the 'energy balance equation'. Changing our eating and drinking patterns will help us to lose weight, but only by increasing our activity levels, will we be able to maintain this weight loss over time. Besides, increased activity levels will also contribute to the initial weight loss and improve your health and overall well-being ."

See ppt. slide 11

Timely – Is this the right time to try to achieve this goal? Give yourself a set amount of time in which to complete your goal. If you don't give yourself a target date it's easier to keep putting off actually starting to change your behaviour, and you may never reach your goal. Since our next session is next week, aim to have reached your goal in one week. If you think your goal will take longer than a week, try breaking it down into 'mini goals' so that you can achieve something each week. For example, if your goal is "I will walk to and from work 3 days a week", a mini goal could be to "I will leave two bus stations before reaching my work place 3 days a week".

After revisiting the concept of SMART goal setting participants will be asked to write down their activity goal.

See ppt. slide 12

The facilitator will need to stress that behaviour change is helped by a detailed plan of how they are going to change their behaviour. A plan includes questions on:

What to do? E.g. "I will leave two bus stations before reaching my work place 3 days a week" or "I will increase my steps by 300 each day". It is important to emphasise that mild and moderate activities, in particular walking, are advisable for participants not experienced and trained with more rigourous activities.

When to do it? "Every Monday, Wednesday and Friday I will leave two bus stops before" or "Every day I will look at my step counter and see if I have managed to increase my steps by 300, if I have not, I will walk the dog, go for a walk with my partner or walk up and down the stairs in order to increase my step count". The idea is that eventually the behaviour will become a habit, so that, for example, the act of getting dressed for work on Monday, Wednesday or Friday morning will prompt you to prepare for a walk

Where to do it? "I can walk in the sidewalks, in my neighbourhood, in the garden, at the beach, in the countryside"

With whom (optional) to do it? "Either alone or with my dog, or my partner, or my family/friends".

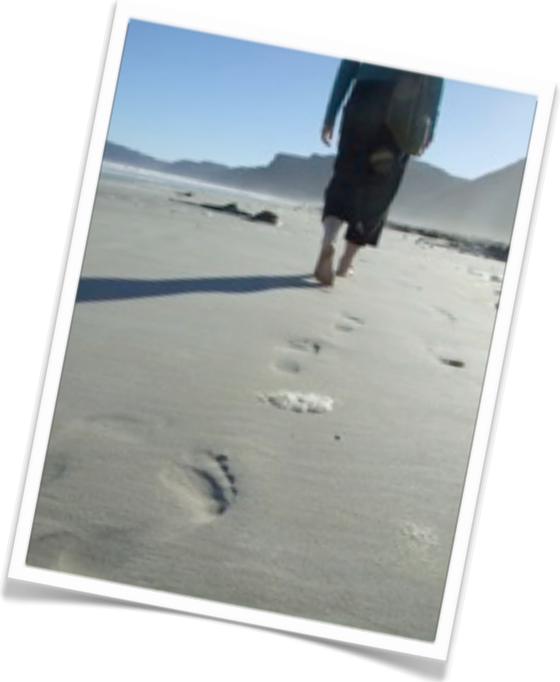

Activity 4: Identify and discuss barriers for PA (15 minutes)

This activity will pave the way to activity 5 (formulate coping plans by filling out the "Protecting my Activity Goal" working pages).

It is important that participants understand that in order to achieve goals, they have to reflect on what may make it difficult to achieve goals (the barriers). For this the facilitator will discuss with the participants a list of potential barriers to a more physically active life. The task of the group will be to analyse if these potential barriers reflect in any way what happens in their life. During this discussion the facilitator should discuss any barriers with the group and together come up with possible solutions. For example, a participant wants to increase his/her step count, but is worried about the rain. However, if the participant decides to go for this walk even if it is raining then he/she can use the adequate clothing and shoes to protect from the rain. Situations that are likely to cause set-backs for the participants are also barriers. For example, a person is trying to go for a walk and suddenly discovers an unannounced friend at the door. Ask the participants to think of any situations where the risk of a set-back would be high, and together think of some strategies for managing them.

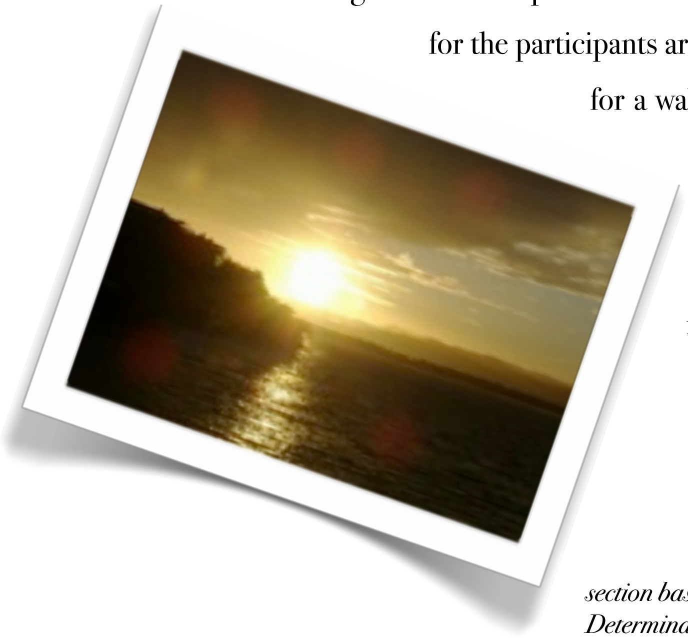

section based on Biddle, J.H. and Mutrie, N. (2001). Psychology of Physical activity: Determinants, well-being ad interventions. Routledge: London and New York

How to perform Activity 4: Identify and discuss barriers for PA

See
ppt. slide 13

facilitator distributes the physical activity barrier sheet

"Have a look at the example barriers and the solutions listed on this sheet."

Examples of barriers:

1. Unsupportive friends/relatives;
2. Situations that make it especially difficult to perform the behaviour: e.g. conflicting goals – a friend calls in when we are ready to go out for a walk; being surrounded by family/friends that are not committed to the same activity goals the participant as chosen form him/her self.
3. Feeling sad or stressed or depressed;
4. Not having time to do it;
5. Feeling embarrassed to go to the local gym;
6. Scottish weather

See
ppt. slide 14

"Have you come across these barriers? How could you overcome these barriers? Some additional suggestions for how you could overcome these barriers are also printed on the sheet."

Some suggestions to overcome these barriers:

1. Explain your goals and request their open support making sure that they understand that this is a life style change that you have decided to make and that it would be easier to fulfil with their support.
2. If a friend comes around at the same time you are leaving for a walk, or if that friend is calling you to invite you for tea/ a party there are some things you can do. Either you say that you cannot talk now, or invite him/her along. If you really want to attend to this party ask if it is possible for you to arrive later since you will start your walking… or, walk to the party (take fancy shoes in a bag, this way you can put them on once you arrive there!).
3. If you feel sad and or/nervous/stressed and feel like doing nothing do not forget that a walk will help you to settle your problems by allowing you some time for distraction. While you walk you will distant yourself from your troubles. This distance will allow you to reach different solutions.
4. If you feel you do not have the time to increase your activity please think about the way you organise your day around the clock and think about your priorities (we have talked about this last week, remember?). You will see that once you establish your priorities you will be better able to stick to them, eliminating from your day what you do not really need.
5. Feeling embarrassed will get you nowhere. If you know what you want follow your goals, no one can do it for you. Remember that everybody has problems and questions, and that even those that you believe have no problems (e.g. self image problems) also have them and need to fight against them too.
6. Prepare appropriate clothing.

" What other barriers could get in your way? How could you overcome these other barriers?

The facilitator will also discuss with the group some additional common barriers to engaging in a more active life.

"I'll look silly"
"I can't afford to go to a gym or pay a personal trainer"
"I'm too fat"
"I wouldn't know where to start"
"I wouldn't be any good at it"
"I have no energy to be active"
"I don't know how to exercise"
"I have no one to work out with"
"Got too old"

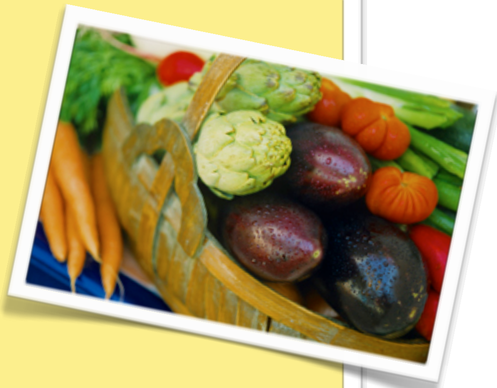

adapted from http://www.motivatingmates.com/info.php?sel=163

After presenting this the facilitator will say that these factors can be summed up in two tables (that s/he will share with the participants): one refers to opportunities to increase activity and another one presents barriers and possible solutions (adapted from Davis et al, 1995):

HOUR	Opportunity for activity/exercise	Reasons against/for
7:45	Walk the dog.	"I am late, so I can not take the dog out for a walk."
8:15	I take the bus to work.	"It is too far to walk, and my bicycle has a flat tire... "
10:00	Go shopping by car, when the market is so nearby.	"I could walk, but I did not want to say no to my friend who offered me a ride."
12:00	Lunch sitting on my desk.	"I want to save some time."
13:00	Calling different people who all work in the same building as me.	"It's more efficient to call. "
15:00	Walk to the post office.	"I need to stretch my legs."
17:00	Rest in my sofa at home.	"Instead I could go waking, but I am exhaust and not in shape."
19:30	At my sofa again	"I could go and walk my dog, or go for a walk with my partner, but is dark and it might not be safe. Also I have a headache. Maybe tomorrow."

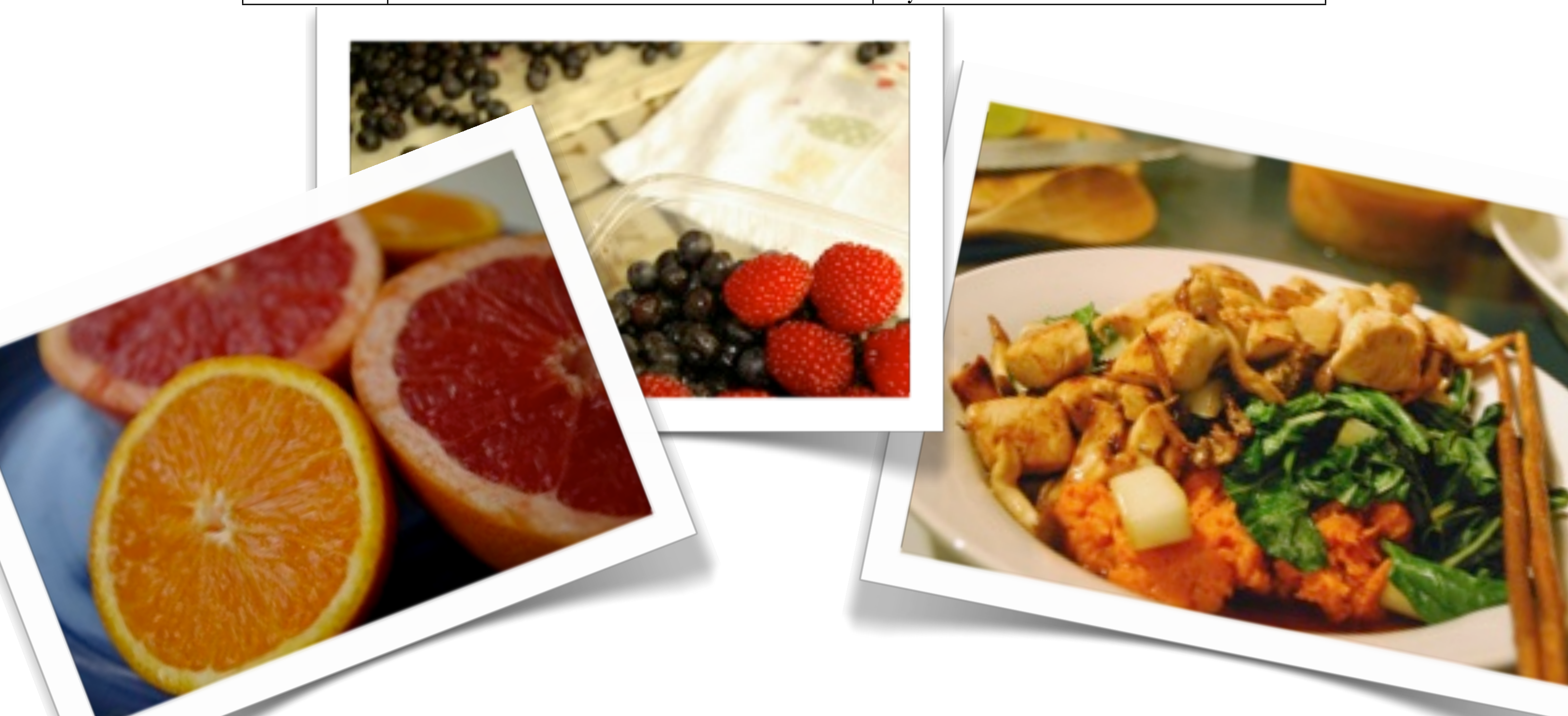

OPPORTUNITIES AND REASONS

Some alternative answers can be the following:

**Barriers/Reasons not to be active**	**Answer or solution/Achieving your lifestyle goals/Problem solving**
Delayed, I cannot walk the dog.	"I rarely have time to walk my dog in the morning since I awake too late. I will set my alarm clock to 15 minutes sooner, as soon at it rings I will stand up, I will not snooze, this way I'll have at least 15 minutes to walk the dog."
I cannot go with a bicycle ...I have a flat tire.	"It is not a question of "I can't": the truth is that I do not want to use the bicycle to go to work. But, I could probably repair it and use it sometimes during the weekend."
I cannot say NO to a friend that offers me a ride.	"I spend all my life saying "I can't". Of course I can say no but sometimes I decide not to. I will ask my friend to walk with me in the future."
Lunch sitting at my desk.	"Half an hour gives me sufficient time to walk for a while outside my working space (even if it rains as you can use a rain coat or umbrella that you can keep at your workplace)"
Not leaving my bus sooner since it looks like raining.	"This is the worst excuse ever! If I am worried with the weather I should dress for it and take an umbrella."
It is more efficient to call.	"It is true, but face to face contact is valuable. And I have time to speak in person with some of them."
I am too exhaust, heavy and unfit to do any activity.	"These are the signs of lack of activity, so these should be the true reasons that bring me to increase my activity."
My neighborhood is not safe after dark.	"I can ask my partner/friend/child to go with me, or try to walk in the morning hours, or use the time at lunch to go for a small walk, or I can go to a local gym."
I have a headache.	"Probably a sign of stress: doing some activity would do me good."
Maybe tomorrow...	"This is my favourite strategy to avoid activity! I will go right now to walk the dog... and myself!"

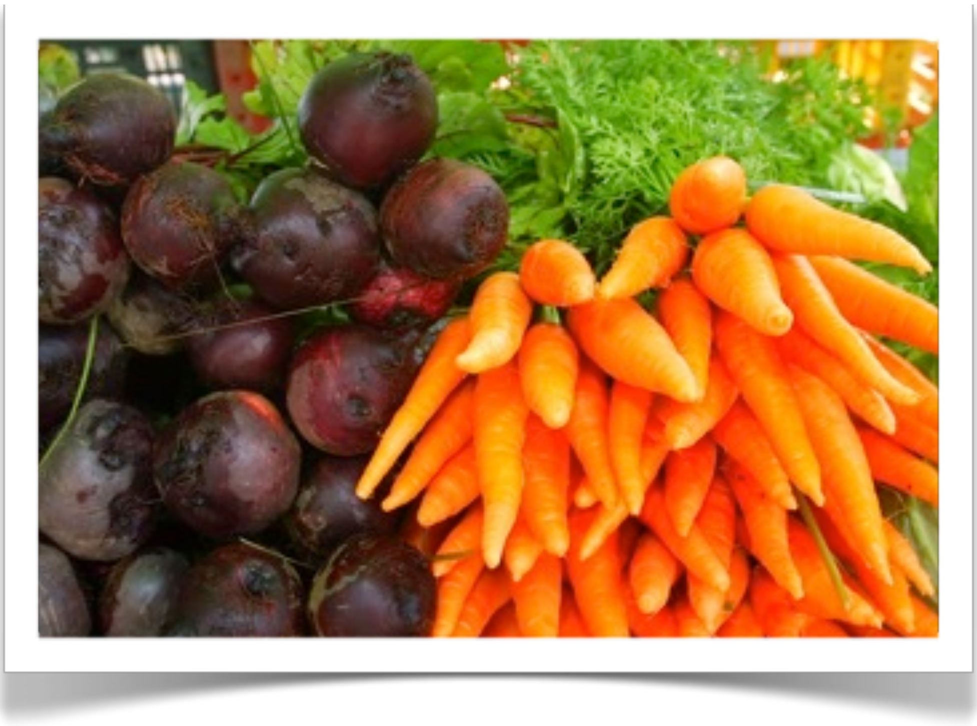

Activity 5: Identify and discuss PA facilitators (15 minutes)

In order to continue preparation for activity 5 the facilitator will prompt a group discussion on what/whom could facilitate/support their goal. This group discussion will be prompted by sharing the information in the following text box with the group.

See ppt. slide 15

How to perform Activity 5: Identifying and discussing PA facilitators

"Reminders or situations that can trigger you to do the behaviour; e.g. placing your most comfortable walking shoes at the door/hall; preparing some comfortable clothes to wear for the morning so that as you awake you remember to walk the dog. Placing a statement of your active goal on the bedroom door. This will help you to attain your behaviour.

Having access to local facilities such as a nearby gym or a local sports center can also help. Identifying a park that can be used for some walks can also be useful to increase PA levels;

The community can also be a source of support and offer some walking activities/other physical activities that they could participate in, etc. The group facilitator should help the participant to identify any useful sources (e.g. a local sports center) of support and encourage him/her to use them.

People that encourage you/prompt you and/or support you to act; The participants should be encouraged to think of people who are likely to be supportive and encourage them to increase their activity levels, and to think of ways to get the most benefit from these people. For example, spending more time with them, having a specific person to phone when they need encouragement."

See ppt. slide 16

(facilitator distributes physical activity information booklet "50+activities" explaining that this booklet is the only printed information available and gives good ideas for possible contacts, even if younger than 50).

Participants should be encouraged to think of situations that can trigger their desired PA behaviour. Participants should also be encouraged to think of local facilities in which or with which to undertake their PA activity (e.g. the gym or park). The community can also be a source of support. Finally, they can think of people who are likely to be supportive and encourage them to change, and think of ways to get the most benefit from these people. For example, spending more time with them, having a specific person to phone when they need encouragement. The facilitator should help the group to identify any useful sources of support and encourage them to use them. Participants will be asked to form groups (2 members each- if needed the facilitator will allocate participants to group by attributing a number-1,2 / activity - walk/brisk walk). The task of the pair will be to look into the booklets filled out during the last 2 sessions and to identify what facilitator is usually associated with being more active. They will have 5 minutes to discuss these facilitators. After this, a discussion amongst the whole group will occur. During this discussion the facilitator will summarise in the flip chart the facilitators that the group has identified. This list is then presented as a pool of suggestions that each participant can use in the future to look to for support.

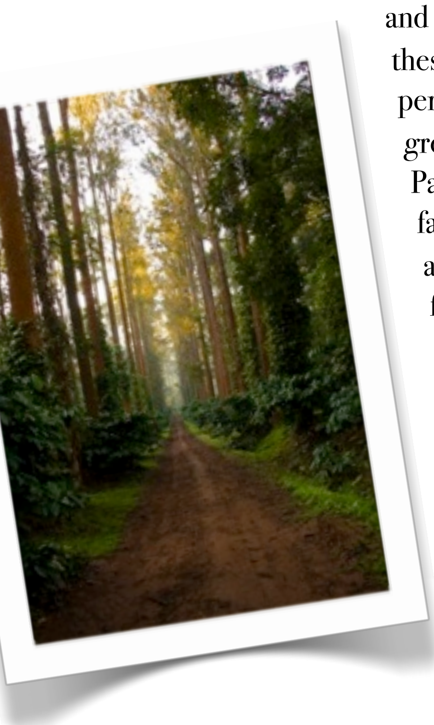

Activity 6: Formulate plans to deal with barriers and involve facilitators (15 minutes)

See ppt. slide 17

After the previous activities, participants should be more aware of existing barriers and feel comfortable and prepared to use strategies to deal with these. At this moment they will be ready to fill out the goal setting working pages in the booklet.

Each participant will be asked to write down the things, situations, people, thoughts or feelings that might make it difficult to stick to their goal (Barriers) and what he/she could do to overcome this: overcoming any barrier in order to reach the goal, and who could help them to stick to their own goal (How could I overcome this?).

They will also be instructed to use their monitoring pages to answer the question on "what/ who can make their goal attainment easier?". Hence the participant should write "What actions, reminders or situations facilitate this behaviour?", "What can be done to increase these positive reminders?", and "How to get support from others for their behaviour change?"

Activity 7: Weekly Challanges (5 minutes)

- Self-monitoring of behaviour change: each participant will be recording each day what they have been eating and drinking as well as their levels of physical activity. In this record they will also register if they have, or have not achieved their "Eating goal" and "Activity goal" and if not, the reason why. They will continue using the pedometer.

See ppt. slide 18

- Participants should be invited to think of and attend to pleasurable things or situations in preparation for the self-reward task in Session 4 "*Over the next week try to think and be attentive to things that you like and that you enjoy. You might want to note these down in your weekly booklet.*"

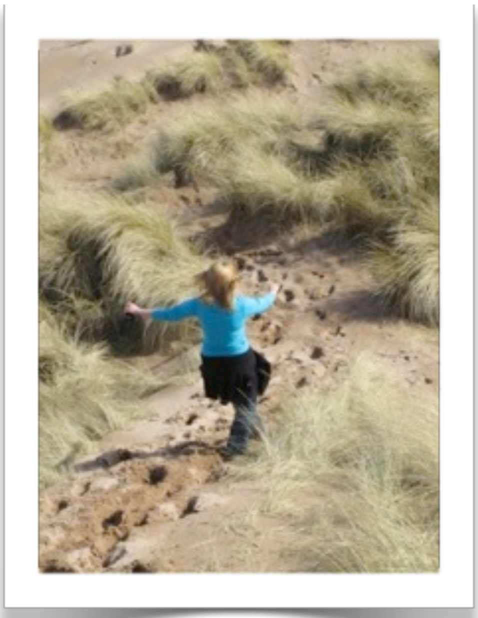

APPENDIX: SESSION SLIDES

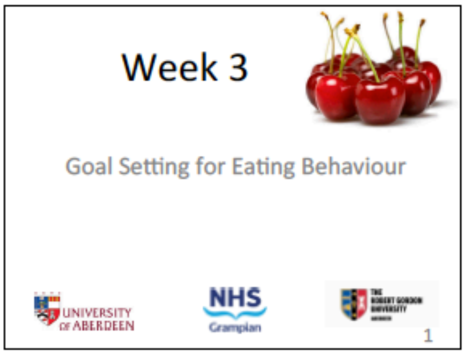

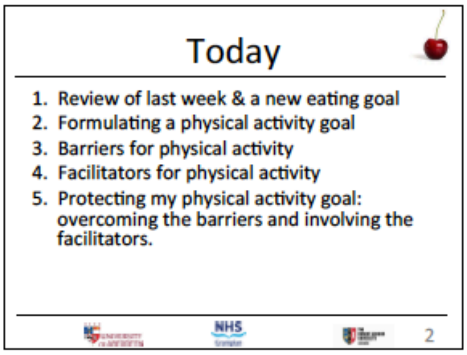

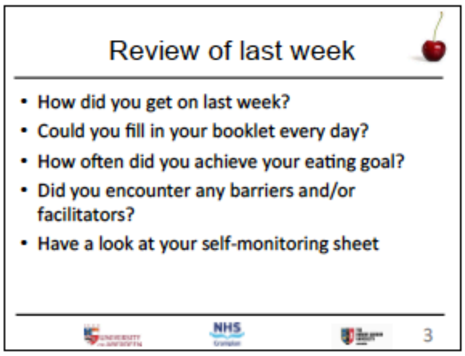

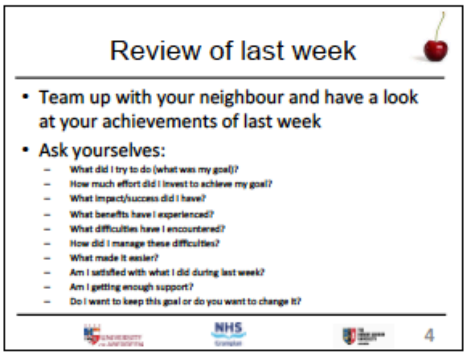

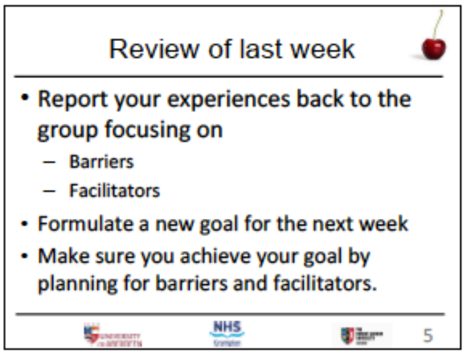

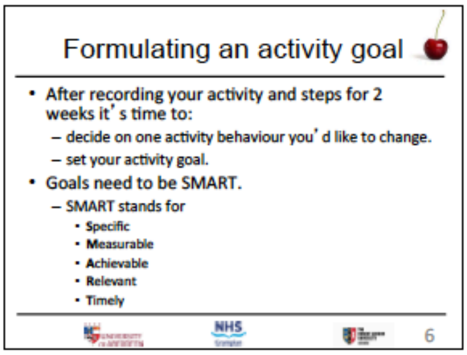

Specific

- Your goal needs to **be clear and detailed**, not vague.
- "Increasing fitness" is vague.
- "I will walk to and from work 3 days a week" is specific.
- Ask yourself the following questions:
  - **What** am I going to do?
  - **How** am I going to do it?
  - **When** am I going to do it?
  - **Where** am I going to do it?
  - **With whom** am I going to do it?

7

Measurable

- The goal should be **easy to measure**.
- "Increasing fitness" is hard to measure.
- "I will walk to and from work 3 days a week" is easy to measure.

8

Achievable

- Set goals that are **possible**.
- If you set yourself a hard goal and don't achieve it, it can make you feel bad and you may want to give up.
- Make your first goal easy to achieve and this can give your self-confidence a boost.

9

Relevant

- Is this an **important** goal for you?
- Is it a behaviour that you really want to change?
- You will succeed in achieving your goal if you see the difference that changing this behaviour will make to your health and your overall goal that you set in the first week.

10

Timely

- Is this the **right time** to try to achieve this goal?
- Give yourself a set amount of time in which to achieve your goal.
- The next session is next week, so aim to reach your goal in one week.
- If you think your goal will take longer than a week, try breaking it down into 'mini goals'.
- For example, if your goal is to eat 5 portions of fruit and veg a day, a mini goal could be to eat at least 1 portion of fruit and veg each day.

11

Formulating an activity goal

- Write down a goal in your new weekly booklets for week!
- Be SMART about it!

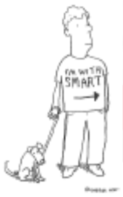

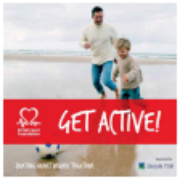

12

•

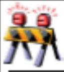

Barriers

1. Unsupportive **friends/relatives**;
2. A picky **family** that does not want to embrace your life change;
3. Unsupportive **situations** e.g. a party;
4. **Feeling** sad and depressed;
5. Not having **time** to be active.

13

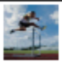

Barriers

- Have you come across these barriers before?
- How could you overcome these barriers?
- What other barriers could get in your way?
- How could you overcome these other barriers?

14

Facilitators

- **Reminders** or situations (e.g. placing your most comfortable walking shoes at the door/hall, preparing some comfortable clothes to wear for the morning);
- **Local facilities** (e.g. a nearby gym or local sports centre);
- **The community** (e.g. walking groups);
- **People** that encourage and support you.

15

Facilitators

- How could you involve these facilitators?
- What other facilitators could help you?
- How could you involve these facilitators as well?

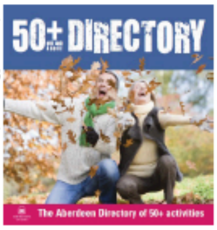

16

Achieving my activity goal

- Thinking about your potential barriers and facilitators formulate a plan:
  - How you could overcome barriers?
  - How could you involve facilitators?
- Use your booklet to note down your plan (weekly booklet page 5).

17

APPENDIX: BARRIER SHEET

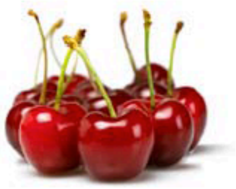

Typical ACTIVITY Barriers and Possible Solutions

Physical Activity Barrier	One way of overcoming the barrier
Unsupportive friends/relatives.	Explain your goals and request their open support making sure that they understand that this is a lifestyle change that you have decided to make and that it would be easier to fulfill with their support.
Unsupportive situations, e.g. a conflicting goals.	If a friend comes around at the same time you are leaving for a walk, or if that friend is calling you to invite you for tea or a party there are some things you can do. Either you say that you cannot talk now, or invite him/her along. If you really want to attend this party ask if it is possible for you to arrive a little bit later since you will start your walking... or, walk to the party (take your fancy shoes in a bag, this way you can put them on once you arrive there!).
Feeling sad and depressed and wanting to eat comfort food.	If you feel sad and or/nervous/stressed and feel like doing nothing do not forget that a walk will help you to settle your problems by allowing you some time for distraction. While you walk you will distance yourself from your troubles. This distance will allow you to reach different solutions.
Not having time to do it.	If you feel you do not have the time to increase your activity please think about the way you organise your day and think about your priorities (we have talked about this last week, remember?). You will see that once you establish your priorities you will be better able to stick to them eliminating from your day what you do not really need.
Feeling embarrassed to go to the local gym.	Feeling embarrassed will get you nowhere. If you know what you want then follow your goals, no one can do it for you. Remember that everybody has problems and questions, and that even those that you believe have no problems also have them and need to overcome them.

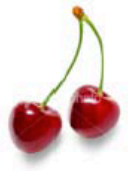

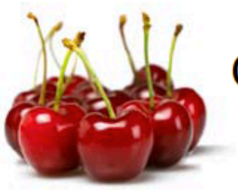

Classic Activity Barriers

Try and come up with good ways of dealing with these barriers

Activity Barrier	One way of overcoming the barrier
I'll look silly.	
I can't afford to go to a gym or pay a personal trainer.	
I'm too heavy.	
I wouldn't know where to start.	
I wouldn't be any good at it.	
I have no energy to be active.	
I don't know how to exercise.	
I have no one to work out with.	

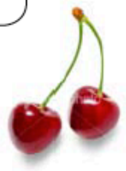

Supplement: Intervention Manual S3 — Intervention Manual Session 3. (PDF) [file pone.0023040.s013.pdf]
