# Supplementary material for: Randomised Controlled Feasibility Trial of an Evidence-Informed Behavioural Intervention for Obese Adults with Additional Risk Factors
Source: PLoS One. 2011 Aug 29;6(8):e23040. doi: 10.1371/journal.pone.0023040 (PMC3163575; doi:10.1371/journal.pone.0023040)
Supplement: Intervention Manual S4 — Intervention Manual Session 4. (PDF) [file pone.0023040.s014.pdf]

Session 4

The ABC Weight Loss Study

The Aberdeen Behaviour Change Study

Health Psychology Group
University of Aberdeen - August 2009

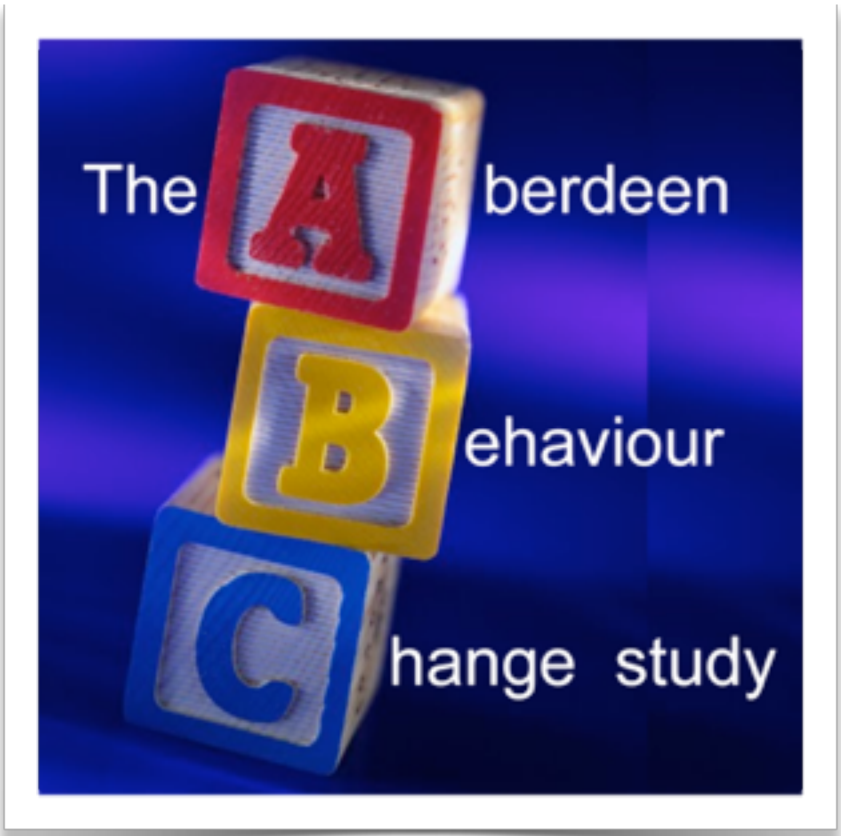

Authored by

Vera Araújo-Soares, Stephan Dombrowski & Falko Sniehotta

(in alphabetical order)

1495 UNIVERSITY OF ABERDEEN

ABERDEEN Health Ψ

THE ROBERT GORDON UNIVERSITY ABERDEEN

© University of Aberdeen

Session 4

Table of Contents

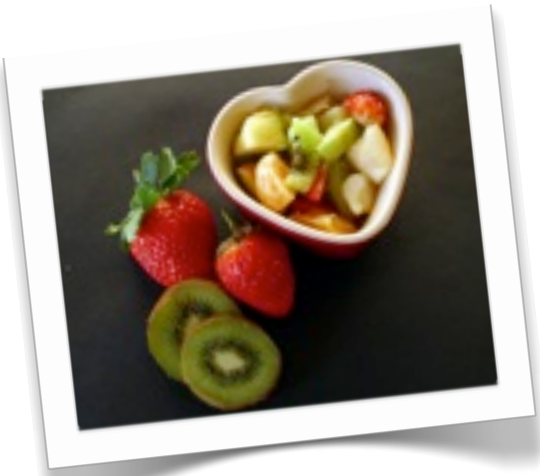

Overview 2

The Session Basics 2

Summary 2

Goals 2

Techniques 2

Materials 2

Recommended time 3

Before the fourth meeting 3

Activities and procedures 3

Content 4

Review of Eating and PA Goals and Planning Rewards 4

Activity 1: Review of last week (40 minutes) 4

Activity 2: Plan contingent rewards (40 minutes) 6

Activity 3: Weekly challenges (10 minutes) 8

Appendix: Slides 9

Appendix: Self-Rewards Sheet 11

Overview

Detailed overview of session 4 content

THE SESSION BASICS

Summary

The purpose of the fourth session is to review behavioural goals for both dietary and physical activity (PA) behaviours and to formulate new plans. Furthermore, this session also focuses on planning rewards for achievements and how to adjust goals and strategies over time to achieve sustainable behaviour change. Participants will again be asked to self-monitor their behaviour.

Goals

1. Review whether established eating and PA goals have been attained during the week and formulate goals and coping plans based on the achievements, barriers and facilitators of the previous week.
2. Plan contingent rewards for attaining eating and PA goals.

Techniques

- Review of behavioural goals
- Action planning
- Coping planning
- Self-monitoring
- Prompt practice
- Plan contingent rewards *New*

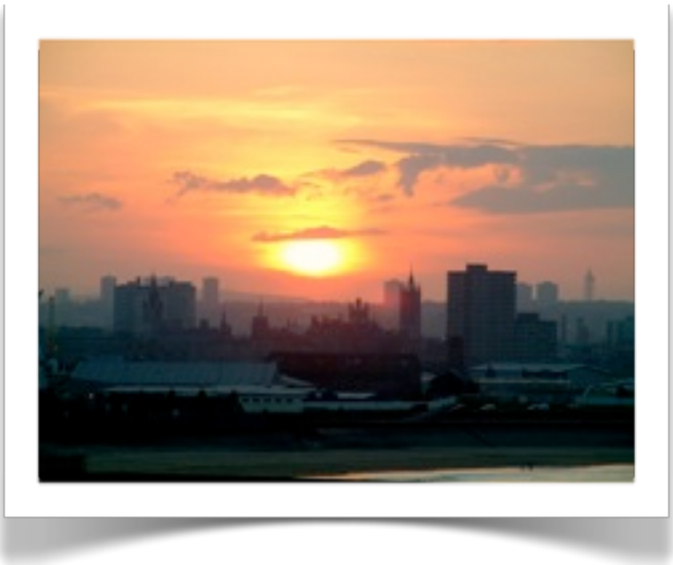

Materials

- Weekly booklet
- PowePoint Slides
- Flip-chart
- Worksheet self-rewards

Session 4

- Laminated example cards for rewards

Recommended time

- 90 minutes

Before the fourth meeting

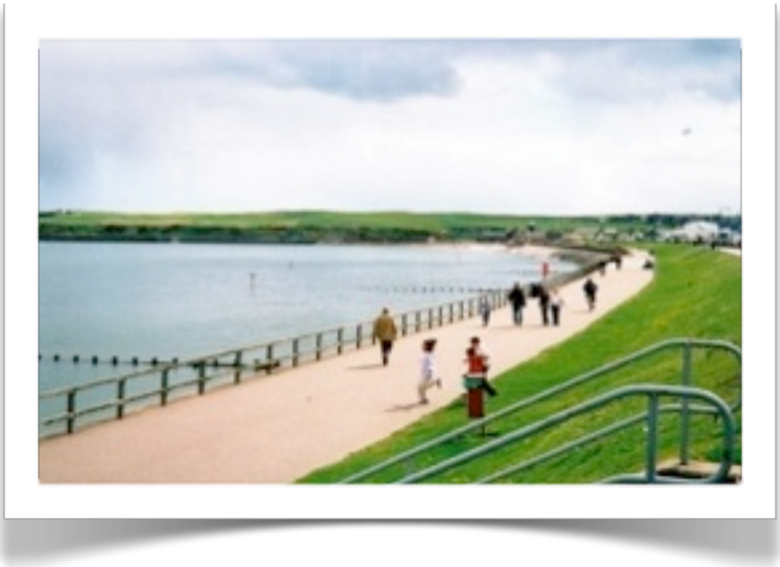

- Know how to introduce your role and the overall session structure;
- Have a clear idea about the structure of the fourth meeting;
- Have a clear understanding of the behaviour change techniques;
- Get materials ready (see materials section above).

Activities and procedures

In the remainder of this session description you will find a detailed guide to the activities you should facilitate in Session 4.

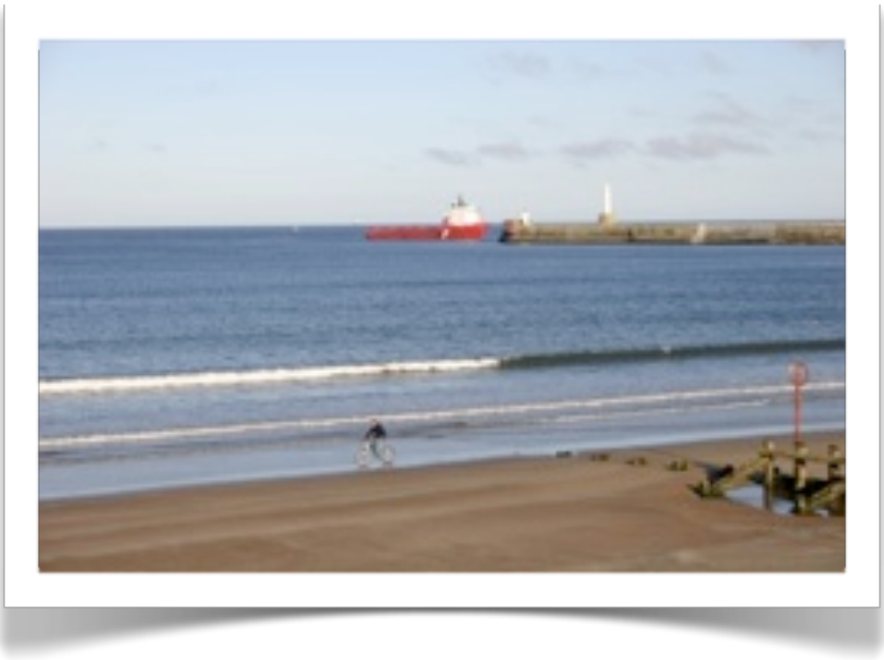

Content

Paving the way for the future

REVIEW OF EATING AND PA GOALS AND PLANNING REWARDS

See ppt. slide 2

Activity 1: Review of last week (40 minutes)

After introducing the session structure participants will reconsider the goals set for eating behaviours and PA during the last session. Participants will analyse how they managed to act on those goals by reviewing the self-monitoring pages. The review will start with last week's eating goals and will be followed by the PA/walking goals. The procedures will be similar to those used during session 3.

Participants will review if their eating and PA goal from the previous week were achieved and on how many occasions (by reviewing the self-monitoring pages). Barriers and facilitators encountered during the week will be analysed. If needed, new ways of overcoming barriers and involving facilitators will be generated. Participants will assess whether last week's goals was a SMART goal, if there is any need to redefine this goal, or to break it down into smaller goals. If the person has been successful in enacting the goal he/she should establish a new goal using the same steps as in the previous session, finding new "opportunities for change".

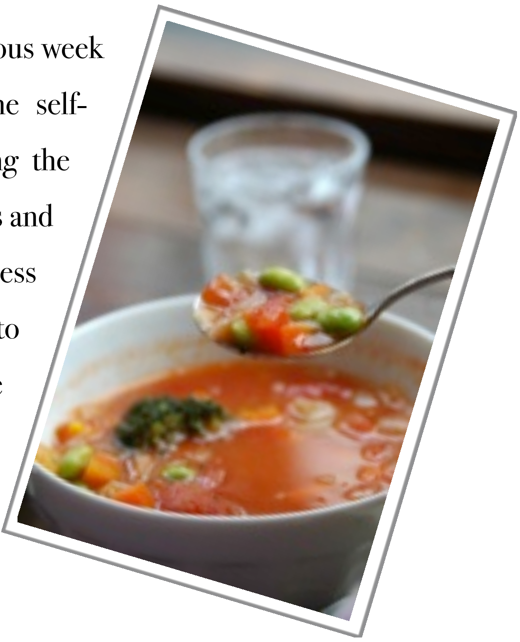

The facilitator should reinforce any efforts made towards attaining the goal and prompt the group to focus back on how it felt when the goal was attained (using questions such as "When you managed to achieve your goal, how did you feel?"). Even if participants only managed to attain the goal some of the time it should still be stressed that they are capable of achieving their goals. Participants should be encouraged to be more attentive to the barriers that hamper goal achievement and to plan strategies of dealing with these barriers. Furthermore, the facilitators that could be used to help achieve the goal should be considered.
To achieve the aims described above each participant will analyse the goals established last week, as well as their behaviour self-monitoring pages (for both eating and PA in turn). After a

first analysis she/he will share this with another participant sitting next to her/him, describing what happened during the week, commenting on the SMARTness of the goal. The other person will reinforce any success (using reinforcers such as: "well done"; "you have made it"). After this participants will, in pairs, go through the same list of questions used during last session and reflect upon:

See ppt. slide 3

How to perform Activity 1: Reviewing eating and PA goals

"On your handout are some questions that you might want to consider when reviewing you achievements from last week. Team-up with your neighbour and review all your accomplishments from last week. Focus on eating first, but also make sure that you answer the same questions about PA. When you are reviewing the last week don't forget to chart the healthy eating and the steps. After that we will discuss in the group what were the barriers, facilitators and successes, and what new goals people would like to formulate."

See ppt. slide 4

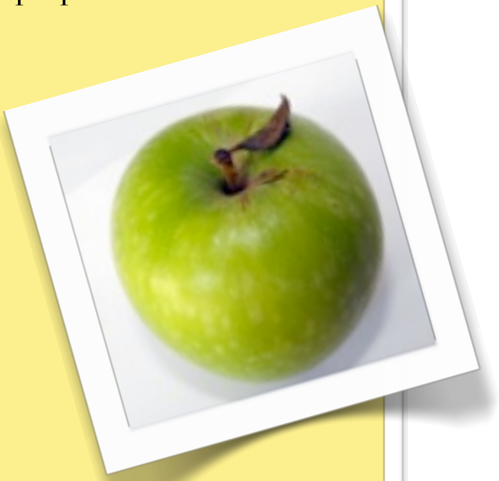

Questions that participants should consider are:

- What did I try to do (what was my goal)?
- How much effort did I invest to achieve my goal?
- What impact/success did I have?
- What benefits have I experienced?
- What difficulties have I encountered?
- How did I manage these difficulties?
- What made it easier?
- Am I satisfied with what I did during last week?

See ppt. slide 5

Pairs will then report back to the group focusing on: barriers; facilitators, success (e.g. showing their behaviour change chart) and the new goal. The facilitator will write on the flip chart the newly identified barriers (and facilitate discussion on possible answers).

See ppt. slide 6&7

After this exercise each participant will be instructed to write down a new SMART goal for both eating and PA, following the same protocol as in session 2 and 3. The facilitator will go around the room personally looking at each one of the distinct participants goals and check if they are establishing SMART goals and planning for good coping strategies to deal with barriers.

Session 4

Activity 2: Plan contingent rewards (40 minutes)

Technique descriptors: Contingent Rewards

Acknowledging success is not only important for the participant's progress towards his or her goals, it also boosts confidence. Often we don't acknowledge the things we have done well and tend to focus on the things we fail to do. By planning for the things we want to achieve and managing to do these we can keep ourselves motivated, and also feel good for the accomplishments.

An effective way of changing behaviour is to reward ourselves. Here the group members will be encouraged to add a system of rewards into their goals. This can have a positive impact on the participants, and increase the likelihood that the behaviour will occur. Rewards (or positive reinforcement) increase the chances that behaviour is performed. Participants should be encouraged/ instructed to reward themselves every time they reach their goal, or even a mini goal if their overall goal is a long-term goal.

A reward can be anything that the participant values having or doing, e.g. praise or treats.

The facilitator will emphasise that rewards don't have to cost money. If the participants can't think of any rewards, some examples will be provided. The facilitator will pass by the participants a box with different reinforcements written down (laminated examples). Each participant will remove 3 examples from the box. After this they will read them out loud for the whole group and discuss if the rewards would apply to them (the participants will also receive the full list in a handout format). Participants will also be told that rewards could be 'saved up'. For example, the money that they would have been spent on sweeties/crisps could be saved, or everyday that one has been physically active a certain amount of money could be saved (e.g. £1). The accumulated money could be spent on a reward at the end of the week/month.

The facilitator should alert the group members that some types of rewards are unhealthy and probably not in line with the overall goal that participants set for themselves. For instance if a participant is trying to increase the consumption of fruits and vegetables, a reward of a chocolate bar every time they eat 3 or more portions of fruit and vegetables in a day would not be a good idea or be congruent with their overall goal. The facilitator should encourage the members to choose rewards which would not affect their progress towards their global goal.

See
ppt. slide 8

How to perform Activity 2: Planning contingent rewards

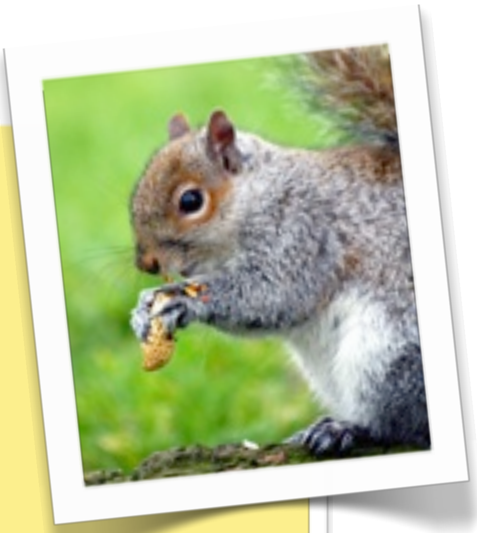

"Giving yourself little treats to reward yourself when you've made progress towards your goal can encourage you to keep going and make more progress. You don't have to wait until you've achieved your overall goal to reward yourself, remember that a big goal can be broken down into smaller mini-goals. Reward yourself for any mini-goals you reach, and any other small successes that you have along the way. You might want to think about smaller rewards for mini-goals, and bigger rewards for achieving longer-term goals. Rewards don't have to cost money, but you can also 'save up' for rewards. For example, save £1 every time you do some physical activity, then at the end of the week or the month spend the money on a reward for yourself. If you can't think of many rewards, here are some examples (verbally exchanged with the participants/ handout/flip chart) of pleasurable things you can do:

Some things I can reward myself with ...

Rewards that don't cost money	Rewards that cost money
Having a nice relaxing bath	Buying yourself a cd/magazine
Borrowing a book or magazine	Buying yourself new clothes
Inviting friends round	Going to the cinema
Having some 'me' time when you can do whatever you want to	Buying yourself flowers
Listening to music	Buying yourself sports equipment
Going for a walk in the garden	Going to a football match
Watching your favourite TV programme	Going out for a meal
Doing some gardening	Renting a DVD
Ask friends or family to look after your children so you can have some time for yourself	Book a holiday or weekend break
Ask friends or family to notice and praise you when you have achieved something.	Buying yourself some perfume/aftershave

See
ppt. slide 9

Warning!

"Try not to choose rewards that are unhealthy. For example, if you are trying to eat more healthily, rewarding yourself with a bar of chocolate every time you eat 5 portions of fruit and vegetables is not a good idea. Use the self-rewards exercise sheet to make a list of things you could reward yourself for, and what those rewards could be" (verbally exchanged with the participants/ handout).

After this an in a pair each participant will be asked by the other "what kinds of things would you like as a reward for achieving your goals and other little successes along the way?" Each participant will think about the rewards they would prefer and write these down on the reward sheet for future reference. If there is time participants will report back to the whole group the rewards they selected and the facilitator will register the favourite rewards on the flip-chart.

See ppt. slide 10

Activity 3: Weekly challenges (10 minutes)

- Self-monitoring of behaviour change: each participant will be recording each day what they have been eating and drinking as well as their levels of PA. In this record they will also register if they have or not achieved their "Eating goal" and "Activity goal" and if not, the reason why. They will continue using the pedometer.
- Each time the participant reaches his/her goals they should not forget to reinforce/reward themselves.
- Bring in a healthy snack for the next session.

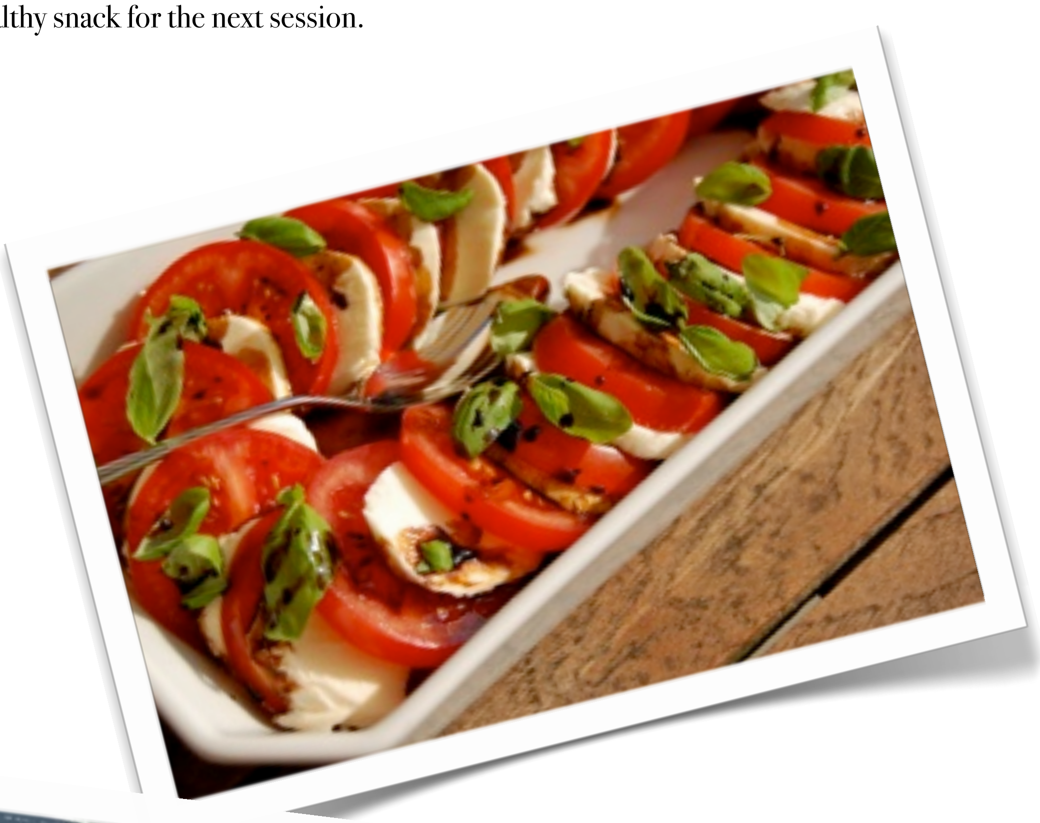

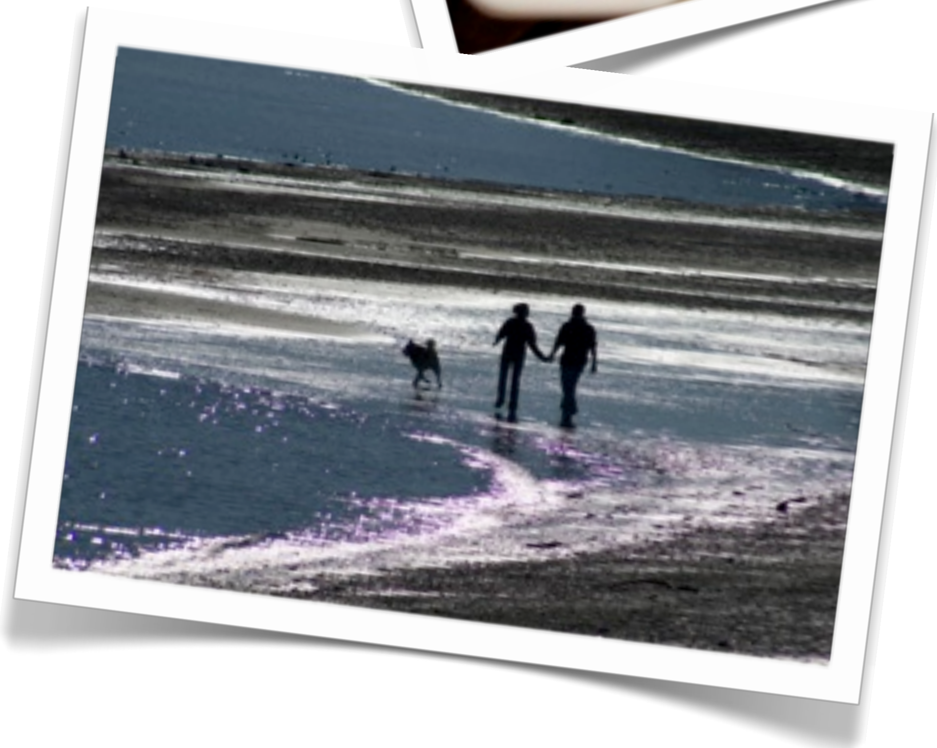

APPENDIX: SLIDES

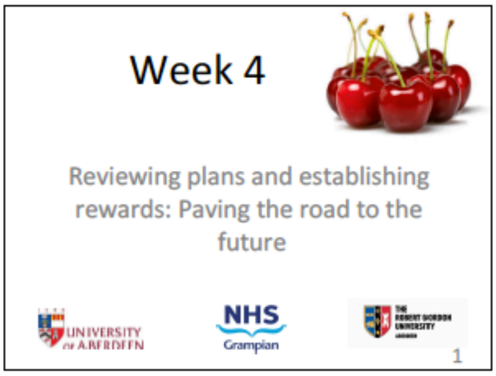

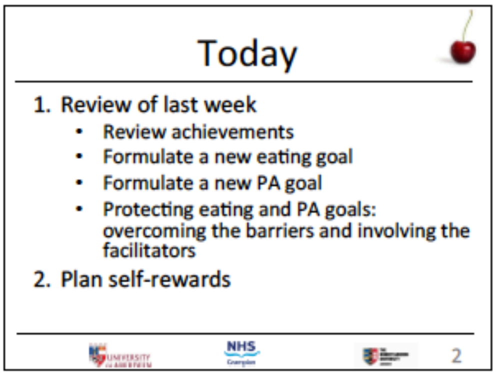

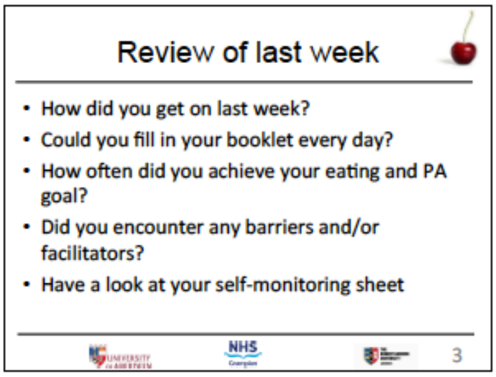

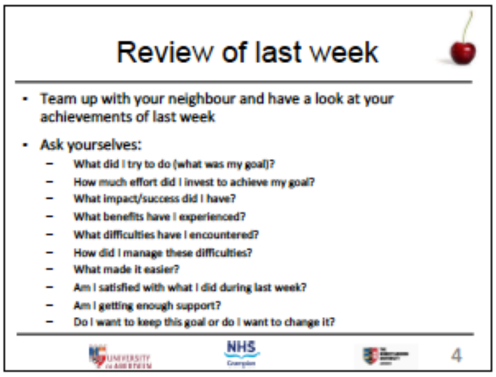

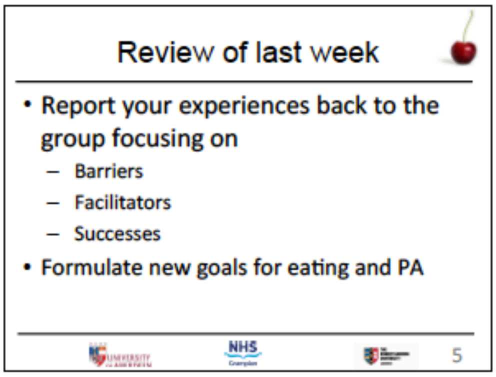

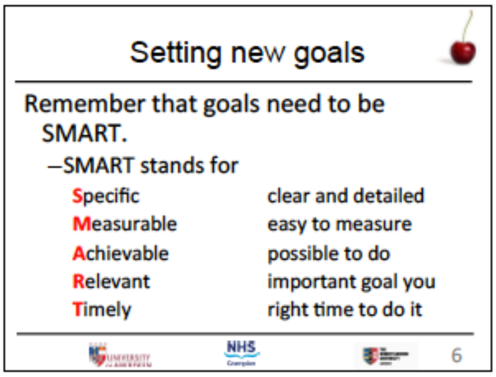

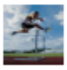

Barriers & Facilitators

Make sure you achieve your goal by planning for barriers...

- Unsupportive **friends/relatives**;
- A picky **family** that does not want to embrace your life change;
- Unsupportive **situations** e.g. a party;
- **Feeling** sad and depressed;
- Not having **time**.

... and involve facilitators

- **People** that encourage and support you;
- The **community**;
- Local **facilities**;
- **Reminders** or situations

7

Planning self-rewards

- It is a good idea to reward yourself when you have done well!
- There are plenty of things that can be treats for yourself when you achieved something.
- Plan in advance how to reward yourself for doing what you want to do!
- Have a look at the example cards for self-rewards.

8

Planning self-rewards

- Warning: don't choose unhealthy rewards.
- Rewarding yourself with a bar of chocolate every time you eat 5 portions of fruit and vegetables is not a good idea.
- Use the sheets in your weekly booklet to make a list of the three things you could reward yourself for, and what those rewards could be.

9

For next week

- Keep on monitoring your eating and PA.
- Use your weekly booklet and bring it with you to the next session.
- Have fun and see you next week for the final session.
- Remember to claim your travel expenses at the end of the last session.

10

APPENDIX: SELF-REWARDS SHEET

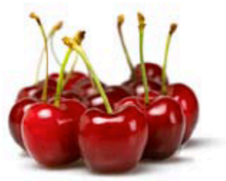

Self treats

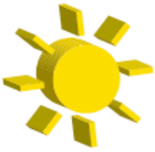

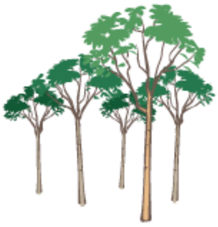

1. Soak in the bathtub.
2. Plan my career.
3. Collect shells.
4. Recycle old items.
5. Go on a date.
6. Buy flowers.
7. Go to a movie in the middle of the week.
8. Walk or jog.
9. Listen to music.
10. Recall past parties.
11. Buy household gadgets.
12. Read a humour book.
13. Think about my past trips.
14. Listen to others.
15. Read magazines or newspapers.
16. Do woodworking.
17. Build a model.
18. Spend an evening with good friends.
19. Plan a day's activities.
20. Meet new people.
21. Remember beautiful scenery.
22. Save money.
23. Go home from work.
24. Practice karate, judo, yoga.
25. Think about retirement.
26. Repair things.
27. Work on my car or bicycle.
28. Remember the words and deeds of loving people.
29. Wear sexy clothes.
30. Have a quiet evening.
31. Collect coins.
32. Take care of my plants.
33. Buy or sell stock.
34. Go swimming.
35. Doodle.
36. Collect old things.
37. Go to a party.
38. Think about buying things.

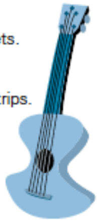

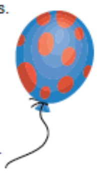

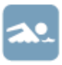

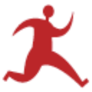

39. Play golf.
40. Play soccer.
41. Fly a kite.
42. Have a discussion with friends.
43. Have a family get-together.
44. Take a day off with nothing to do.
45. Arrange flowers.
46. Have sex.
47. Ride a motorcycle.
48. Practice religion (go to church, pray).
49. Go to the beach.
50. Sing around the house.
51. Go skating.
52. Paint.
53. Do needlepoint, crewel, knitting, sewing, etc.
54. Take a nap.
55. Entertain.
56. Go to a club meeting.
57. Go hunting.
58. Sing with groups.
59. Flirt.
60. Play a musical instrument.
61. Make a gift for someone.
62. Collect postcards.
63. Buy a record, tape, or CD.
64. Plan a party.
65. Go hiking.
66. Write a love poem.
67. Buy clothes.
68. Go sightseeing.
69. Garden.
70. Go to the beauty parlour.
71. Play cards, chess, etc.
72. Buy a book.
73. Watch children play.
74. Write a letter.

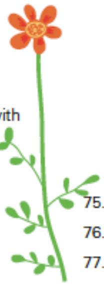

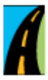

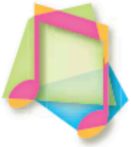

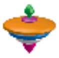

75. Write in a diary.
76. Go to a play or concert.
77. Daydream.
78. Plan to go to school.
79. Go for a drive.
80. Listen to music.
81. Refinish furniture.
82. Make lists of things to do.
83. Go bike riding.
84. Take a walk in the woods.
85. Buy a gift for someone.
86. Visit a national park.
87. Take photographs.
88. Go fishing.
89. Play with animals.
90. Read fiction.
91. Watch an old movie.
92. Go dancing.
93. Meditate.
94. Play volleyball.
95. Read nonfiction.
96. Go bowling.
97. Go to the mountains.
98. Think about happy moments in my childhood.
99. See or show photographs or slides.
100. Play cards, chess, checkers, etc.

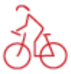

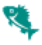

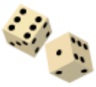

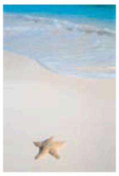

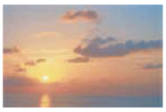

101. Solve riddles.
102. Have a political discussion.
103. Play softball.
104. Do crossword puzzles.
105. Shoot pool.
106. Dress up and look nice.
107. Think about how I've improved.
108. Buy something for myself (perfume, golf balls, etc.)
109. Talk on the phone.
110. Kiss.
111. Go to a museum.
112. Light candles.
113. Get a massage.
114. Say "I love you."
115. Take a sauna or steam bath.
116. Go skiing.
117. Have an aquarium.
118. Go horseback riding.
119. Do a jigsaw puzzle.
120. Go window shopping.
121. Send a greeting card to someone you care about.

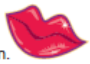

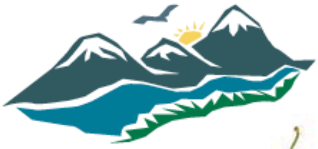

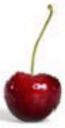

adapted from Diabetes Prevention Program, 2002

http://diabetes.niddk.nih.gov/dm/pubs/preventionprogram
