# Supplementary material for: Randomised Controlled Feasibility Trial of an Evidence-Informed Behavioural Intervention for Obese Adults with Additional Risk Factors
Source: PLoS One. 2011 Aug 29;6(8):e23040. doi: 10.1371/journal.pone.0023040 (PMC3163575; doi:10.1371/journal.pone.0023040)
Supplement: Intervention Manual S5 — Intervention Manual Session 5. (PDF) [file pone.0023040.s015.pdf]

The ABC Weight Loss Study

The Aberdeen Behaviour Change Study

Health Psychology Group
University of Aberdeen - August 2009

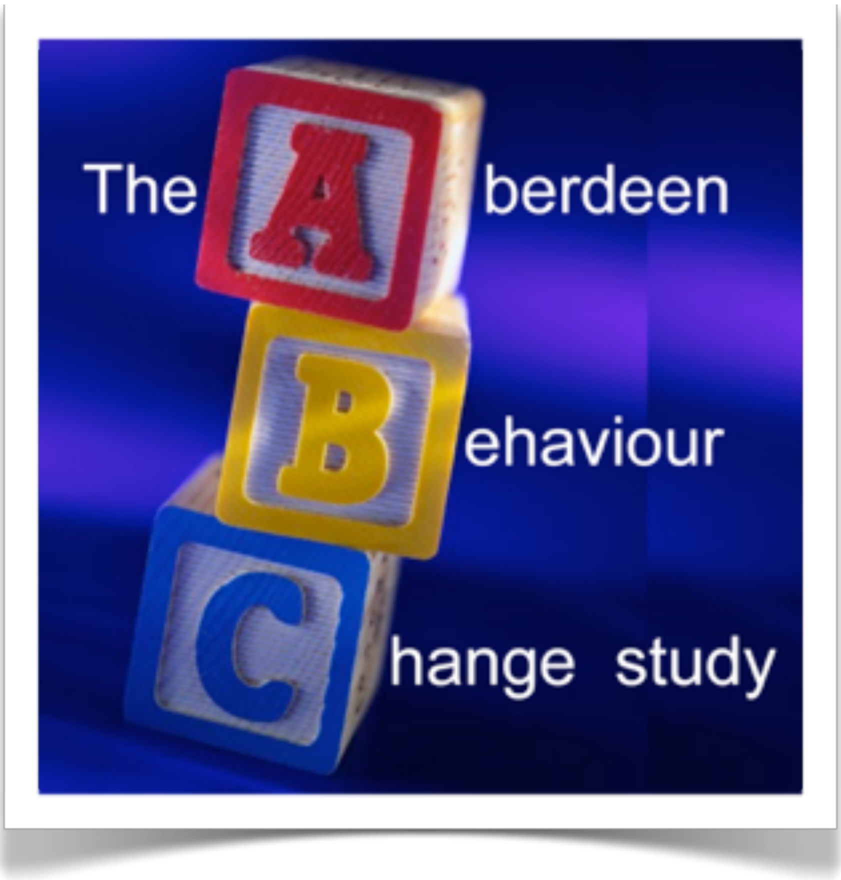

Authored by

Vera Araújo-Soares, Stephan Dombrowski & Falko Sniehotta

(in alphabetical order)

© University of Aberdeen

Session 5

Table of Contents

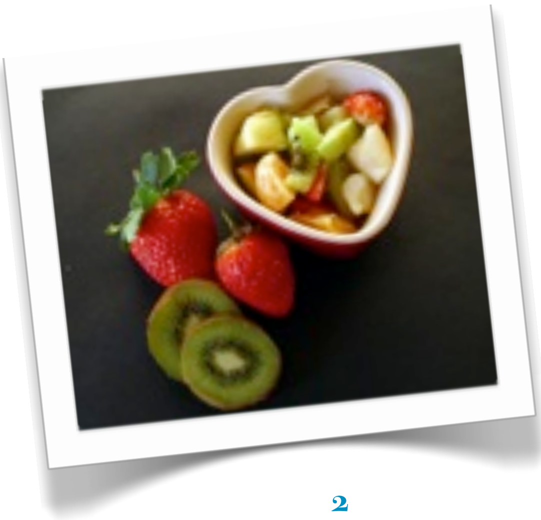

Overview 2

The Session Basics 2

Summary 2

Goals 2

Techniques 2

Materials 2

Recommended time 3

Before the fifth meeting 3

Activities and procedures 3

Content 4

Reviewing Achievements, Generalising Activities, promoting Maintenance 4

Activity 1: Review of last week (30 minutes) 4

Activity 2: Review contingent rewards (10 minutes) 6

Activity 3: Become aware of lapse and relapse issues (30 minutes) 7

Activity 4: Continue behaviour change and see you in 3 weeks (10 minutes) 10

Activity 5: Weekly Challenges (10 minutes) 11

Appendix: Slides 12

Overview

Detailed overview of session 5 content

THE SESSION BASICS

Summary

The purpose of the fifth session is to review achievements of previous weeks and to highlight all positive changes that have taken place. It will be emphasized that the achieved behaviour changes can be generalised to other behaviours. Moreover, relapse issues as part of an ongoing attempt for permanent lifestyle change will be discussed. After this session, the lifestyle changes continue, but participants will take charge, independently using their newly acquired skills. In three weeks they will come together again to assess how this new phase of their life is taking off (follow-up session).

Goals

1. Review whether established eating and PA goals have been attained during the week and formulate goals and coping plans based on the achievements, barriers and facilitators of the previous week.
2. Become aware of relapse prevention issues.
3. Continue being the manager of your behaviour in the future and 'see you later'.

Techniques

- Review of behavioural goals
- Action planning
- Coping planning
- Self-monitoring
- Relapse prevention *New*

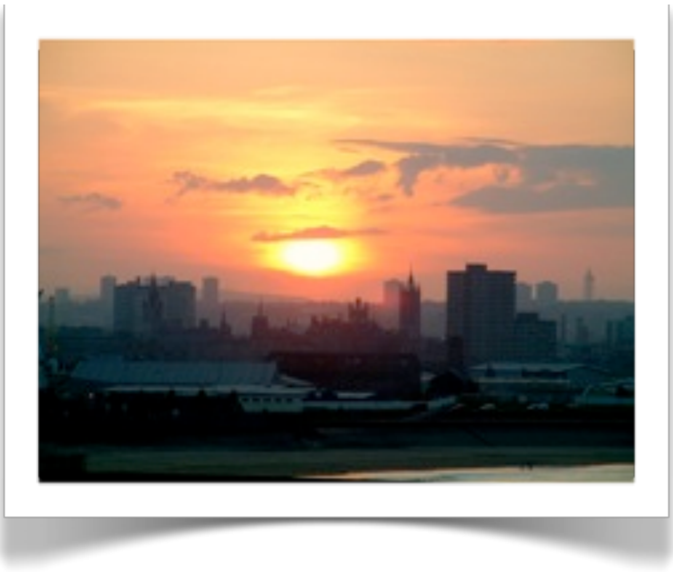

Materials

- Weekly booklet

Session 5

- Flipchart
- Healthy snack

Recommended time

- 90 minutes

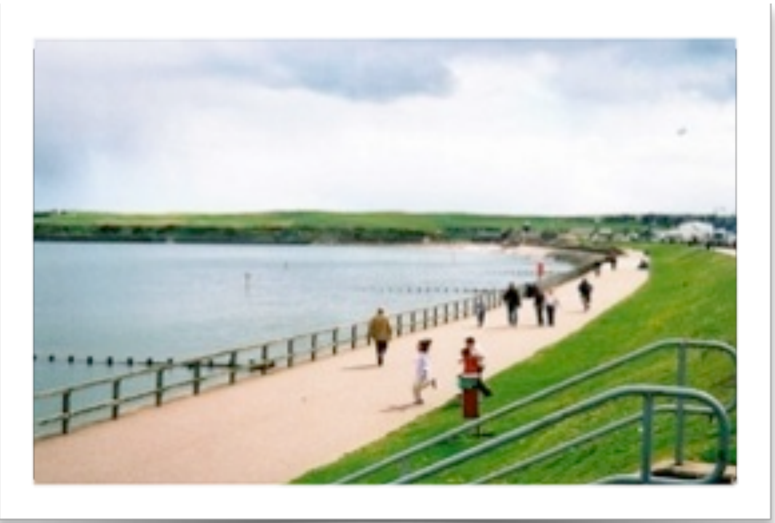

Before the fifth meeting

- Know how to introduce the fifth session;
- Have a clear idea about the structure of the fifth session;
- Have a clear understanding of the behaviour change techniques;
- Get materials ready (see materials section above).

Activities and procedures

In the remainder of this session description you will find a detailed guide to the activities you should facilitate in Session 5.

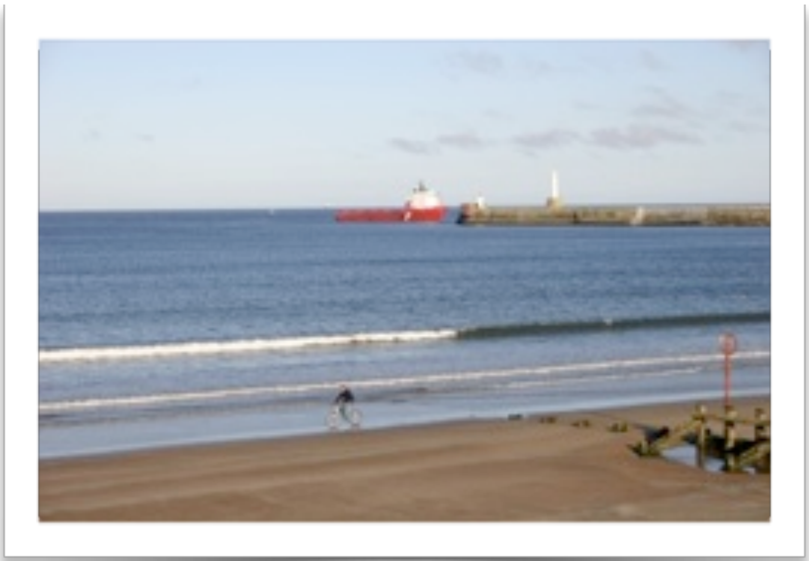

Content

REVIEWING ACHIEVEMENTS, GENERALISING ACTIVITIES, PROMOTING MAINTENANCE

See ppt. slide 2

Activity 1: Review of last week (30 minutes)

Following the outline of the session in the first part of the session participants will reconsider the goals set for eating behaviours and PA during the last session. Participants will analyse how they managed to act on those goals by reviewing the self-monitoring pages. The review will start with last week's eating goals and be followed by the PA/walking goals. The procedures will be similar to those used during session 3.

Participants will review if their eating and PA goal from the previous week were achieved and on many occasions. Barriers and facilitators encountered during the week will be discussed and analysed. If needed, new ways of overcoming barriers and involving facilitators will be generated. Participants will assess whether last week's goals was a SMART goal, and if there is any need to redefine this goal or to break it down into smaller goals. If the person has been successful in enacting the goal she/he should establish a new goal using the same steps as in the previous session, finding new "opportunities for change".

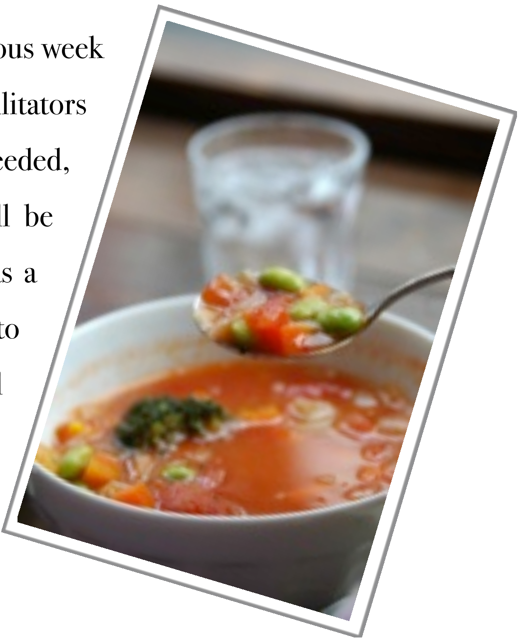

The facilitator should reinforce any efforts made towards attaining the goal and prompt the group to focus back on how it felt when the goal was attained (using questions such as "When you managed to achieve your goal, how did you feel?"). Even if participants only managed to attain the goal some of the time it should still be stressed that they are capable of achieving their goals. Participants should be encouraged to be more attentive to the barriers that hamper goal achievement and to plan strategies of dealing with these barriers. Furthermore, the facilitators that could be used to help achieve the goal should be considered.

Session 5

To achieve the aims described above, each participant will analyse the goals established last week as well as their behaviour self-monitoring pages (for both eating and PA in turn). After a first analysis she/he will share this with another participant sitting next to her/him, describing what happened during the week, commenting on the SMARTness of the goal. The other person will reinforce any success (using reinforcers such as: “well done”; “you have made it”). After this participant will, in pairs, go through the same list of questions used during last session and reflect upon:

See ppt. slide 3

How to perform Activity 1: Reviewing eating and PA goals

See ppt. slide 4

“On your handout are some questions that you might want to consider when reviewing you achievements from last week. Team-up with your neighbour and review all your accomplishments from last week. Focus on eating first, but also make sure that you answer the same questions about PA. When you are reviewing the last week don’t forget to chart the healthy eating and the steps. After that we will discuss in the group what were the barriers, facilitators, successes and what new goals people would like to formulate.”

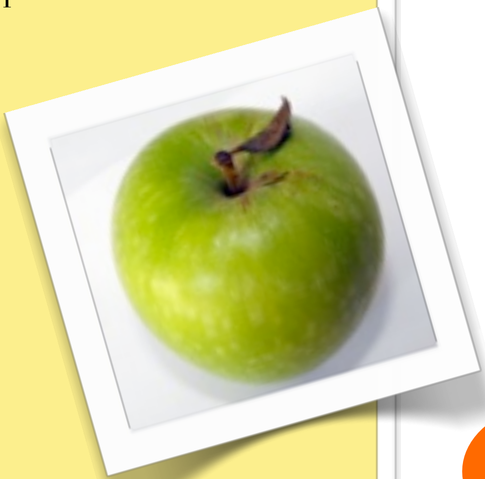

Questions that participants should consider are:

- What did I try to do (what was my goal)?
- How much effort did I invest to achieve my goal?
- What impact/success did I have?
- What benefits have I experienced?
- What difficulties have I encountered?
- How did I manage these difficulties?
- What made it easier?
- Am I satisfied with what I did during last week?

See ppt. slide 5

Pairs will then report back to the group, focusing on: barriers; facilitators, success (e.g. by showing the chart) and new goal. The facilitator will write on the flip chart the newly identified barriers (and facilitate discussion on possible answers).

See ppt. slide 6

After this exercise each participant will be instructed to write down a new SMART goal for both eating and PA, following the same protocol as in session 2 and 3. The facilitator will go around the room personally looking at each one of the individual participants goals and check if they are establishing SMART goals and planning for good coping strategies to deal with barriers.

See ppt. slide 7

Activity 2: Review contingent rewards (10 minutes)

The facilitator will prompt participants to review whether or not they successfully rewarded themselves for doing well. This should be addressed in a group discussion with every participant sharing his/her experience in turn. The facilitator will ask participants how it felt not only to achieve the goal, but also to receive a reward at the end of it. The importance of using rewards for all kind of behavioural achievement will be underlined.

See ppt. slide 8

How to Perform Activity 2: Reviewing self-rewards

"Remember that last week we talked about rewarding yourself when you have achieved a goal or mini goal.

Did you reward yourself last week?
Did you reward yourself after achieving something?
How did it work for you?
Has this helped you achieve your goal?
Have you planned your rewards for next week?"

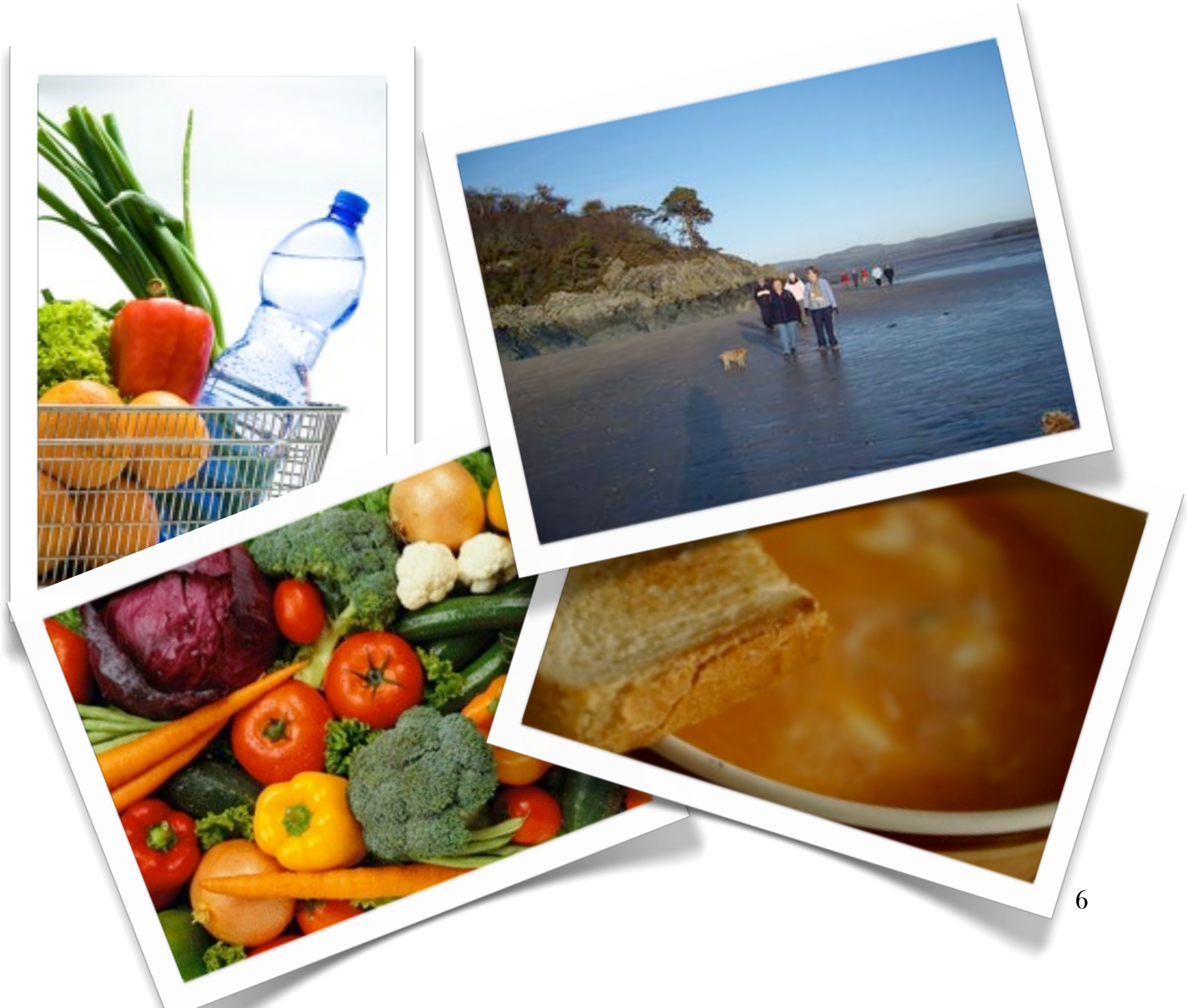

Activity 3: Become aware of lapse and relapse issues (30 minutes)

Technique description: Relapse prevention

Behaviour change is typically not a straightforward process. After changing behaviour for a while, it often happens that old habits catch us off guard. In these cases we need to be aware of when these lapses happen, and how we can respond to avoid complete relapse. Relapse prevention normalises lapses, and helps clients get back on track once a lapse has occurred.

It is vital to explore the possibility of risk situations triggering a lapse, and, if not addressed, a full relapse. In the case of eating and PA goals a lapse can be defined as not following one's goal once during one week (e.g. on one day when the behaviour was planned to be executed), and a full relapse can be defined as not following the plan more than once during one week. A lapse can be seen as something that occurs once when the old habits overwhelm the level of commitment to a new goal, but the goal itself is still present with the person still wanting to put it into practice. A relapse occurs once the person stops pursuing the established goal altogether, and abandons it (West, 2006).

It is important that the participant understands that a relapse is a process that begins well in advance of the actual event. People who relapse can usually point back to certain things that they thought and did long before they actually fell back into old habits. Hence the participant should be attentive to *warning signs* (the first signs of lapse) that may lead to a lapse and take positive steps to stay on track and keep to their goals, acting immediately to counteract the possibility of a lapse.

The facilitator should encourage participants to identify things that could put them at risk for a lapse and to discuss various strategies (behaviour change techniques) learned during past sessions which could be used to prevent a lapse before it occurs. As learned at the beginning of the programme, it is important to identify (as a detective) the risk situations that could lead to a lapse (or that have brought a lapse in the past) and then formulate protection plans. Having confidence in the strategies acquired during previous sessions will be crucial, as well as being able to replicate/generalise these. Participants should be encouraged to, from time to time, review their lapse and relapse prevention plans.

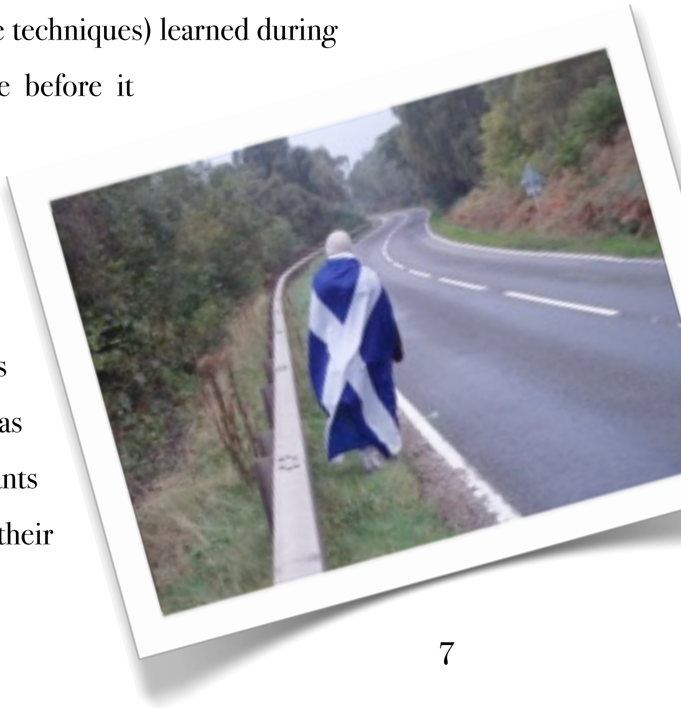

See ppt. slide 9

How to perform Activity 3: Relapse prevention

"Along the previous sessions and as detectives in your own backyard, you have become familiar with situations/thoughts/feelings/contexts and people that might restart you on old habits. Now, after having come to our 5 sessions, and after having learned about and used various strategies, the potential to lapse, and later relapse is minimised. However, this does not mean that it cannot occur."

"If and when lapses happen, you should not judge or blame yourself. You are not a failure or a bad person because of this. Do not feel that all is lost once a lapse occurs. You can always learn from a lapse and relapse, and prevent it from happening again in the future. So the best thing to do if you have a lapse is to get right back on track towards your goals as soon as possible. Rather than spending time and effort on being upset, direct your activities towards regaining control. "

See ppt. slide 10

"Also, if you are prepared for the possibility of a lapse you are more likely to keep it from becoming a full relapse. Remember that the process of change (that we have discussed in the first session) is filled with ups and downs. The downs are critical moments on which to focus back on success and regain control"

"Almost everyone who is in the process of maintaining changes has times when competing thoughts/cravings and old habits return (eating the same things, behaving in the same way). However, you should remind yourselves of the reasons why you wanted to change, as well as all the health and personal problems that your previous behaviours have caused you. Remember that no matter how hard it sometimes feels to stick to your own plans, the gain in the long run will far outweigh any short term pleasure that might be found in past habits. To establish new habits and stick to them takes time. Your cravings, and occasional temporary doubts in your own power (feeling of self-efficacy) of sticking to your initial behaviour change, will fade if you manage not to give in."

"You might find that the gains seem small in the beginning but they will accumulate bit by bit with time as a new sense of control evolves in your new way of living a healthier lifestyle. Also, when the commitment to change is firm, and what we stand to gain outweighs going back to the old behaviours enormously, chances are that we will stick to our new behaviours"

See ppt. slide 11

"In order to be able to recognise the risk situations for a potential lapse or relapse, it is necessary to pinpoint internal and external risk factors within each participant. These can include (Marlatt & Donovan, 2005):"

1. *Negative feelings* such as anger frustration, anxiety, depression, boredom). [Caused by intrapersonal perceptions of certain situations (e.g. feeling bored or lonely after coming home from work) or by reactions to environmental events (e.g. being made redundant)]
2. *Relationship difficulties* (e.g. situations involving conflict associated with any interpersonal relationship, in particular family or friends).
3. *Social pressure* (e.g. situations in which you respond to the influence of another person or group of people who exert pressure for you to engage in a non desired behaviour).
4. *Positive feelings* (e.g. celebrations), exposure to unhealthy eating -related stimuli or cues.

Marlatt GA, Donovan,DM (Eds) (2005): Relapse Prevention: Maintenance strategies in the treatment of addictive behaviors

See
ppt. slide 12

"Breaking these situations down in specific units, some possible factors and warning signs for Lapse/Relapse might be:

1. Hanging around with people that you know do not support your new behaviours
2. Isolating, or not seeking others for support (e.g. not using the telephone to seek support)
3. Keeping cookies, chocolates and other sweets around the house for any reason
4. Going shopping with a strong feeling of "starvation"
5. Relationship difficulties, ongoing serious conflicts (e.g. a spouse that boycotts your efforts for healthy eating)
6. Setting unrealistic goals (e.g. perfectionism, being too hard on yourself)
7. Changes in sleeping patterns, personal hygiene, or energy levels
8. Feeling overwhelmed, confused, useless, stressed
9. Constant boredom or irritability, or a lack of routine and structure in life
10. Major life changes like a loss or other painful emotions, or even winning the Euro Millions!
11. Ignoring lapse/relapse warning signs."

And ...Here are also some examples of situations that can restart unexpected cravings:
1. Something seen on TV or in a magazine
2. A smell
3. A sound that you might associate with your past habits
4. A particular feeling or mood

See
ppt. slide 13

"No matter how much you have invested on changing your behaviour during the past weeks, there's always the possibility of facing unexpected situations/emotions/thoughts/contexts/cravings that can bring you to lapse. Having a plan which defines how to deal with warning events/risk factor means that you can identify risk situations, which will help you to cope with these situations. Besides this, maintaining the behaviours you decided to change will allow you to manage obstacles/barriers to keeping on track with greater confidence."

After sharing this information the participant is encouraged to pick up their folder and to look into the sections on "protecting my goals". Here the participant will be able to list all the coping plans devised/developed across the previous sessions for managing different risks.

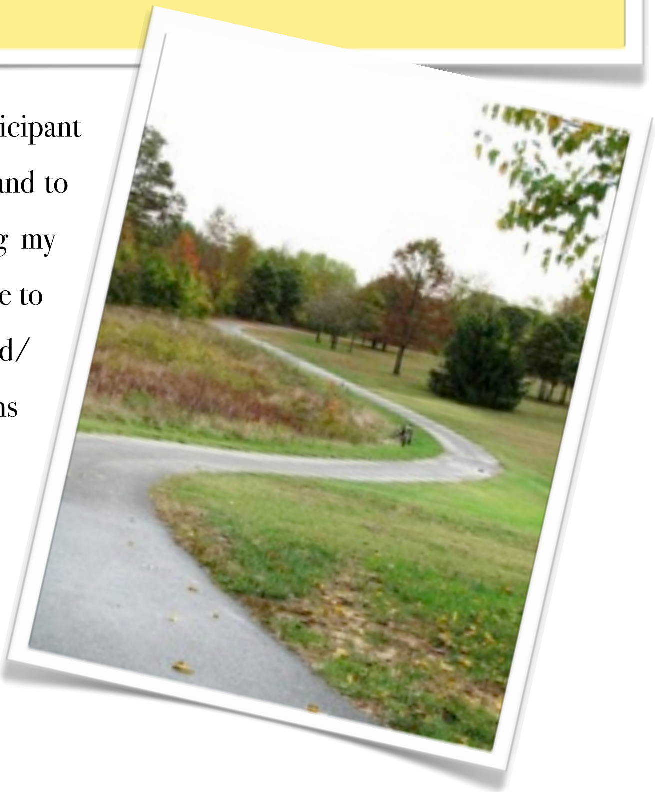

How to protect yourself from cravings that come when you are alone?

"You should be aware of the fact that some of the risk situations, such as cravings, can happen when you are alone. Here are different ways of dealing with these feelings:

1. Call someone that his in a similar situation than yours. It is specially important to have support from a significant other that will be easily available any time of day or evening and who is very accessible and meets your specific needs. Sometimes sharing your (re) lapse experience with someone else will allow you to have some time to think of what went wrong.
2. Arrange to go out with some friends.
3. Go for a walk and clear your head. At the same time you will be fulfilling your activity goal!
4. Engage in something else that gives you pleasure (think about the rewards you have defined for yourself last week)
5. Make sure you do not have "junk food" or food that you do not want to eat around the house.
6. Be sure to stick to your daily eating plan, if you are not hungry is easier to fight these cravings so do not skip any meals."

Idiosyncrasy is the key word, as we have noticed all across the intervention programme! Hence, each person lapse and relapse risk factors are unique to them, their lives, problems, goals and aims. This way, personal plans on how to deal with these, should be established for each participant. By the end of this activity each person should have coping strategies prepared that will allow them to come out of a lapse, and prevent a relapse if the strategies to avoid the risk factors for lapse do not work.

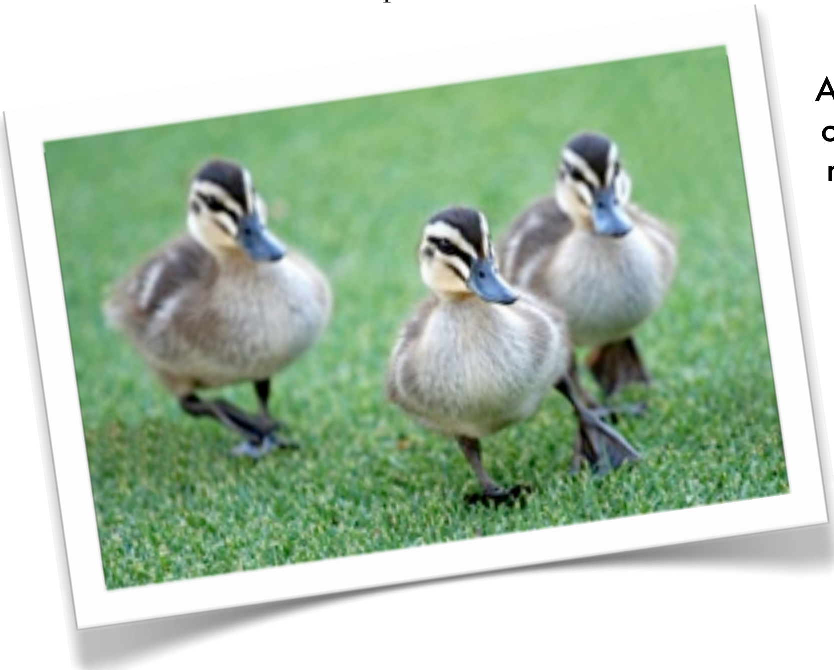

Activity 4: Continue behaviour change and see you in 3 weeks (10 minutes)

See ppt. slide 14

The facilitator will deliver certificates of attendance and competence on the strategies used across the sessions. These strategies should be mentioned by the names given during the programme. Each participant will be called by name and receive the certificate, as well as a pocket card (laminated card) with all the strategies named that she/he could carry in their pocket. The facilitator will

encourage the participants to use the strategies that have been learned across the sessions in their daily life, generalising them to different life situations.

After this a healthy snack should be provided (by the end of session 4 participants were asked to bring a healthy snack - the facilitator will have brought health snacks just in case no one volunteers to bring these) and the participants should be given the opportunity to socialise. This time should also be used to give general positive feedback and encouragement to the group members, stressing that to lose weight, and to maintain this loss, is part of a new lifestyle and a life task that, like life, is filled with little pleasures and sometimes little pains. “Persisting in our own goals is the trick and knowing precisely what you want!”.

To say good-bye allows the expression of feelings amongst the participants and the group facilitator. The facilitator should, again, refer to the need to continuously discover possible obstacles and problems arising.

Activity 5: Weekly Challenges (10 minutes)

- Repeat to yourself every morning what are your plans.
- Observe what happens around you, and the impact these things may have on your own behaviour. Be aware, keep on self-monitoring your behaviour. When needed establish new plans and new protecting plans.
- Expect a lapse, but know that you have precise plans on how to deal with it in order to prevent a relapse.
- Believe that you can do it.
- Reinforce/reward yourself.
- Use these strategies in other areas of life, you will see they help

APPENDIX: SLIDES

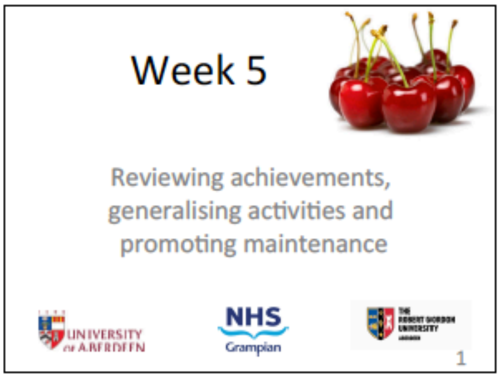

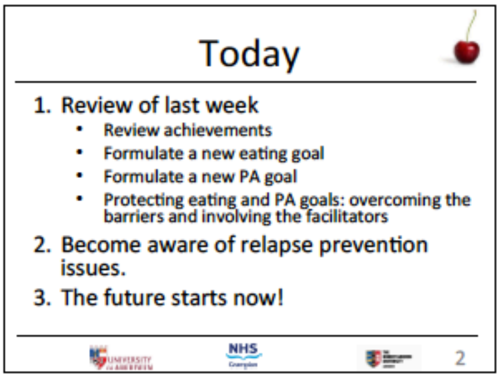

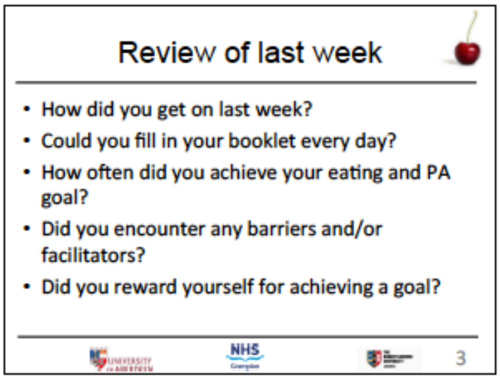

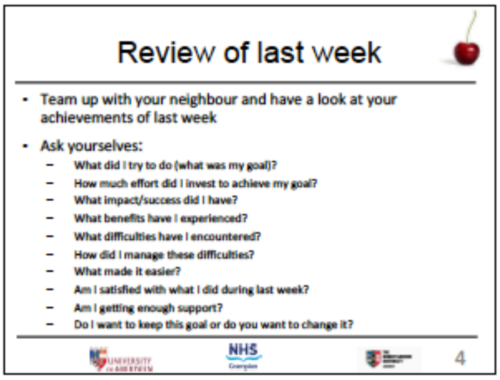

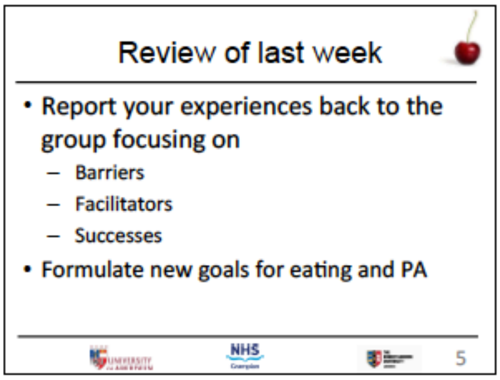

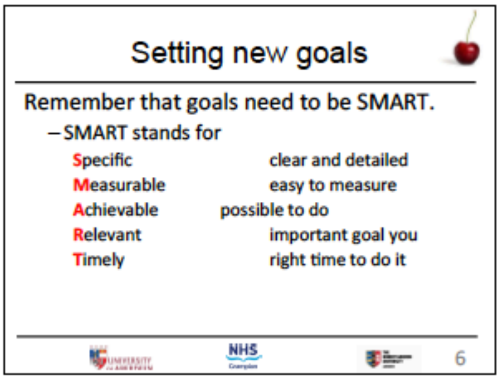

Barriers & Facilitators

Make sure you achieve your goal by planning for barriers…

- Unsupportive **friends/relatives**;
- A picky **family** that does not want to embrace your life change;
- Unsupportive **situations** e.g. a party;
- **Feeling** sad and depressed;
- Not having **time.**

… and involve facilitators

- **People** that encourage and support you;
- The **community**;
- Local **facilities**;
- **Reminders** or situations

7

Review self-rewards

- Have you rewarded yourself last week?
- Did you do it after achieving something?
- How did it work for you?
- Has this helped you achieve your goal?
- Have you planned your rewards for next week?

8

Relapse prevention

- After our 5 sessions you have learned and used many behavioural strategies:
  - Self-monitoring
  - Setting SMART goals
  - Achieving your goal (avoiding barriers and involving facilitators)
  - Reviewing achievements
  - Planning self-rewards
- The potential to fall into old habits is minimised.
- But, it doesn't mean that it will never happen.

9

Relapse prevention

- If and when lapses happens:
  - Do not judge or blame yourself (remember that the process of change is full of ups and downs).
  - Take it as an opportunity to learn and prevent it from happening again.
  - The best thing to do if you have a lapse is to get right back on track towards your goals.
  - Regain control!

10

Relapse prevention

- Lapse or relapse can be triggered by *internal* (e.g. feelings or thoughts) and *external* (e.g. environment). These include:
- **Negative feelings**, e.g. anger, frustration, anxiety, depression, boredom. This is often caused by perceptions of certain situations (e.g. feeling bored) or by reactions to environmental events (e.g. being made redundant)
- **Relationship difficulties**, e.g. situations involving conflict associated with any, in particular family or friends.
- **Social pressure**, e.g. situations in which you respond to the influence of another person or group of people who pressure you to engage in something not planned.
- **Positive feelings**, e.g. celebrations and parties, exposure to unhealthy eating - related stimuli or cues.

11

Relapse prevention

Here is a list of typical situations that lead to lapses and relapses:

- Hanging around people that don't support your new behaviours.
- Isolating, or not seeking others for support.
- Keeping cookies, chocolates and other sweets around the house.
- Going shopping with a strong feeling of "starvation" .
- Relationship difficulties, ongoing serious conflicts (e.g. a spouse that boycotts your efforts for healthy eating).
- Setting unrealistic goals (e.g. perfectionism, being too hard on yourself)
- Feeling overwhelmed, confused, stressed.
- Constant boredom or irritability, or a lack of routine and structure in life
- Major life changes like a loss, or even winning the lottery!
- Ignoring lapse/relapse warning signs.

12

Relapse prevention

- Having a **plan** on how to deal with warning events/risk factors that you can identify as risk situations will help you to cope with these.
- Besides this maintaining the behaviours you decided to change will allow you to increase your **confidence** and manage obstacles/ barriers for keeping on track.

13

For the next few weeks

- Keep up the good work!
- Continue to use the strategies that you learned in this programme.
- Write down the date of the next session on your weekly booklet

14
