# Supplementary material for: Randomised Controlled Feasibility Trial of an Evidence-Informed Behavioural Intervention for Obese Adults with Additional Risk Factors
Source: PLoS One. 2011 Aug 29;6(8):e23040. doi: 10.1371/journal.pone.0023040 (PMC3163575; doi:10.1371/journal.pone.0023040)
Supplement: Intervention Manual S6 — Intervention Manual Session 6. (PDF) [file pone.0023040.s016.pdf]

The ABC Weight Loss Study

The Aberdeen Behaviour Change Study

Health Psychology Group
University of Aberdeen - August 2009

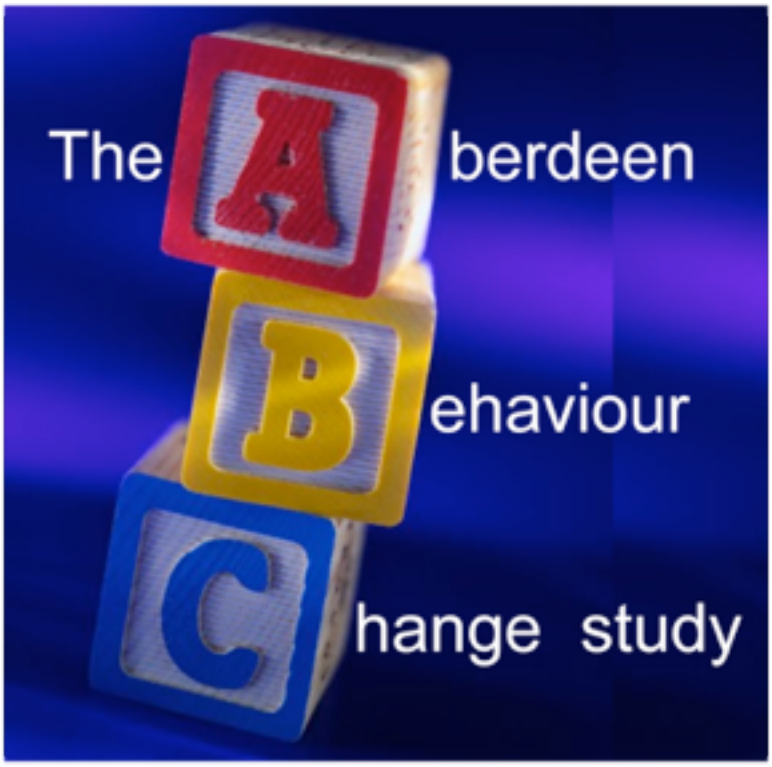

Authored by

Vera Araújo-Soares, Stephan Dombrowski & Falko Sniehotta

(in alphabetical order)

© University of Aberdeen

Table of Contents

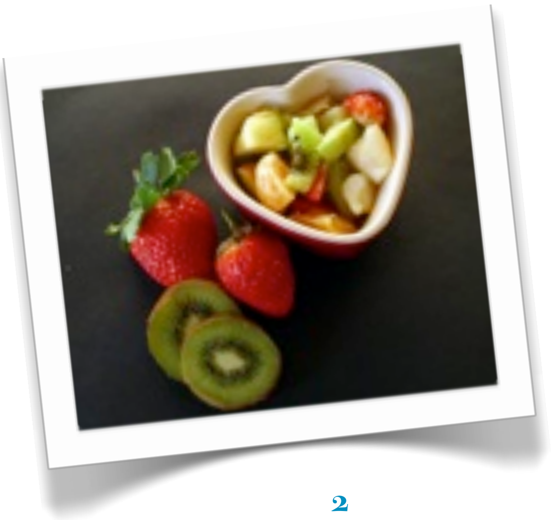

Overview 2

The Session Basics 2

Summary 2

Goals 2

Techniques 2

Materials 3

Recommended time 3

Before the sixth meeting 3

Activities and procedures 3

Content 4

Reviewing, Revinforcing and Consolidating Achievements 4

Activity 1: Review of last month (20 minutes) 4

Activity 2: Review contingent rewards (20 minutes) 7

Activity 3: Discuss lapses, relapses and ways of dealing with these (20 minutes) 7

Activity 4: Continue behaviour change and goodbye (20 minutes) 9

Activity 5: Life Challanges (10 minutes) 10

Appendix: Slides 11

Overview

Detailed overview of session 6 content

THE SESSION BASICS

Summary

The purpose of the sixth session 6 is to review, reinforce and consolidate achievements reached by participants since the end of the weekly intervention. The main goal is to highlight all positive changes that have taken place and provide reinforcement for these changes. It will be explored with the participants if, when and how generalisations of the behavioural change strategies have occurred, and what other goals/behaviours were selected as a target in order to facilitate weight loss. The facilitator will follow the assumption made at the last intervention session (session 5) that participants take charge of their behaviour change independently, using the acquired skills. The participants will be introduced to the concept of habit as a way of facilitating the implementation of the behavioural changes agreed upon. Also, relapse issues as part of an ongoing attempt for permanent lifestyle change will be discussed.

Goals

1. Review, reinforce and consolidate achievements
2. Explore relevant generalisations of the behavioural change skills for weight loss
3. Identify past relapse episodes and how the participants dealt with these
4. Continue being the manager of your behaviour in the future and good-bye.

Techniques

- Review of behavioural goals
- General encouragement
- Providing feedback on behaviour
- Self-monitoring
- Relapse prevention

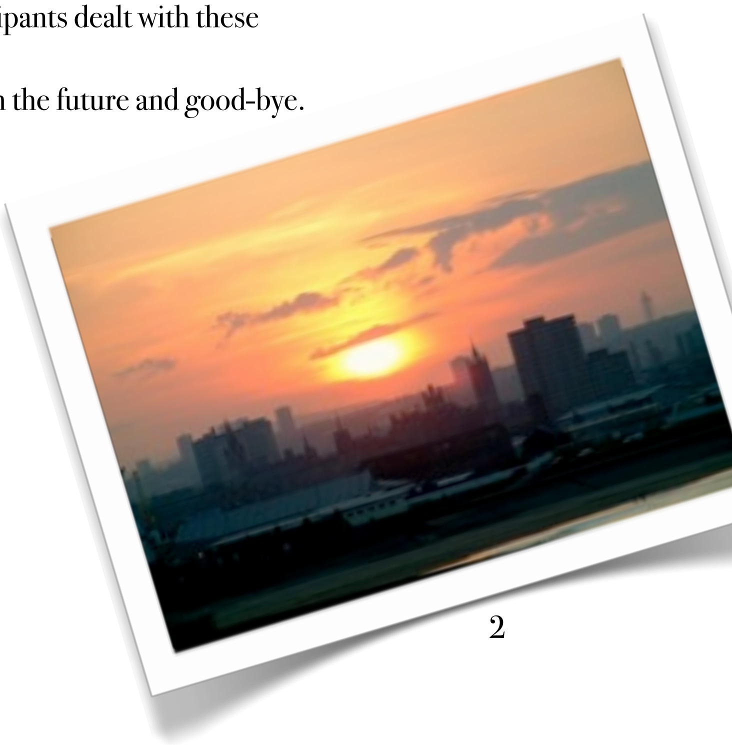

Materials

- Flipchart
- PowerPoint

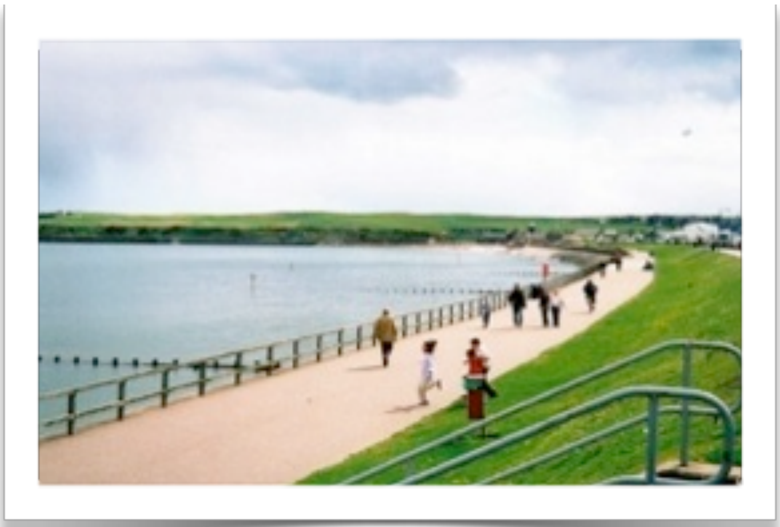

Recommended time

- 90 minutes

Before the sixth meeting

- Know how to introduce your role and the overall session structure;
- Have a clear idea about the structure of session 6;
- Have a clear understanding of the behaviour change techniques;
- Get materials ready (see materials section above).

Activities and procedures

In the remainder of this session six you will find a detailed guide to the activities you should facilitate in Session 6.

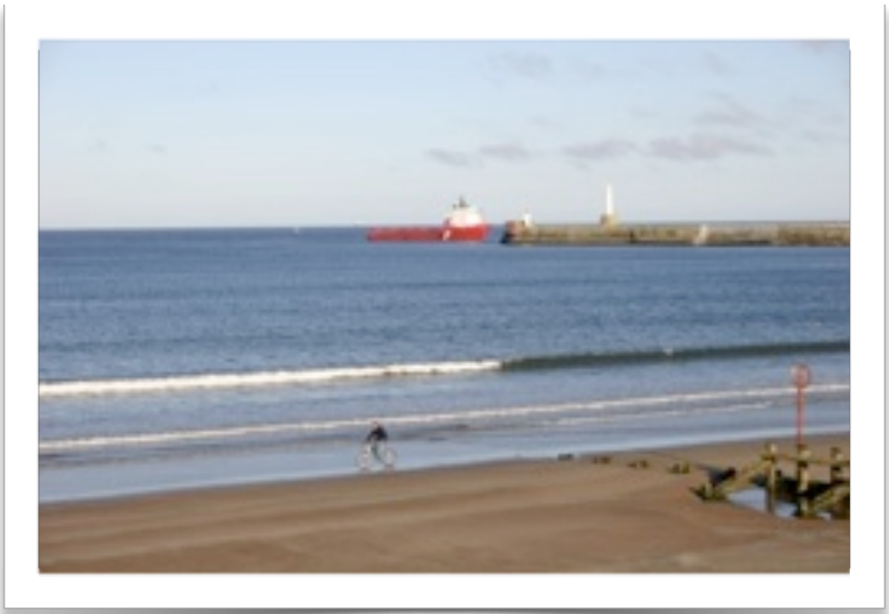

Content

Follow-up session

REVIEWING, REVINFORCING AND CONSOLIDATING ACHIEVEMENTS

See ppt. slide 2

Activity 1: Review of last month (20 minutes)

Following the introduction of the session structure the first part of the follow-up session participants will share the goals set for eating and PA behaviours established by them since the last session. Participants will analyse how they managed to act on those goals by reviewing self-monitoring sheets. The review will start with eating goals followed by PA/walking goals. The procedures will be similar to those used during session 3, 4 and 5.

See ppt. slide 3

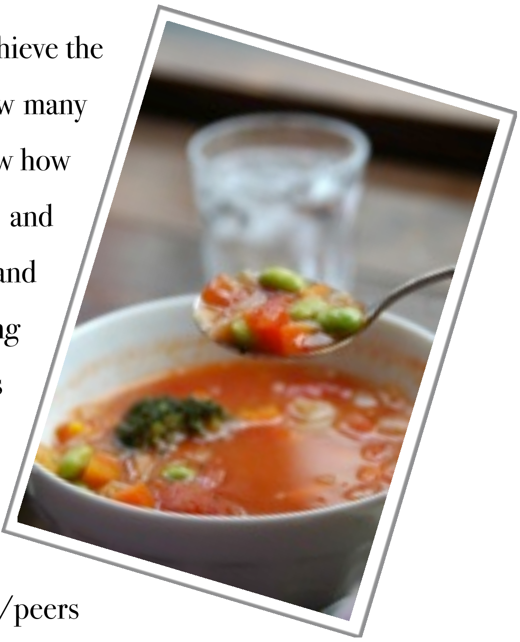

Participants should start by identifying if they have managed to achieve the established goals for eating/PA since the last session and on how many days they have managed to achieve it. Participants will also review how many new goals they have established and achieved. Barriers and facilitators encountered during the weeks will be discussed and analysed. If needed, new ways of overcoming barriers and involving facilitators will be generated. Participants will critically assess whether the goals established after the main part of the programme were SMART, and if there is any need to reformulate any of these goals, or to break them down into smaller goals. Each person should be reinforced by the facilitator/peers on the successful enactment of the goals she/he has achieved in previous sessions, and new "opportunities for change" should be sought and planned for.

The facilitator reinforces any efforts made towards attaining new goals, and prompts the group to focus back on how they felt when the goal was attained (using questions such as "When you managed to achieve your goal, how did you feel?"). The facilitator also reinforces the participant's capability to generalise the behavioural change skills when selecting new behaviours to change and/or initiate. Even if participants only managed to attain their goals some of the time, it should still be stressed that they are capable of achieving their goals. They

should be encouraged to be even more attentive to the barriers that hamper goal achievement and to plan strategies for dealing with these barriers. This task should now be easier to conduct given their experience with behaviour change and, importantly, self-monitoring. Furthermore, participants should activate the identified facilitators that can be used to help attain their goals.

To achieve the above aims each participant will analyse the goals established since the last session (Session 5), as well as their self-monitoring sheet (for eating and PA). After a first analysis he/she will share this with another participant sitting next to her/him, describing what happened ever since the main part of the programme finished, commenting on the SMARTness of the goals. The other person will reinforce any success (using reinforcers such as: "well done"; "you have made it"). After this the facilitator will distribute to each participant the same list of questions used during session 4&5 and prompt each participant to reflect upon:

See ppt. slide 4

How to perform Activity 1: Review of eating and PA goal

"On this slide are some questions that you might want to consider when reviewing your achievements up to now. Team-up with your neighbour and review all your accomplishments. Focus on eating first, but also make sure that you answer the same questions about PA. When you are reviewing the last weeks don't forget to chart the healthy eating and the steps. After that we will discuss in the group what were the barriers, facilitators, and successes of your new goals, as well as what new goals people would like to formulate, or if you will now focus on developing and consolidating new achievements, transforming those into habitual patterns of behaviour."

Questions that participants should consider are:

- What did I try to do (what were my goals)?
- How much effort did I invest to achieve my goals?
- What impact/success did I have?
- What benefits have I experienced?
- What difficulties have I encountered?
- How did I manage these difficulties?
- What made it easier?
- Am I satisfied with what I did during last week?
- Am I getting enough support?
- Do I want to keep this goal or do I want to change it?

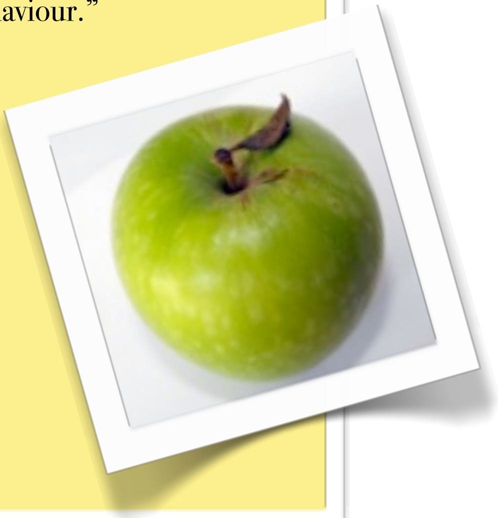

See ppt. slide 5

Session 6

Pairs will then report back to the group, focusing on: barriers; facilitators, success, new goal, and habits. The facilitator will write on the flip chart newly identified barriers (and facilitate discussion on possible answers). The facilitator will also write down the most relevant facilitators identified as well as the successes encountered. When participants refer to new goals, or conclude that the behaviour aimed for has become an habit, this will also be written down, then the positive characteristics of a habit and habit strength (automaticity, cue response) will be discussed.

During and/or after this exercise each participant will be reinforced by the other participants as well as by the facilitator. The facilitator will also provide individual feedback, going around the room personally. The facilitator will encourage each one of the participants to continue to use the behavioural change skills learned in order to establish and reach new behavioural goals, increasing their ability to establish SMART goals and to plan good coping strategies to deal with barriers.

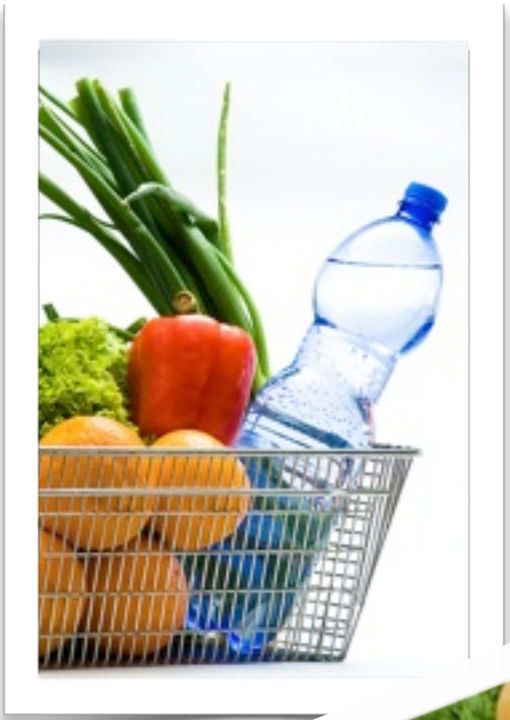

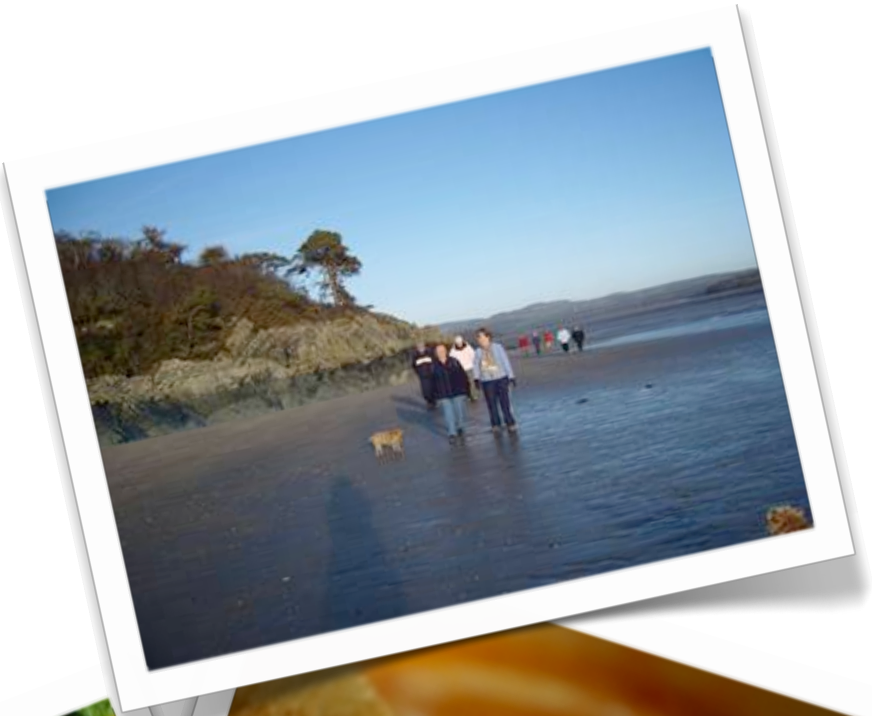

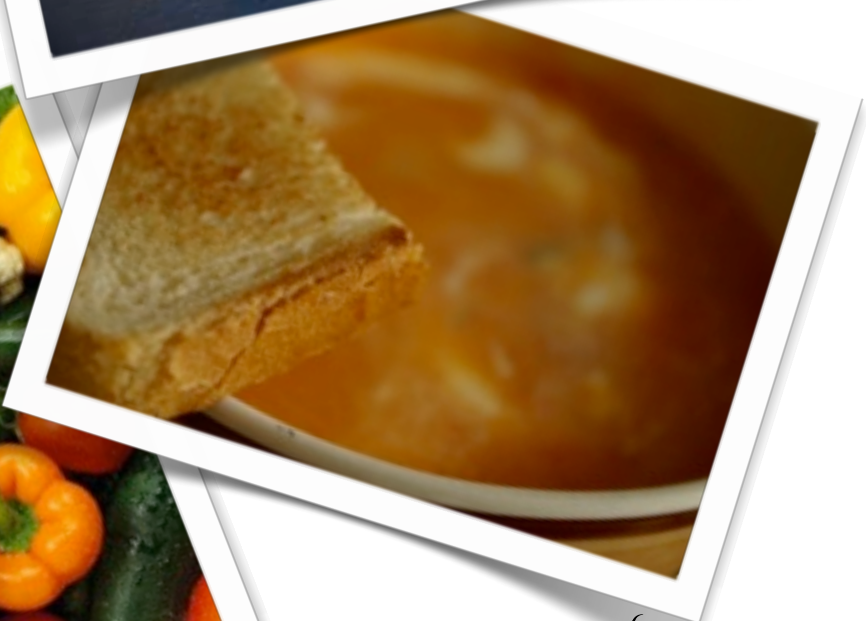

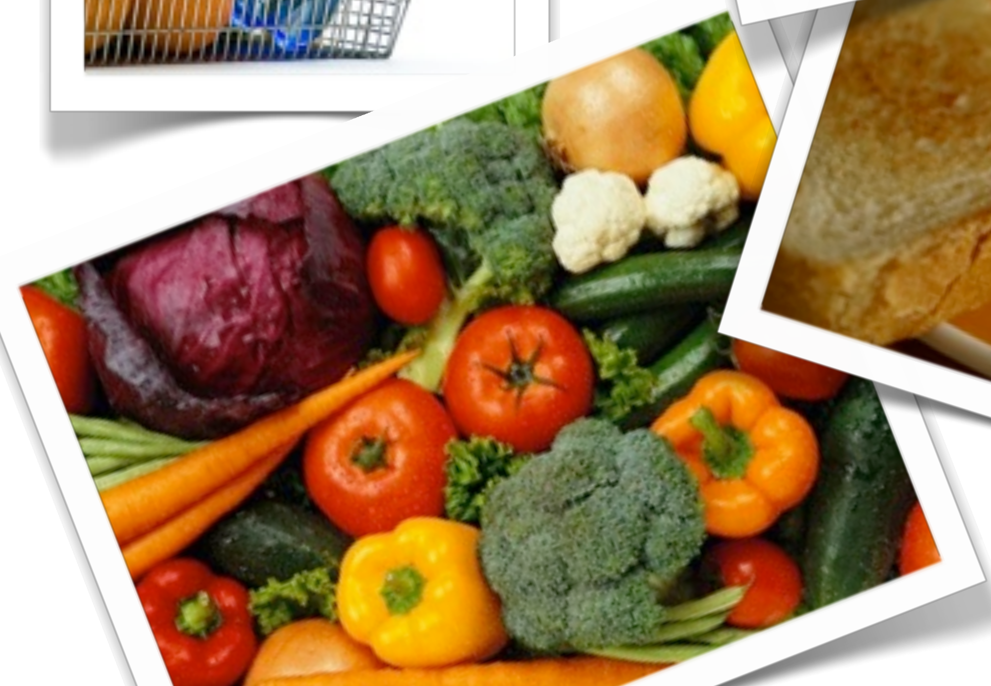

See ppt. slide 6

Activity 2: Review contingent rewards (20 minutes)

The facilitator will prompt participants to review whether or not they successfully rewarded themselves for doing well. This should be addressed in a group discussion with every participant sharing his/her experience in turn. The facilitator will ask participants how it felt not only to achieve the goal, but also to receive a reward at the end of it. The importance of using rewards for all kind of behavioural achievement will be underlined.

Activity 3: Discuss lapses, relapses and ways of dealing with these (20 minutes)

Even more important than reviewing if lapse or relapse situations have occurred, is for each participant to catalogue the most frequent risk situations that trigger these situations. The facilitator should encourage participants to review/identify the things/situations/people that have put them at risk for lapse and discuss the various strategies (behaviour change techniques) used since the last session to prevent new lapses. As learned at the beginning of the programme, it is important to identify/pinpoint (as a detective) the risk situations that brought on, and/or could lead to a lapse, and then formulate protection plans that have worked in the past, or create new ones based on the newly acquired experiences. Having confidence in the strategies acquired during the previous month and being able to replicate/generalise these will be crucial. Participants will, during this task, be encouraged to state and review their lapse and relapse prevention plans.

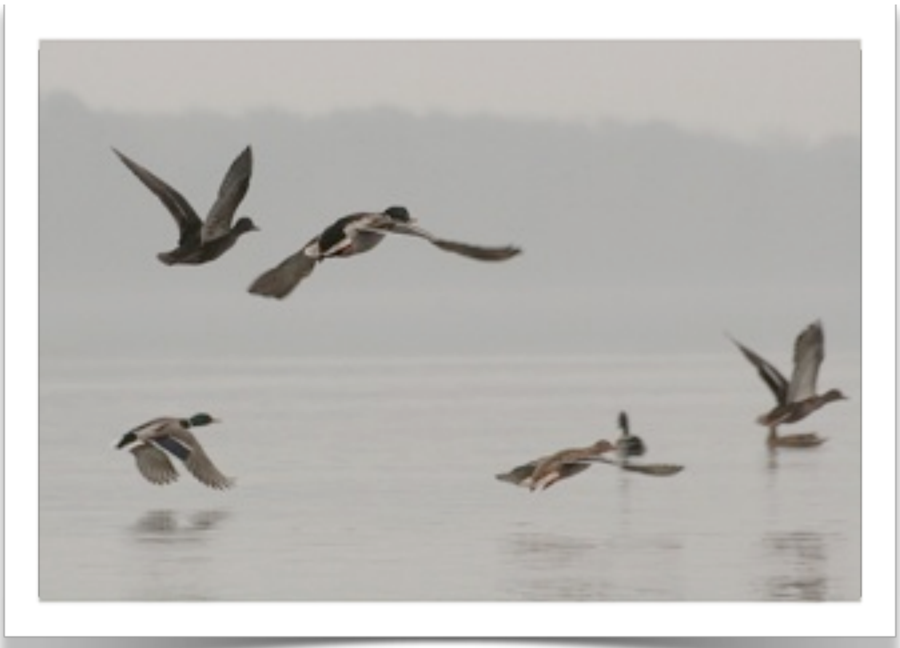

How to perform Activity 3: Dealing with relapse episodes successfully

"As detectives in your own backyard you are familiar with situations, thoughts, feelings, contexts and people that might restart your old habits. Now, after being the manager of your own behaviour you might have experienced some lapses and relapses. By these occuring you have more experience and are better equipped to use certain strategies that will minimise the potential to lapse and later relapse. However, this does not mean that it cannot occur."

"Please think back and identify the risk situations that fomented a lapse or relapse. Do not forget that these can include –see handouts session 5- (Marlatt & Donovan, 2005):"

1. *Negative emotional states* such as anger frustration, anxiety, depression, boredom). [Caused by intrapersonal perceptions of certain situations (e.g. feeling bored or lonely after coming home from work) or by reactions to environmental events (e.g. being made redundant)]
2. *Interpersonal conflict* (e.g. situations involving conflict associated with any interpersonal relationship, in particular family or friends).
3. *Social pressure* (e.g. situations in which you respond to the influence of another person or group of people who exert pressure for you to engage in a non desired behaviour).
4. *Positive emotional states* (e.g. celebrations), exposure to unhealthy eating -related stimuli or cues.

"Breaking these situations down in specific units, some possible factors and warning signs for Lapse/Relapse might be:

1. Hanging around with people that you know do not support your new behaviours
2. Isolating, or not seeking others for support (e.g. not using the telephone to seek support)
3. Keeping cookies, chocolates and other sweets around the house for any reason
4. Going shopping with a strong feeling of "starvation"
5. Relationship difficulties, ongoing serious conflicts (e.g. a spouse that boycotts your efforts for healthy eating)
6. Setting unrealistic goals (e.g. perfectionism, being too hard on yourself)
7. Changes in sleeping patterns, personal hygiene, or energy levels
8. Feeling overwhelmed, confused, useless, stressed
9. Constant boredom or irritability, or a lack of routine and structure in life
10. Major life changes like a loss or other painful emotions, or even winning the Euro Millions!
11. Ignoring lapse/relapse warning signs."

And ...Here are also some examples of situations that can restart unexpected cravings:

1. Something seen on TV or in a magazine
2. A smell
3. A sound that you might associate with your past habits
4. A particular feeling or mood

See ppt. slide 7

See ppt. slide 8

See ppt. slide 9

See ppt. slide 10

After sharing the information on the risk situations that brought lapses/relapses the participant will be prompted to list all the coping plans devised/developed/used across the previous month's for managing different risks.

Marlatt GA, Donovan,DM (Eds) (2005): Relapse Prevention: Maintenance strategies in the treatment of addictive behaviors

How to Protect yourself from Cravings that Come when you are alone?

"You should be aware of the fact that some of the risk situations, such as cravings, can happen when you are alone. Here are different ways of dealing with these feelings:

1. Call someone that his in a similar situation as yours. It is specially important to have support from a significant other that will be easily available any time of day or evening, who is very accessible and meets your specific needs. Sometimes sharing your (re) lapse experience with someone else will allow you to have some time to think of what went wrong.
2. Arrange to go out with some friends.
3. Go for a walk and clear your head. At the same time you will be fulfilling your activity goal!
4. Engage in something else that gives you pleasure (think about the rewards you have defined for yourself last week)
5. Make sure you do not have "junk food" or food that you do not want to eat around the house.
6. Be sure to stick to your daily eating plan, if you are not hungry is easier to fight these cravings, so do not skip any meals"

"Over time these feelings/cravings will weaken in intensity. But it is important to know that people who achieve long-term behaviour changes have learned to take the warning signals very seriously and to act upon them immediately."

Again the facilitator should state that idiosyncrasy is the key word, as noticed across the intervention programme! Hence, for each person lapse and relapse risk factors are unique to them, their lives, problems, goals and aims. By the end of this activity each person should have also reviewed coping strategies used to deal with a lapse, and prevent a relapse if the strategies used to avoid risk factors for lapse do not work.

See ppt. slide 11

Activity 4: Continue behaviour change and goodbye (20 minutes)

The facilitator will encourage the participants to continue using the strategies that have been learned across the sessions in their daily life, generalising them across different life situations. Each participant will be encouraged to use their pocket card (laminated card) since this contains all the strategies learned that can be easily remembered just by reaching their pocket.

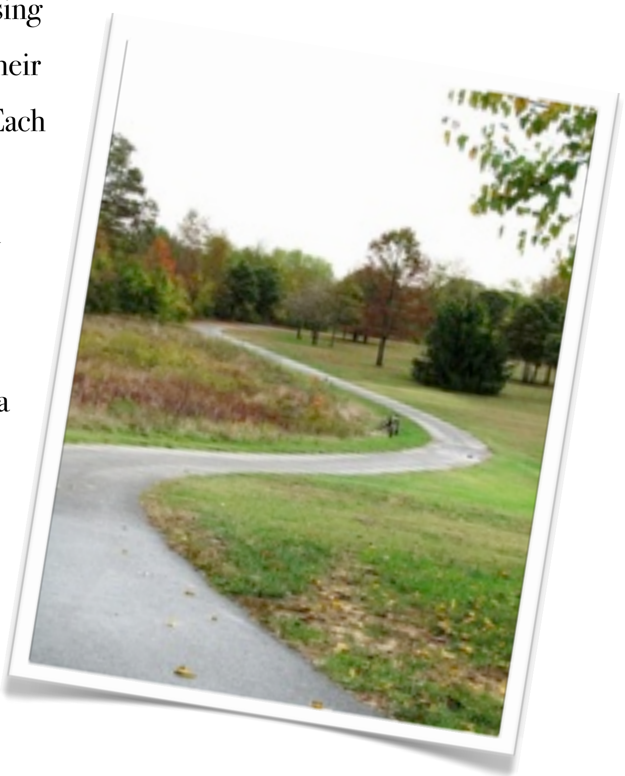

Again (like after session 5) this time should be used to give a general positive feedback and encouragement to the group members stressing that loosing weight and maintaining this loss is part of a new lifestyle, and a life task that, like life, is

filled with little pleasures and sometimes little pains. "Persisting in our own goals is the trick, knowing precisely what you want!".

To say good-bye allows the expression of feelings amongst the participants and amongst these and the group facilitator. The facilitator should, again, refer to the need to continuously deal with the possible obstacles and problems arising.

The facilitator will suggest that those group members who are available today could meet up afterwards, for example in the lobby or in a cafe to organise future gatherings and contact to keep up the support that has been established during the group sessions.

Activity 5: Life Challanges (10 minutes)

- Repeat to yourself every morning what are your plans.
- Observe what happens around you, and the impact on your own behaviour. Be aware, keep on self-monitoring your behaviour. When needed establish new plans and new protecting plans.
- Expect a lapse, but know that you have precise plans on how to deal with it in order to prevent a relapse.
- Believe that you can do it.
- Reinforce/reward yourself.
- Use these strategies in other areas of life, you will see they help!

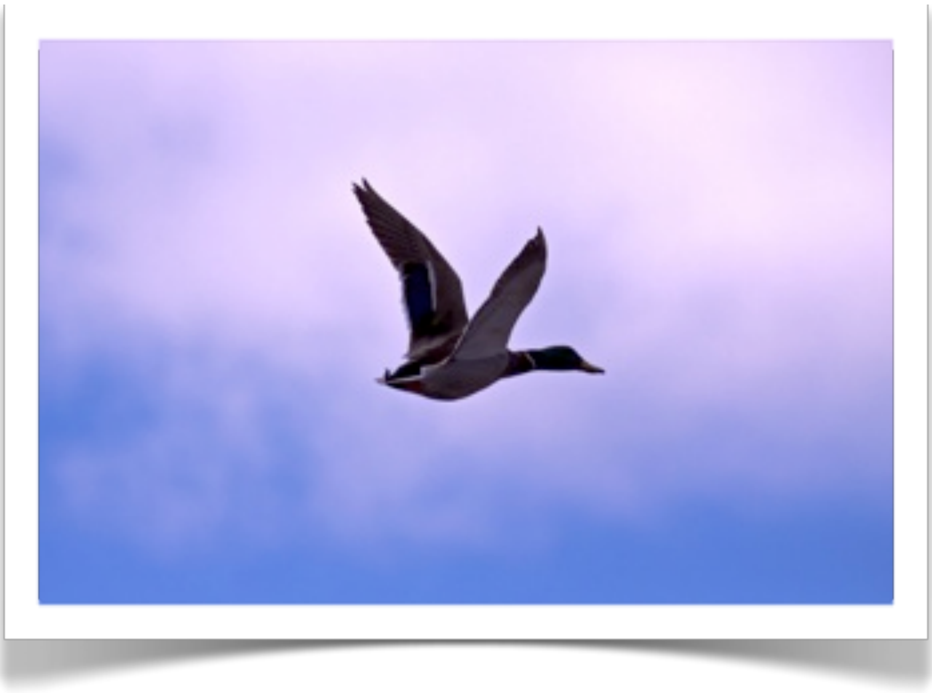

APPENDIX: SLIDES

Week 6

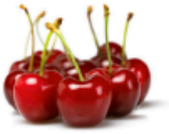

Reviewing achievements, generalising activities and promoting maintenance: the future starts now!

1

Today

1. Review of last weeks
   - Review achievements
   - Formulate a new eating goal
   - Formulate a new PA goal
   - Protecting eating and PA goals: overcoming the barriers and involving the facilitators
2. Become aware of relapse prevention issues.
3. The future starts now!

2

Review of last weeks

- How did you get on over the last weeks?
- Did you set yourself new eating and PA goals?
- How often did you achieve your eating and PA goals?
- Did you encounter any new barriers and/or facilitators?
- Did you reward yourself for achieving a goals?

3

Review of last weeks

- Team up with your neighbour and have a look at your achievements of last week
- Ask yourselves:
  - What did I try to do (what were my goals)?
  - How much effort did I invest to achieve my goals?
  - What impact/success did I have?
  - What benefits have I experienced?
  - What difficulties have I encountered?
  - How did I manage these difficulties?
  - What made it easier?
  - Am I satisfied with what I did during last week?
  - Am I getting enough support?
  - Do I want to keep this goal or do you want to change it?

4

Review of last weeks

- Report your experiences back to the group focusing on
  - Barriers
  - Facilitators
  - Successes
- Formulate new goals for eating and PA

5

Review self-rewards

- Have you rewarded yourself during the last weeks?
- Did you do it after achieving something?
- How did it work for you?
- Has this helped you achieve your goal?
- Have you planned your rewards for next week?

6

Reviewing relapse

- Remember that we said that if and when lapses happens:
  - Do not judge or blame yourself (remember that the process of change is full of ups and downs).
  - Take it as an opportunity to learn and prevent it from happening again.
  - The best thing to do if you have a lapse is to get right back on track towards your goals.
  - Regain control!

7

Relapse prevention

- Lapse or relapse can be triggered by *internal* (e.g. feelings or thoughts) and *external* (e.g. environment). These include:
- **Negative emotional states**, e.g. anger frustration, anxiety, depression, boredom. This is often caused by perceptions of certain situations (e.g. feeling bored) or by reactions to environmental events (e.g. being made redundant)
- **Interpersonal conflict**, e.g. situations involving conflict associated with any, in particular family or friends.
- **Social pressure**, e.g. situations in which you respond to the influence of another person or group of people who pressure you to engage in something not planned.
- **Positive emotional states**, e.g. celebrations and parties, exposure to unhealthy eating -related stimuli or cues.

8

Relapse prevention

Here is a list of typical situations that lead to lapses and relapses:

- Hanging around people that don't support your new behaviours.
- Isolating, or not seeking others for support.
- Keeping cookies, chocolates and other sweets around the house.
- Going shopping with a strong feeling of "starvation" .
- Relationship difficulties, ongoing serious conflicts (e.g. a spouse that boycotts your efforts for healthy eating).
- Setting unrealistic goals (e.g. perfectionism, being too hard on yourself)
- Feeling overwhelmed, confused, stressed.
- Constant boredom or irritability, or a lack of routine and structure in life
- Major life changes like a loss, or even winning the lottery!
- Ignoring lapse/relapse warning signs.

9

Relapse prevention

- Have you had in lapses or relapses over the last few weeks?
- Remember that having a **plan** on how to deal with warning events/risk factors that you can identify as risk situations will help you to cope with these.
- Besides this maintaining the behaviours you decided to change will allow you to increase your **confidence** and manage obstacles/barriers for keeping on track.

10

For all next weeks

- Keep up the good work!
- Continue use the strategies that you learned in this programme.
- Be healthy,
- Be happy,
- Have fun!

11

THANK YOU!

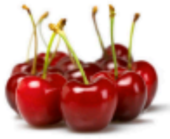

The research team wishes you the best of luck for your future.
Please remember to fill in our evaluation form so that we can continue to improve our programme

If you have any questions please email Stephan at s.u.dombrowski@abdn.ac.uk

12
